# An accelerated sine mapping whale optimizer for feature selection

**DOI:** 10.1016/j.isci.2023.107896

**Published:** 2023-09-14

**Authors:** Helong Yu, Zisong Zhao, Ali Asghar Heidari, Li Ma, Monia Hamdi, Romany F. Mansour, Huiling Chen

**Affiliations:** 1 College of Information Technology, Jilin Agricultural University, Changchun 130118, China; 2 Key Laboratory of Intelligent Informatics for Safety & Emergency of Zhejiang Province, Wenzhou University, Wenzhou 325035, China; 3 Department of Information Technology, College of Computer and Information Sciences, Princess Nourah bint Abdulrahman University, P.O. Box 84428, Riyadh 11671, Saudi Arabia; 4 Department of Mathematics, Faculty of Science, New Valley University, El-Kharga 72511, Egypt

**Keywords:** Natural sciences, Computer science, Engineering

## Abstract

An improved whale optimization algorithm (SWEWOA) is presented for global optimization issues. Firstly, the sine mapping initialization strategy (SS) is used to generate the population. Secondly, the escape energy (EE) is introduced to balance the exploration and exploitation of WOA. Finally, the wormhole search (WS) strengthens the capacity for exploitation. The hybrid design effectively reinforces the optimization capability of SWEWOA. To prove the effectiveness of the design, SWEWOA is performed in two test sets, CEC 2017 and 2022, respectively. The advantage of SWEWOA is demonstrated in 26 superior comparison algorithms. Then a new feature selection method called BSWEWOA-KELM is developed based on the binary SWEWOA and kernel extreme learning machine (KELM). To verify its performance, 8 high-performance algorithms are selected and experimentally studied in 16 public datasets of different difficulty. The test results demonstrate that SWEWOA performs excellently in selecting the most valuable features for classification problems.

## Highlights

• Several new strategies are introduced into the original WOA
• The proposed method has superior advantage over other peers
• The binary version is proposed for feature selection
• The new method was validated on publicly available datasets

## Graphical abstract

**Figure:**
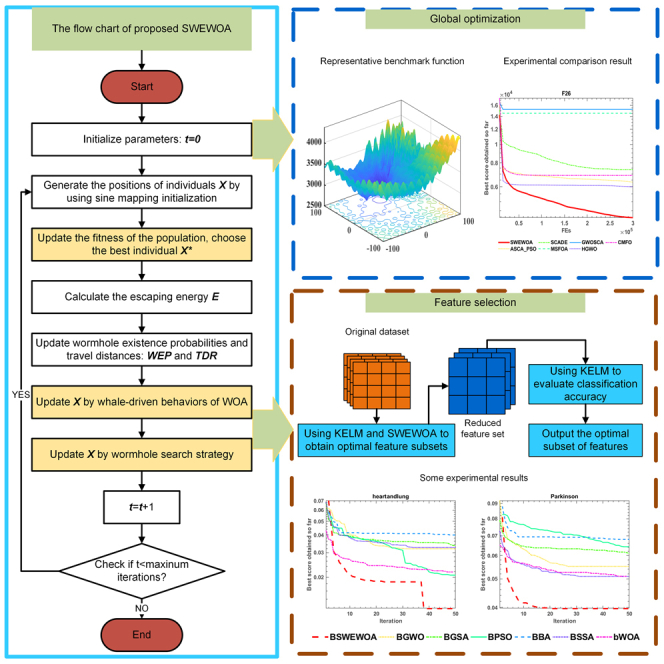

## Introduction

Feedforward neural networks are static nonlinear mappings that have gained widespread use caused of their capability to obtain complex nonlinear processing capabilities directly from the input samples. Over the last few years, gradient descent-based approaches, such as backpropagation methods, have been extensively employed in training feedforward neural networks.^1^ Nevertheless, this method usually has a slow learning speed or may quickly converge to the local optimal solution. To acquire better learning performance and overcome the difficulties of complex parameter adjustment in various applications, extreme learning machine (ELM) was put forward by Huang et al.,^2^ as an excellent new learning algorithm for feedforward neural networks. It has been extensively concerned by scholars because of its fast learning ability, excellent generalization ability, and few tuning parameters, and it has been utilized to tackle a variety of realistic issues, including image classification,^3^ face recognition,^4^ wind power probability prediction,^5^ and building energy consumption estimation.^6^ Although ELM has an excellent performance in practical applications, it will show instability in some cases because the input layer weight and hidden layer bias are randomly selected. To overcome the above difficulties, Huang et al.^7^ integrated kernel function into ELM and proposed kernel extreme learning machine (KELM). KELM can make better predictions while keeping the advantages of ELM.

Since the introduction of KELM, KELM has been commonly employed in various situations because of its stronger robustness, including medical diagnosis,^8^^,^^9^^,^^10^ aircraft engine fault diagnosis,^11^ financial stress prediction,^12^ bankruptcy prediction,^13^ classification of hyperspectral remote sensing images,^14^^,^^15^^,^^16^ intrusion detection,^17^ activity recognition,^18^ two-dimensional contour reconstruction,^19^ foreign fiber recognition in cotton,^20^ and many other scenarios. However, in practice, the choice of kernel parameter *γ* and penalty parameter *C* in KELM will seriously affect the classification accuracy of KELM. Therefore, to handle the optimization issue of the KELM parameter setting, a meta-heuristic algorithm was utilized to cope with this problem.^21^ It is worth noting that there are always redundant features or irrelevant features in most datasets that are not helpful to the learning task, and these features may affect the model’s performance. Studies have shown^22^ that an excellent feature subset does well for the capacity of the model. Accordingly, it is necessary to select features before model construction.

Feature selection is a crucial step in feature engineering. In practical problems, an object often has many features; these features are roughly categorized into three types: related features that can improve the effectiveness of learning algorithms, irrelevant features that do not change the algorithm’s performance, and redundant features that can be inferred from other features.^23^ Nevertheless, for a specific learning method, it is unknown which feature is practical and which will significantly influence the accuracy of the model and the amount of calculation. Consequently, screening the related features is crucial to the learning algorithm’s performance. The process of removing irrelevant and unnecessary features to obtain the most relevant subset of features is feature selection. The methods of feature selection are subdivided into the filter, embedded, and wrapped. Each of the three methods has its advantages, so choosing an appropriate feature selection method is not an easy problem.^24^

Filtering feature selection is to score each feature by correlation to represent the importance of the feature and then filter the feature according to the set threshold or the number of features to be chosen. The method does not rely on any machine learning method, does not require training, and is computationally efficient. Therefore, this method can quickly and efficiently remove redundant features from large-scale datasets. Ke et al.^25^ developed a standard fusion filtering feature selection approach for gene microarray data. Cui et al.^26^ presented a filtering one based on relief. Hancer et al.^27^ introduced information theory and feature ranking into the filtering feature selection technique.

Embedded-based methods comprehensively consider feature selection and model training. This approach automatically selects features during the training procedure. Li et al.^28^ presented an embedded feature selection technique based on an approximate marginal likelihood correlation vector machine. Zhu et al.^29^ developed a discriminative embedded unsupervised feature selection used to process high-dimensional datasets.

Wrapper-based approach evaluates the feature subset according to the performance of the model, aiming to obtain a feature subset "tailored" for the model.^30^^,^^31^^,^^32^ Compared with the filtering model, the packaging model is more specific to the model even though the computational cost is larger, and its classification performance far exceeds that of the filtering model. The wrapped one has higher computational efficiency and classification accuracy than the embedded model.^33^ Therefore, the wrapped approach is an excellent choice when time-consuming issues can be ignored, and the model is to be obtained as accurately as possible. However, the wrapped feature selection approach must search for the best subset of features over a wide feature space. If the exhaustive method selects the optimal feature subset, the computational overhead is too high, and it is inappropriate for solving the feature selection problem with a large search space. Recently, heuristic algorithms have emerged as a hot topic for scholars to solve optimization problems because of their simple structure and strong optimization ability. Studies in^34^^,^^35^ showed great success was achieved using a heuristic algorithm to obtain the model’s key parameters and then perform feature selection. Therefore, using the heuristic algorithm to search in complex feature space, the wrapper-based approach is a pretty good alternative.

There are different optimization methods available, which can be categorized based on their ability to handle cost functions with many or multiple objectives.^36^^,^^37^ Most of these methods fall under the single objective domain, meaning they can only handle one objective at a time.^38^^,^^39^ According to the survey, many classical approaches and new ones have been developed and widely used in many fields, such as ant colony optimization (ACO),^40^ differential evolution algorithm (DE),^40^ particle swarm optimization (PSO),^41^ tunicate swarm algorithm (TSA),^42^ Harris hawks optimization (HHO),^43^ gray wolf optimizer (GWO),^40^ fruit fly optimization algorithm (FOA),^44^ grasshopper optimization algorithm (GOA),^40^ multi-verse optimizer (MVO),^40^ gravitational search algorithm (GSA),^40^ firefly algorithm (FA),^40^ moth-flame optimization (MFO),^40^ slime mould algorithm (SMA),^45^ simulated annealing algorithms (SA),^40^ sine cosine algorithm (SCA),^40^ hunger games search (HGS),^46^ weighted mean of vectors optimizer (INFO),^47^ Runge Kutta optimizer (RUN),^48^ and colony predation algorithm (CPA).^49^ At the same time, some improved algorithms have been proposed to deal with the more difficult optimization situations, for example, Issa et al. suggested an adaptive SCA integrated with PSO (ASCA_PSO)^50^ achieves convergence accuracy and speed improvement. Nenavath et al. developed a hybridizing SCA with DE (SCADE)^51^ to speed up the convergence of standard SCA and DE. Zhang et al. suggested a new FOA based on a multi-scale cooperative mutation strategy (MSFOA)^52^ addresses the limitation that standard FOA easily traps in local optima. Singh et al. introduced the SCA into the GWO (GWOSCA)^53^ to obtain higher-quality solutions. Zhu et al. used DE to improve the disadvantage that GWO is prone to stagnation (HGWO),^54^ and Li et al. presented a chaos-enhanced moth-flame optimization (CMFO)^55^ to strengthen the convergence speed and precision of CMFO.

The whale optimization algorithm (WOA)^56^ is currently one of the most popular swarm intelligence algorithms (SIA) in research, inspired by the predation activity of humpback whales in nature. Its main structure is a PSO-based method, in which a global best tries to lead other members of a swarm.^57^ Because of its uncomplicated structure, fewer parameters, and great optimization ability, WOA has been widely used by scholars to cope with optimization problems. However, the complexity of optimization problems is increasing day by day. In particular, feature selection needs to dig the best subset of features in the complex feature area, and the original WOA cannot meet the needs of real complex problems well. Therefore, the improved algorithm of WOA has become a research hotspot. For example, Yousri et al. used chaotic mapping to accelerate the convergence rate and execution time of WOA (CWOA).^58^ Elhosseini et al. considered the imbalance between exploration and exploitation in the WOA, so two dynamic parameters A and C were introduced into WOA to propose the ACWOA.^59^ Sun et al. also considered the imbalance between algorithm exploration and exploitation, so they presented multi-strategy enhanced WOA (MWOA). They introduced a nonlinear dynamic strategy into WOA. In addition, the Lévy-flight strategy prevents MWOA from falling into a local optimum. Abd Elaziz et al. developed an improved WOA based on oppositional learning (OBWOA),^60^ which uses oppositional learning methods to enhance exploration in the search space. The practice proves that OBWOA can improve convergence accuracy effectively. In a nutshell, most researchers have introduced corresponding strategies to solve the problem that WOA itself is prone to trapping into local optimum and the problem that the exploration and exploitation are imbalanced. However, their methods still have the potential to improve.

These heuristics and improved algorithms have demonstrated significant potential in many application scenarios, such as engineering design problems,^61^^,^^62^^,^^63^ image segmentation,^64^^,^^65^^,^^66^^,^^67^^,^^68^ scheduling problems,^69^ feature selection,^70^^,^^71^^,^^72^ and financial stress prediction.^21^^,^^73^ Many practices indicate that the enhanced approach performs better than the original algorithm in some optimization domains. Nevertheless, the "No free lunch" (NFL) theorem^74^ suggests that no single algorithm can ideally face all optimization situations, which shows that although various improved algorithms of these proposed original algorithms are significantly superior to the original algorithm for specific problems, this is not necessarily the case for other optimization domains. Therefore, in the process of solving specific problems, they may be prone to low convergence accuracy, trapping by the local optimal, and may not be able to get satisfactory results. Studies have shown that,^75^^,^^76^ due to the weak exploration capability of the original WOA, a larger proportion of the entire search process is utilized, which may result in low convergence precision of WOA and trapping in the local optimum. Therefore, to deal with these problems and effectively improve the performance of the machine learning feature selection model, this paper innovatively uses a sine mapping initialization strategy, escape energy, and wormhole search strategy to enhance the WOA(SWEWOA). Then, a binary version of BSWEWOA based on SWEWOA is developed and used to solve the feature selection problem. Eventually, a new machine learning model is proposed by combining KELM and BSWEWOA. To prove the superiority of the proposed SWEWOA, experiments are conducted in two competition sets, IEEE CEC2017^77^ and IEEE CEC2022. The results are analyzed by two statistical methods, including Wilcoxon signed rank test (WSRT)^78^ and the Friedman test (FT),^79^ to verify the global optimization performance of SWEWOA. Regarding feature selection, 13 public datasets and different performance indicators were used to demonstrate the feature selection ability of BSWEWOA-KELM. The test outcomes reveal that compared with other KELM models, the presented BSWEWOA-KELM model has better classification results and robustness. It is an excellent machine learning tool. The primary contributions of the paper are as follows:

(a) In the population initialization stage of SWEWOA, the sine mapping initialization strategy was proposed to replace the original random generation strategy, which improved the quality of the initial solution in WOA and provided a good direction for the subsequent search of whales.
(b) The wormhole search mechanism is proposed to enhance the convergence accuracy of SWEWOA and to keep it from dropping into a local optimum.
(c) Finally, it is proposed to introduce escape energy to guide whales to make more reasonable behaviors, give SWEWOA more exploration opportunities, and strengthen the global search capability of SWEWOA.
(d) Among the 42 test functions of IEEE CEC2017 and CEC2022, SWEWOA outperforms other well-known original algorithms and advanced improved algorithms to prove that SWEWOA is a competitive optimizer, and the improved strategy in this paper can also provide new ideas for the improvement of other meta-heuristic algorithms.
(e) In this paper, we combine BSWEWOA (binary version of SWEWOA) and KELM to develop a new machine learning feature selection model BSWEWOA-KELM, and it is compared with other six excellent swarm intelligence algorithms-based KELM models on 13 public datasets. The capacity of the proposed model in high-dimensional datasets is also analyzed. The results indicate that the classification accuracy of the new model is higher, so this work can be used as an effective tool for decision-making tasks.

The remainder of the paper is as follows: in the Method details section, we present the specific details of SWEWOA and the materials used. The results and discussion of the global optimization experiments and feature selection experiments are presented by Results and discussion section. Finally our conclusions and perspectives for the future are given in the Conclusions and future works section.

## Results and discussion

### All models used in the experiment

In this section, we list all the models used in this study and their specific details.

### Experimental settings

All the experiments in Section 4.2 are based on thirty IEEE CEC2017 test functions. The main goal is to prove that SWEWOA has high performance. The specific description of test functions is described in Table B1 of the supplemental information.

In order to prove the superiority of SWEWOA, firstly, the strategy combination comparison experiment, stability analysis experiment, experimental balance-diversity assessment, and search history assessment are carried out on SWEWOA. Then, SWEWOA was compared with eight original classical algorithms, 12 WOA variants, and other high-performance variants. The original algorithms include HHO, TSA, FA, PSO, SCA, MFO, SMA, and WOA. The variant algorithms include CWOA, BMWOA, CCMWOA, ACWOA, MWOA, OBWOA, ASCA_PSO, SCADE, MSFOA, GWOSCA, HGWO, and CMFO. To ensure the fairness^80^^,^^81^ and reliability of the test, the evaluation times rather than the iteration times are employed to prove that SWEWOA does not improve the optimization capability by heaping strategies, and the experimental parameters in the relevant experimental process are disclosed uniformly. Table B2 describes the parameters required for the experiment. In addition, the detailed settings of the competitors for the global optimization and parameter settings of the binary version algorithms are depicted in Tables 1 and 2.

**Table 1:** Specific settings for all algorithms in the global optimization experiment

Method	Specific parameters
SWEWOA	b=1; ${WEP}_{\min}=0.2;{WEP}_{\max}=1$
HHO	$E_{0}=\left[-1\;1\right]$; β=1.5
TSA	ST=0.1
FA	$\alpha =0.5;\;\beta_{\min}=0.2;\;\gamma =1\text{;}$
PSO	$c_{1}=2;\;c_{2}=2;\;V_{\max}=6$
SCA	a=2
MFO	b=1
SMA	z=0.03
WOA	$a_{1}=\left[2\;0\right];\;a_{2}=\left[-2-1\right];\;b=1$
CWOA	a=[20];b=1；c=5
BMWOA	β=0.005;bw=0.5
CCMWOA	m=1500;
ACWOA	w=[0.50.75];b=1
MWOA	b=1
OBWOA	b=1
ASCA_PSO	$M=4;\;N=9;\;V_{\max}=6;\;w_{\max}=0.9;\;w_{\min}=0.2$
SCADE	$\beta_{\min}=0.2;\beta_{\max}=0.8;pcr=0.8;a=2\text{;}$
MSFOA	$w_{0}=1;\;\alpha =0.95;M=5;\;W=200;\;n=0.005$
GWOSCA	a=2;r=[02π]
HGWO	$F=1;\;P_{c}=0.2$
CMFO	CC(1)=0.7;ccnum=1
WDNMWOA	$b=1;r_{4}\in \left[0,1\right];r_{5}\in \left[0,1\right]$
BWOA	b=1;m=2500
FSTPSO	$V_{\max}=6;V_{\min}=-6;c_{1}=2;c_{2}=2$
DHHOM	a=2.5,SF=0.5
GWO	$r_{1}\in \left[0,1\right],r_{2}\in \left[0,1\right]$
BA	Qmin=0,Qmax=2

**Table 2:** Parameter settings of the binary version algorithms

Methods	Other parameters
BSWEWOA	a=randomin(0,4];p=6;k=randomin[0,1]; ${WEP}_{\min}=0.2;{WEP}_{\max}=1$
BGWO	a=[20]
BWOA	$a_{1}=\left[2\;0\right];\;a_{2}=\left[-2-1\right];\;b=1$
BPSO	$w=1\;;c_{1}=2;c_{2}=2$; $V_{\max}$*= 6*
BBA	$A=0.5;\;r=0.5；Q_{\min=0}；Q_{\max=2}$
BGSA	$Rnorm=2;\;Rpower=1;a=20;\;G_{0}=100$
BSSA	b=1
BSCGWO	a=[20];q=2
BMFO	$a_{1}=\left[0\;1\right];\;a_{2}=\left[0\;1\right];\;b=1$

The results of the competitor comparisons were validated by WSRT and FT. Among them, p value is applied to evaluate the variability between competitors. The p value less than 0.05 suggests a significant difference in both methods. However, the difference between the competitors cannot be determined only through the significance test, so in this paper, "+" means that SWEWOA has better performance than this algorithm, "−" means that SWEWOA is weaker than this algorithm, and " = " indicates that the capability difference between the competitor is small.

### Global optimization experiment

The proposed SWEWOA is formed based on WOA by introducing three strategies: sine mapping initialization, wormhole search strategy, and escape energy. WOA is improved to have a more efficient initial solution and the capacity to extricate from local optimum and improves the convergence accuracy of SWEWOA. This section confirms the superiority of SWEWOA through experiments in the following subsections.

#### The impact of three strategies

To verify that the introduction of sine mapping initialization strategy, wormhole search strategy, and escape energy benefits the performance of SWEWOA, three improved strategies are introduced to construct eight different WOA variants, and the constructed variant algorithm is used in the policy comparison experiment. The eight different WOA and variants introducing the three strategies are shown in Table 3. Where "SS" stands for sine mapping initialization strategy, "WS" stands for wormhole search strategy, and "E" stands for escape energy. In addition, "1" and "0" represent used and unused strategies, respectively.

**Table 3:** The combination scheme of the three strategies

Methods	SS	WS	E
SWEWOA	1	1	1
WOA_S	1	0	0
WOA_W	0	1	0
WOA_E	0	0	1
WOA_SW	1	1	0
WOA_SE	1	0	1
WOA_WE	0	1	1
WOA	0	0	0

Tables 4 and 5 show the WSRT and FT outcomes of the eight combined variant competitors on the thirty test functions of CEC2017, respectively. From the results in Tables, it is not difficult to see that the WSRT ranking and FT ranking of the original WOA without any strategy are in last place. This indicates that the three introduced strategies can enhance the competitiveness of WOA. SWEWOA ranked first in the two statistical methods, and WSRT ranked 2.00 and FT ranked 2.33, respectively. This indicates that only when these three strategies are simultaneously combined and introduced into WOA can the optimization performance attain the strongest.

**Table 4:** Comparison of strategy combination based on WSRT

Methods	+/−/=	Mean	Rank
SWEWOA	∼	**2.00**	**1**
WOA_S	28/0/2	6.53	7
WOA_W	22/0/8	3.60	4
WOA_E	27/0/3	6.00	6
WOA_SW	20/1/9	3.17	3
WOA_SE	27/1/2	4.93	5
WOA_WE	4/2/24	2.30	2
WOA	29/0/1	7.47	8

**Table 5:** Comparison of strategy combination based on FT

Methods	Mean	Rank
SWEWOA	**2.33**	**1**
WOA_S	6.30	7
WOA_W	3.92	4
WOA_E	5.61	6
WOA_SW	3.63	3
WOA_SE	5.08	5
WOA_WE	2.46	2
WOA	6.67	8

#### The historical search process experiment

This subsection discusses the characteristics of the SWEWOA through search history experiments and balanced diversity experiments.

**Figure 1:**
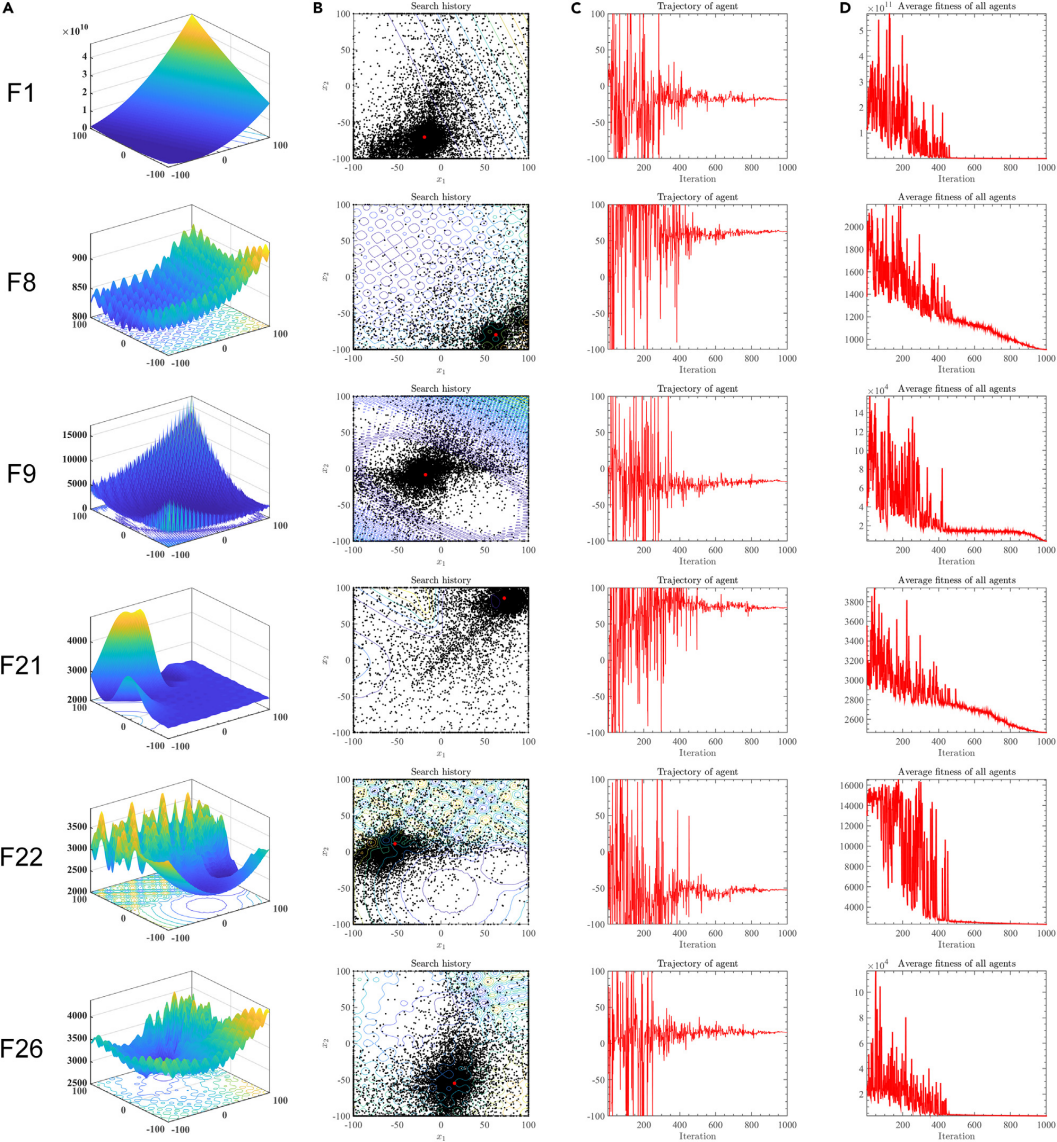
Historical search analysis for SWEWOA (A) 3D model of the partially test function. (B) Record of historical positions. (C) Search trajectories in the first dimension. (D) Average fitness value of the population.

Figure 1 shows the historical search trajectory of SWEWOA, where Figure 1A is the 3D model of the objective function. Figure 1B displays the historical search trajectory of SWEWOA in the search region. The red dot stands for the location of the global optimal solution, and the other black dots indicate the historical location of the whole individuals in 1000 iterations. It is not difficult to see from Figure 1B that search agents uniformly search in solution space. Most individuals mainly search around the global optimal solution. In Figure 1C, the fluctuation of the entire population of SWEWOA is relatively drastic at the beginning of the iteration and gradually becomes stable with the progress of the search. Figure 1D draws the change of the average fitness. At the beginning of the iteration, the fitness is large because the search agents are allocated to the feasible region. However, as the search progresses, the algorithm tends to search in a small local space. Finally, the overall average fitness value becomes smaller.

**Figure 2:**
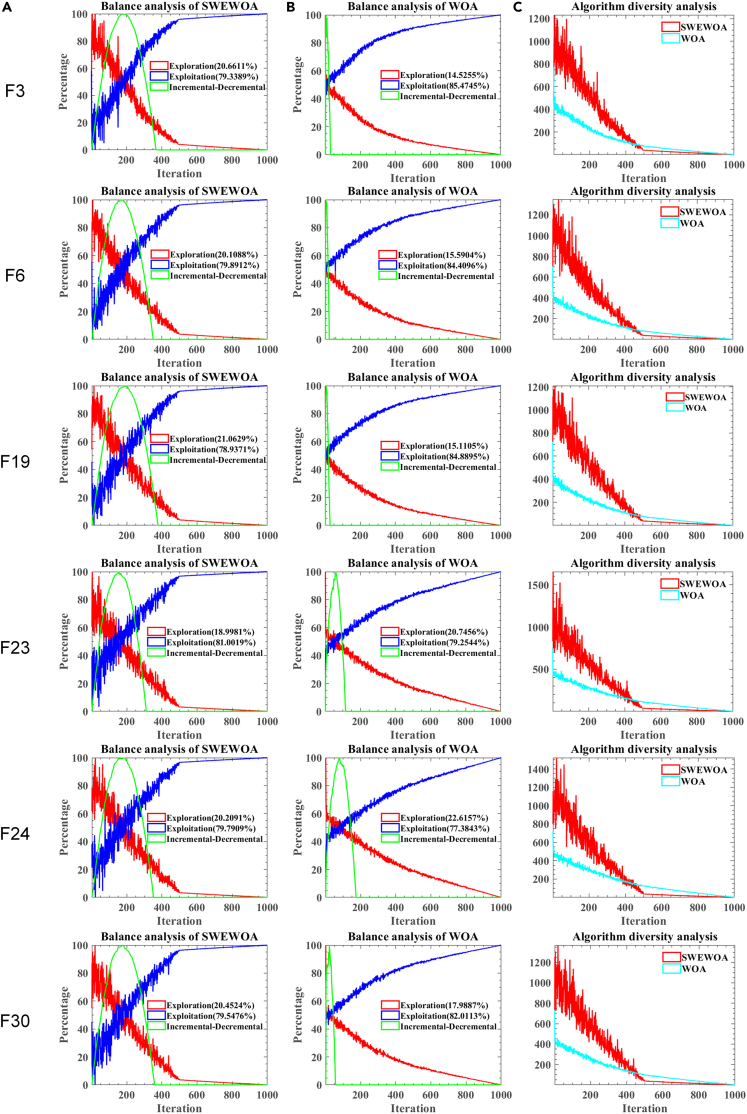
Balance and diversity analysis of algorithms (A) Balance of SWEWOA. (B) Balance of WOA. (C) Diversity of SWEWOA and WOA.

To further analyze the influence of the introduced mechanism on the exploration and development effect of the original WOA, this paper conducted 1000 iterations of comparison experiments on the balance and diversity of SWEWOA and WOA algorithms. Figure 2A and 5B consist of three curves, including the red line, blue line, and green line. The red and blue lines play a part in the proportion of exploration and exploitation in the overall search process. The green line is the incremental-decremental curve. The rising incremental-decremental curve indicates that exploration is stronger than exploitation at this time, which means that the algorithm is more concerned with global search in the solution space. Otherwise, the incremental-decremental curve shows a downward trend. In this case, the algorithm pays more attention to local search near the historical solution. The green line reaches its maximum value when the proportion of exploration and exploitation phases is equal. Figure 2A shows that SWEWOA increases opportunities in the exploration phase at the beginning of the iteration and focuses more on development at the end; this is due to the introduction of escape energy of prey E. At the beginning of the iteration, the energy of prey is abundant. At this time, it is not an excellent option to attack directly, so we choose to surround the prey and gradually consume the energy of the prey. Figure 2B demonstrates that the original WOA has paid attention to the local search for a long time, so the original WOA has a high probability of dropping into the local optimum. As seen from functions F3, F6, F19, and F30, global search capability of SWEWOA has been enhanced. As can be seen from functions F23 and F24, the local search ability of SWEWOA is enhanced. Figure 2C is the diversity image of the search agent, which reflects the diversity change of the population through the average distance between individuals in the population. Figure 2C shows that the SS is utilized in the beginning phase instead of random initialization, making SWEWOA more diverse. In addition, in the beginning phase, the population diversity of SWEWOA fluctuates wildly, which is why the algorithm gives more opportunities to the exploration stage. Then, with the increase in iteration times, the diversity of the SWEWOA swarm gradually decreased. SWEWOA is more inclined to perform a local search.

#### The experimental analysis of stability in various dimensions

To meet the needs of practical problems, the capability of algorithms to perform in different dimensions is also a significant index to judge the optimization competence of the approach. In this subsection, the optimization results of SWEWOA and WOA in four dimensions are compared to estimate the optimization capacity of SWEWOA. The dimensions of the question are 10, 30, 50, and 100, respectively. Table C1 of the appendix presents the comparison consequences of two methods, among which SWEWOA is dominant in terms of the number of the optimal mean and standard deviation, which denotes that SWEWOA has better optimization ability than WOA in four different dimensions. For further demonstrating that SWEWOA has stronger optimization ability than WOA, Table C2 in the appendix displays the comparison consequences of WSRT of SWEWOA and WOA, and when p value <0.05, it means that the capability of SWEWOA and WOA is significantly different. In the “result” of table, "+" indicates SWEWOA is stronger than WOA, "−" is the opposite, and " = " means that the two competitors have the same performance. “B” represents the number of functions in which SWEWOA has an advantage, “W” stands for the number of poor SWEWOA functions, and “E” plays the part of the number of functions for which WOA and SWEWOA are close to the same. Table C2 in the appendix demonstrates that there are only five p values >0.05, and the other p values <0.05. This shows that there is a significant difference between the two approaches, and the SWEWOA has a better optimization impact. Tables 6 and 7 illustrate the WSRT and FT results of the two algorithms in four dimensions. In summary, SWEWOA achieves better optimization results than WOA on 30 benchmark functions in four dimensions; this suggests that SWEWOA performs more consistently and better across different dimensions.

**Table 6:** WSRT results in four dimensions

Dim	10	10	30	30	50	50	100	100
Methods	SWEWOA	WOA	SWEWOA	WOA	SWEWOA	WOA	SWEWOA	WOA
Mean_rank	**1.00**	2.00	**1.03**	1.97	**1.03**	1.97	**1.00**	2.00
Rank	**1**	2	**1**	2	**1**	2	**1**	2

**Table 7:** FT results in four dimensions

Dim	10	10	30	30	50	50	100	100
Methods	SWEWOA	WOA	SWEWOA	WOA	SWEWOA	WOA	SWEWOA	WOA
Mean_rank	**1.06**	1.94	**1.05**	1.95	**1.06**	1.94	**1.05**	1.95
Rank	**1**	2	**1**	2	**1**	2	**1**	2

#### The comparison between SWEWOA and original algorithms for IEEE CEC2017

To prove the superiority of SWEWOA more comprehensively, SWEWOA is compared with eight well-known high-performance original algorithms, including HHO, TSA, FA, PSO, SCA, MFO, SMA, and WOA.

**Figure 3:**
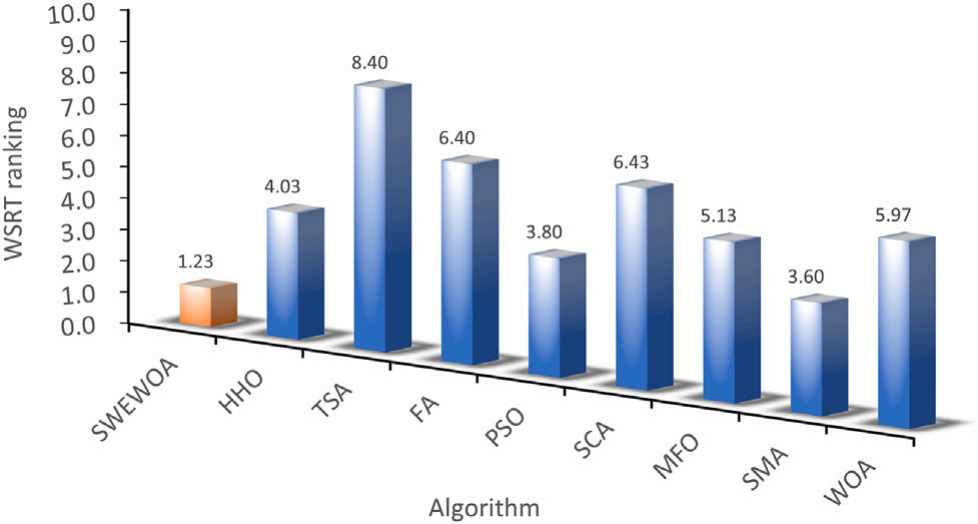
WSRT ranking of SWEWOA and original algorithms

**Figure 4:**
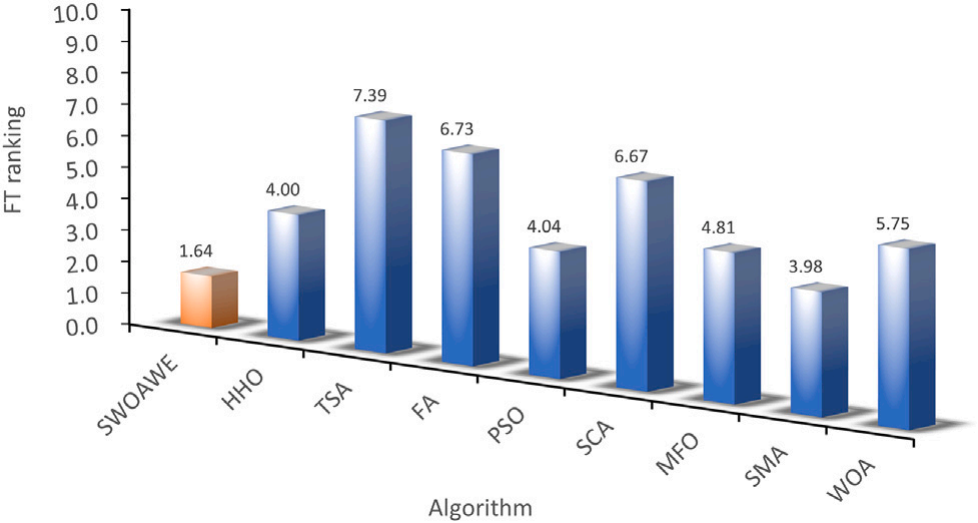
FT ranking of SWEWOA and original algorithms

**Figure 5:**
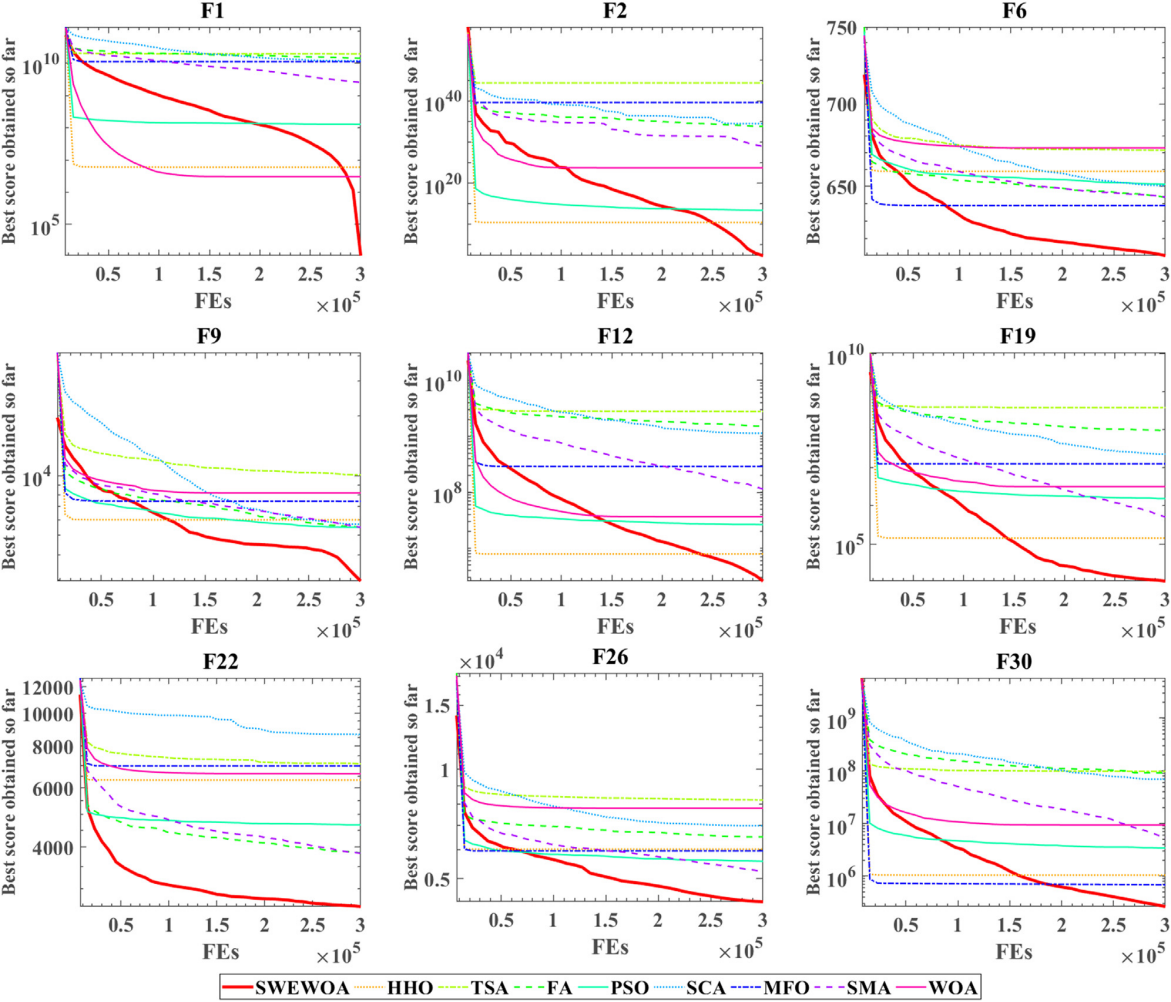
Convergence curve of the comparison between SWEWOA and original algorithms

The mean and standard deviations of the aforementioned nine algorithms are expressed in Table C3. In average results, SWEWOA performs best on 23 functions. In the standard deviation, the optimal number of standard deviations SWEWOA, although a less optimal average number, but compared with other 8 kinds of the algorithm, the optimal number of standard deviations SWEWOA is still the highest. This shows that SWEWOA has the most stable experimental results in thirty independent runs. The results of the different significance between the eight algorithms and SWEWOA are given in Table C4. The experimental outcomes show that compared with SWEWOA, the p value of the eight original competitors is less than 0.05 on most of the functions, and the result is "+," which indicates that SWEWOA is significantly different from the other eight famous original competitors on most of the test functions. The optimization capability of SWEWOA on most functions is the best among these 9 algorithms. Figures 3 and 4 show the WSRT and FT rankings of the nine algorithms, respectively. It can be seen that SWEWOA ranks first among the two evaluation methods, revealing that SWEWOA has the strongest optimization capability over the nine competitors. The WSRT and FT rank of WOA are both sixth. The results point that WOA itself has good optimization capacity, but the optimization ability of WOA is significantly improved after the introduction of the three strategies. The partial convergence curves for the nine competitors are shown in Figure 5. As displayed in the figure, the convergence curve of SWEWOA is at the bottom, which means that SWEWOA is the highest among the 9 algorithms in terms of convergence accuracy. Although the convergence speed of SWEWOA is not the best, the exploration ability of SWEWOA is stronger than that of the other 8 algorithms, so it can search for more excellent solutions. In addition, SWEWOA can better escape from the local optimal and keep the algorithm with certain global search in the later phase. In general, the SWEWOA has advantages in the comparison experiments with the aforementioned classical and new algorithms.

#### The comparison of WOA variants for IEEE CEC2017

In this part of the experiment, to further verify the performance, SWEWOA is compared with six improved techniques of excellent WOA. These algorithms include CWOA, BMWOA, CCMWOA, ACWOA, MWOA, and OBWOA.

**Figure 6:**
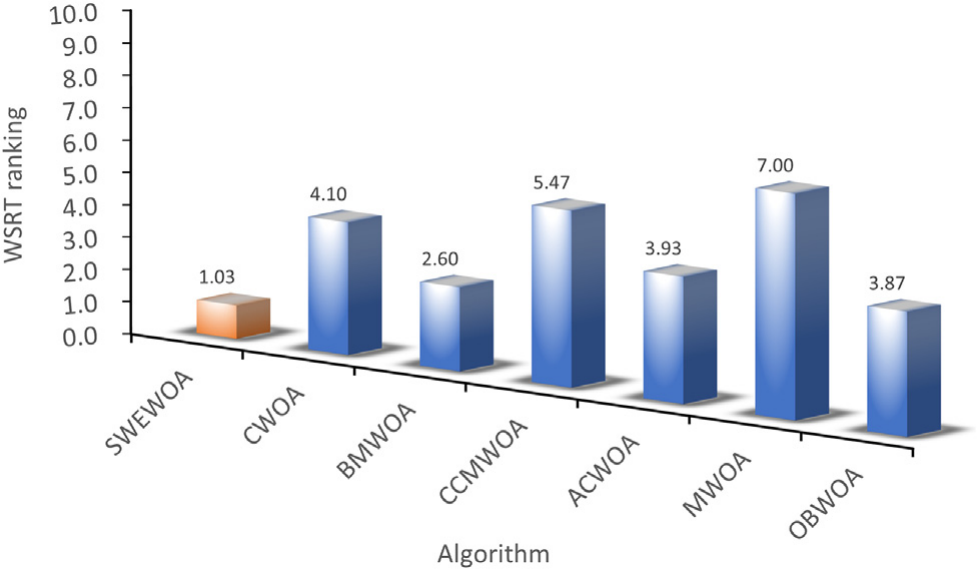
WSRT ranking of the WOA variant algorithms

**Figure 7:**
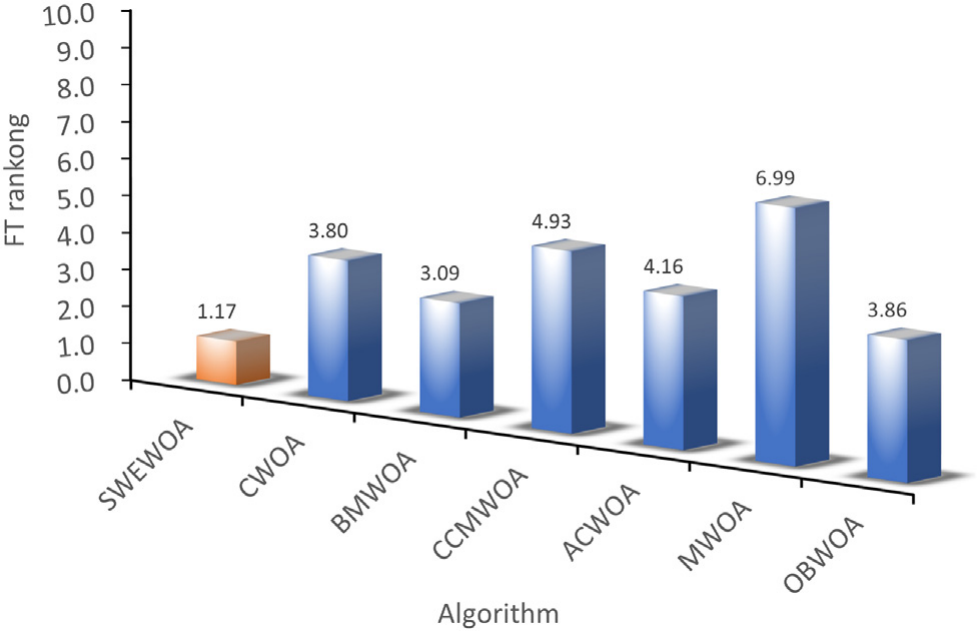
FT ranking of the WOA variants

**Figure 8:**
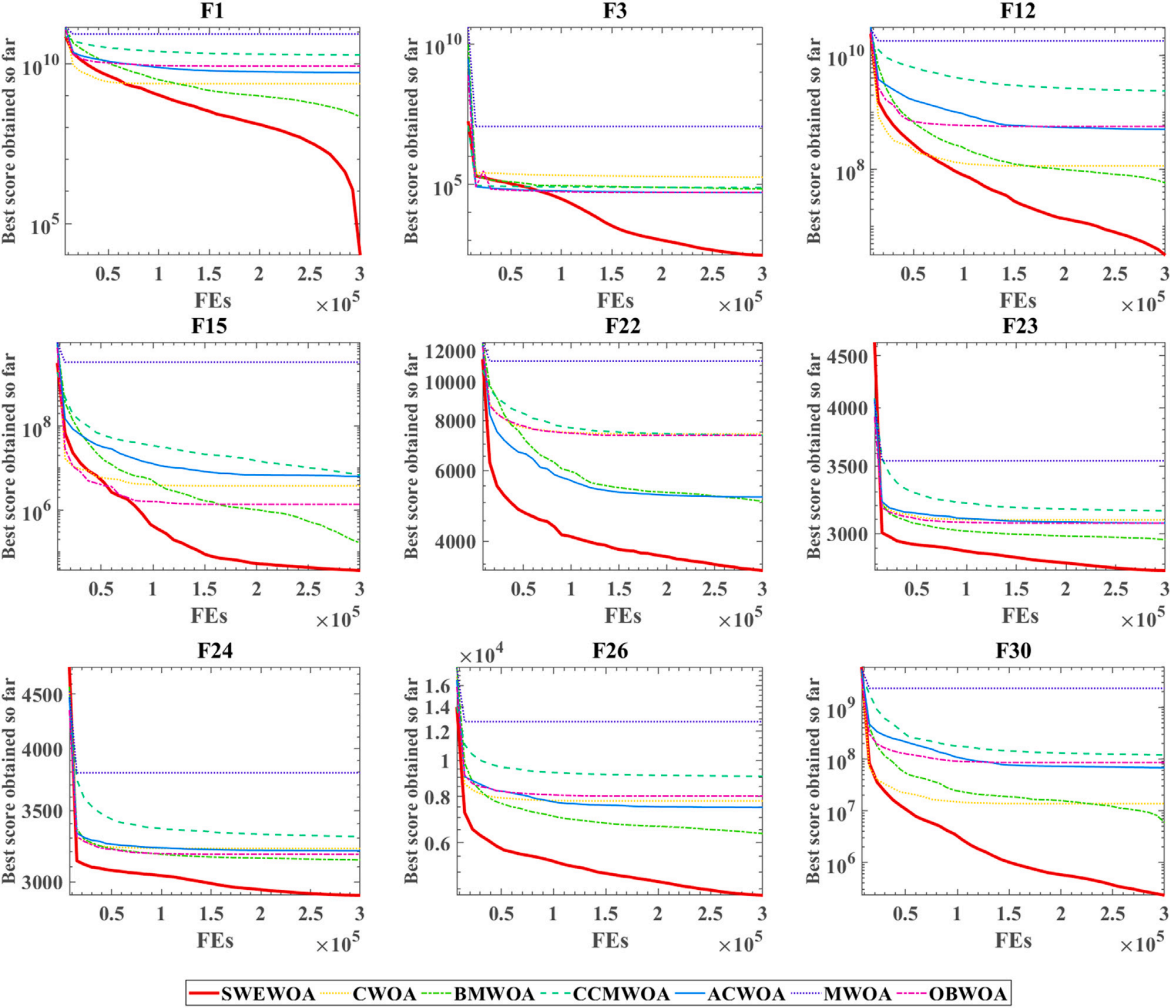
Convergence curve of the WOA variants

Table C5 expresses the mean and standard deviation of the comparison outcomes of thirty test functions of the seven WOA variants in IEEE CEC2017. In Table C5, the number of best average value and standard deviation of SWEWOA are 29 and 22, respectively. SWEWOA ranked first in both criteria. This suggests that the overall capability of SWEWOA is stronger than the other six improved variants of WOA. The outcomes of the difference comparison between SWEWOA and the six different WOA variants are presented in C6. Table C6 demonstrates that there are only three p values greater than 0.05, whereas the other p values are all less than 0.05, indicating significant differences between the six improved WOA algorithms and SWEWOA in most functions. Meanwhile, from the perspective of the number of "+", SWEWOA has the largest number of "+", which indicates that SWEWOA has better optimization ability than other algorithms. The number of "−" to 0 demonstrates that SWEWOA in 30 test functions of performance is not weaker than the other 6 kinds of algorithms. In addition, compared with CCMWOA, ACWOA, MWOA, and SWEWOA, the number of "+" is all 30, which indicates that SWEWOA performs better than the three algorithms in IEEE CEC2017. Figures 6 and 7 illustrate the WSRT and FT results of the seven algorithms. The average rank of WSRT and FT of SWEWOA is 1.03 and 1.17, respectively. SWEWOA ranked first in the comprehensive ranking of the two evaluation methods, and BMWOA ranked second, with WSRT and FT average ranks of 2.60 and 3.09, respectively. Figure 8 is the convergence graph of the seven competitors on the partial functions. In Figure 8, the red line is the lowest among all the methods, which illustrates that its convergence accuracy of SWEWOA is superior to the above six excellent WOA-improved algorithms.

#### The comparison of SWEWOA and advanced algorithms for IEEE CEC2017

The dominance of SWEWOA is confirmed by comparison with the popular primitive intelligent algorithms and excellent WOA variants. However, comparison with these algorithms alone is not enough to confirm the validity of SWEWOA. Therefore, in this section, the capacity differences between SWEWOA and other advanced variants of algorithms are compared to demonstrate the superiority of SWEWOA. These advanced variants of other algorithms include ASCA_PSO, SCADE, MSFOA, GWOSCA, HGWO, and CMFO.

**Figure 9:**
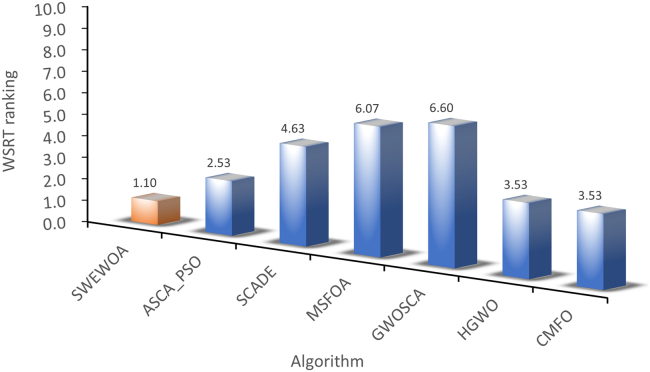
WSRT ranking of the other variant algorithms for IEEE CEC2017

**Figure 10:**
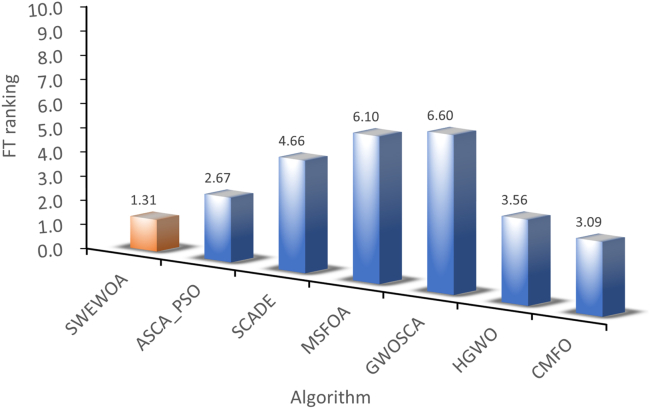
FT ranking of the other variant algorithms for IEEE CEC2017

**Figure 11:**
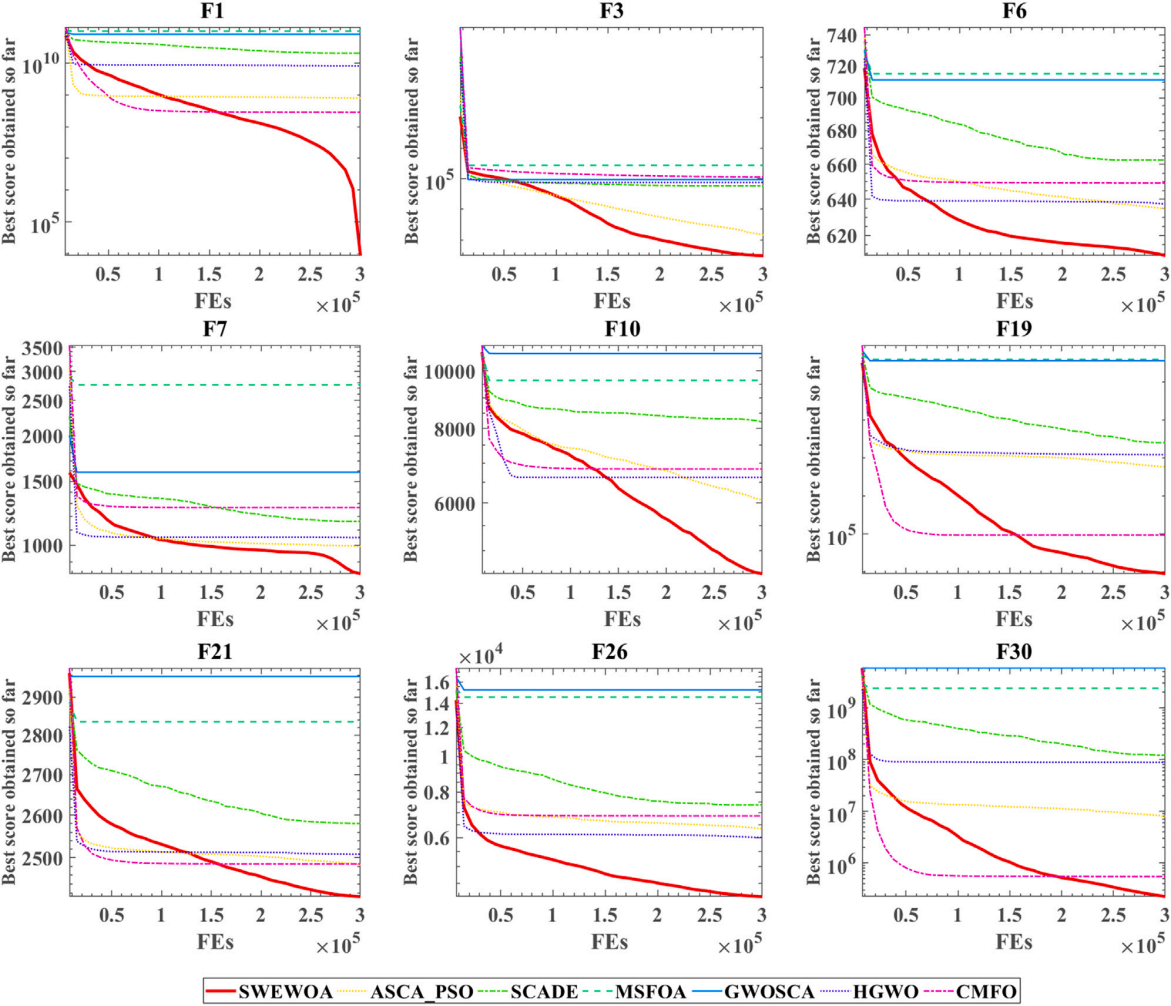
Convergence curve of the other variant algorithms

The mean and standard deviation of SWEWOA compared with the other 6 competitors are displayed in Table C7. Table C7 expresses that the optimal mean number of SWEWOA is 27, and the optimal standard deviation number is 12, ranking first. Therefore, the overall effect of SWEWOA is stronger than the improved algorithms of the other six well-known techniques. Table C8 shows the significance analysis of the comparison results between SWEWOA and the other six algorithms. In the table, there are only 4 p values greater than or equal to 0.05. This shows that in most functions, these six algorithms are significantly different from SWEWOA. Meanwhile, in terms of the number of "+", SWEWOA is far more than other algorithms. Although SWEWOA is weaker in F27 than MSFOA and weaker in F13 than CMFO (this may be the reason that the properties of MSFOA and CMFO algorithms apply to F27 and F13, respectively), SWEWOA outperforms MSFOA in 29 other functions. The number of those better than CMFO is 27. This shows that SWEWOA has the strongest optimization performance in terms of overall optimization performance. Figures 9 and 10 show the comprehensive ranking of the two evaluation methods of the algorithm in 7. The average rank of WSRT and FT of SWEWOA is 1.10 and 1.31, respectively. SWEWOA ranked first in the combined rankings of both methods. ASCA_PSO ranked second, and the average rank of the two methods was 2.53 and 2.67, respectively. The convergence curves of the seven competitors in partial functions are drawn in the Figure 11. From the convergent curve it is not hard to find that in functions F1, F3, F6, F7, and F19, the initial solution of SWEWOA is below the other six algorithms, because SWEWOA uses sine mapping initialization strategy instead of the original random generation strategy. The initial swarm of the presented SWEWOA is of high quality. It is worth noting that the red line is at the bottom of all curves, which indicates that SWEWOA can explore the location of optimal solutions with better quality, and its accuracy of convergence is higher than the other six competitors.

#### The comparison of SWEWOA and advanced algorithms for IEEE CEC2022

The SWEWOA presented in this paper demonstrated superior optimization performance in the CEC2017 test sets. To further confirm the capability of SWEWOA, this section presents its performance in the CEC2022 test sets. The specific description of these 12 test functions of IEEE CEC2022 are described in Table B3. In addition, this section selects new algorithms proposed in recent years with strong optimization performance as new comparison algorithms. Qiao et al. proposed to introduce individual disturbance and neighborhood mutation (WDNMWOA)^61^ to avoid WOA from falling into local optima. The BWOA^82^ with Lévy flight and chaotic local search is prominent in constrained engineering design problems. In FSTPSO,^83^ the application of fuzzy logic effectively improves the convergence speed of the algorithm. Jia et al. proposed a satellite image segmentation technique based on dynamic Harris hawks optimization with a mutation mechanism (DHHOM).^84^ GWO^85^ and BA^86^ are inspired by the behavior of wolf and bat groups in nature, respectively. The detailed parameter settings of the above competitors are presented in Table 1.

Table C9 illustrates the mean value and standard deviation of the above competitors in the CEC2022 test set. From the results in the table, the number of functions for SWEWOA to obtain the minimum mean is 10. This shows that SWEWOA can obtain solutions with lower values than other comparison algorithms. The difference analysis between SWEWOA and other comparison algorithms is given in Table C10 of the supplemental information. The results indicate that SWEWOA is significantly superior to other comparison algorithms in most functions. First of all, SWEWOA clearly wins out of 11 functions compared with the advanced algorithms DHHOM and BA. Second, SWEWOA completely outperformed the FSTPSO throughout the test sets. In addition, the proposed SWEWOA outperforms WDNMWOA on 8 functions and performs approximately equally on the other 4 functions. Compared with another WOA variant named BWOA, SWEWOA performs significantly better than BWOA on 11 test functions. This shows that the three strategies introduced by SWEWOA are effective and perform better than other newly developed variants of WOA. Tables 8 and 9 present the WSRT ranking and FT ranking of the above algorithms in the CEC2022 test sets. SWEWOA ranked first overall with an average of 1.25 and 1.84, respectively.

**Table 8:** WRST results of the competitors for IEEE CEC2022

Methods	SWEWOA	WDNMWOA	BWOA	FSTPSO	DHHOM	GWO	BA
Mean_rank	**1.25**	3.33	4.58	6.25	4.17	3.42	4.92
Rank	**1**	2	5	7	4	3	6

**Table 9:** FT results of the competitors for IEEE CEC2022

Methods	SWEWOA	WDNMWOA	BWOA	FSTPSO	DHHOM	GWO	BA
Mean_rank	**1.84**	3.59	4.43	5.99	4.21	3.11	4.82
Rank	**1**	3	5	7	4	2	6

**Figure 12:**
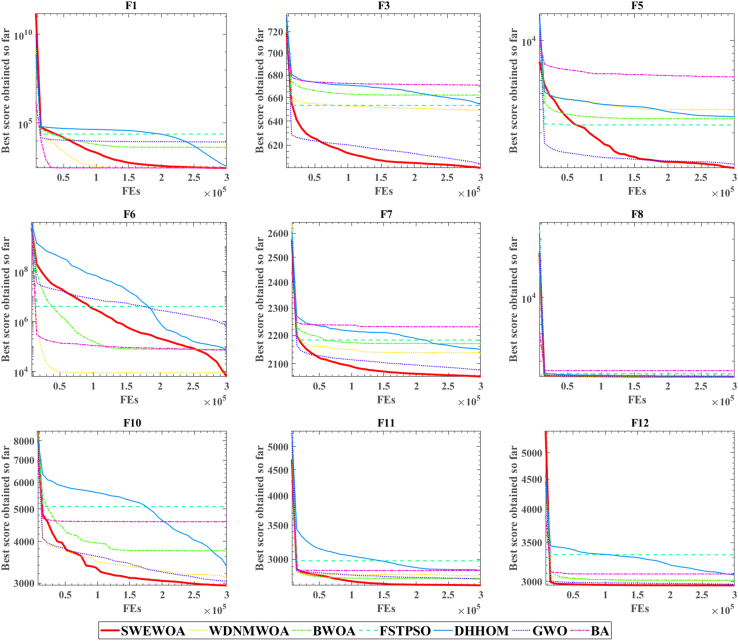
Convergence curve of the algorithms for IEEE CEC2022

Figure 12 shows the convergence curve of the comparison algorithm. The red line indicates the SWEWOA proposed in this paper. From the convergence curves of functions F3, F5, F8, and F11, the starting position of the red line is always lower than that of other algorithms. This is why sine mapping initialization is introduced to improve the initial population quality. In the convergence curves of F3, F5, F6, and F10 functions, it is not difficult to find that other algorithms have already fallen into local optimum, whereas the red line can continue exploring other better-quality solutions.

In a nutshell, the performance of SWEWOA in the latest CEC2022 test tests is still superior.

### Feature selection experiment

#### Competitive algorithms and public datasets

In the part, a new machine learning model on the basis of the binary version of SWEWOA (BSWEWOA) and KELM is proposed, named BSWEWOA-KELM. To confirm the superiority of the suggested method, the proposed BSWEWOA-KELM was compared with other six excellent swarm intelligence algorithms-based KELM models on 13 public datasets. The specific content of the public datasets and the specific parameter settings of the comparison algorithm are given in Table 10 below and Table B2 in the supplemental information, respectively.

**Table 10:** Characteristics of public datasets

Dataset	Samples	Features
clean1	476	166
Breastcancer	699	9
German	1000	24
wdbc	569	30
Breast	569	30
Vote	101	16
heartandlung	139	23
JPNdata	152	10
heart	270	13
Parkinson	195	22
Sonar	208	60
Wielaw	240	30
thyroid_2class	187	8

#### Evaluation criteria

The results are assessed utilizing a 10-fold cross-validation analysis to ensure that the test outcomes were objective and effective. Fitness, Average feature number, Accuracy, MCC, F-measure, and other indicators were used to verify the performance and classification effectiveness. Calculation methods for evaluation indicators other than fitness and average number of features are given in Tables 11 and 12.

**Table 11:** The confusion matrix

	(N)Negative	(P)Positive
F(False)	FN	FP
T(True)	TN	TP

**Table 12:** Evaluation criteria

Name	Formula	Remark
Accuracy	$Accuracy=\frac{TP+TN}{TP+FP+FN+TN}$	A higher accuracy rate represents a larger percentage of the sample that is correctly predicted.
Specificity	$Specificity=\frac{TN}{TN+FP}$	The higher the specificity, the lower the classification error.
Precision	$Precision=\frac{TP}{TP+FP}$	A higher precision indicates a more accurate prediction of positive cases.
MCC	$MCC=\frac{TP\times TN-FP\times FN}{\sqrt{\left(TP+FP\right)\times \left(TP+FN\right)\times \left(TN+FP\right)\times \left(TN+FN\right)}}$	A closer MCC to 1 indicates a more perfect prediction of the subject.
F-measure	$F-measure=\frac{TP}{TP+\frac{FN+FP}{2}}$	The F-value represents whether the predicted result is in line with expectations, and the higher the value, the more in line with expectations.

#### Feature selection results of competitive algorithms on public datasets

Compared with BGWO, BGSA, BPSO, BBA, BSSA, and BWOA, the average fitness value of BSWEWOA under 50 iterations is given in Table 13, where the optimal solution is highlighted. The results show that the competitors are significantly weaker than the proposed BSWEWOA algorithm in any dataset. This is because the sine initialization strategy makes the optimal solution can be quickly searched when the whale is initialized, which is conducive to a more effective optimization search of whales in the following. Moreover, the wormhole strategy improves the capacity of BSWEWOA to keep from dropping into the local optimum and improves the convergence accuracy of BSWEWOA. Therefore, in terms of fitness, the excellent performance of BSWEWOA demonstrates that it has the best search ability and feature solving ability.

**Table 13:** The results of SWEWOA and other competitors in fitness

Dataset	Items	BSWEWOA	BGWO	BGSA	BPSO	BBA	BSSA	BWOA
Breast	Avg	**1.761900E-02**	2.097200E-02	2.430700E-02	2.113900E-02	3.336300E-02	3.150200E-02	2.286300E-02
	Stdv	1.265100E-02	1.370400E-02	1.295400E-02	**1.213700E-02**	1.858100E-02	1.412600E-02	1.409100E-02
clean1	Avg	**6.858700E-03**	2.822700E-02	4.198100E-02	3.258500E-02	7.350300E-02	6.930300E-02	3.452300E-02
	Stdv	**5.884800E-03**	1.392000E-02	2.623700E-02	1.588700E-02	2.244500E-02	2.845100E-02	1.971400E-02
heartandlung	Avg	**1.200300E-02**	3.192500E-02	3.366500E-02	2.074500E-02	3.996900E-02	3.253100E-02	2.183200E-02
	Stdv	**2.132900E-02**	4.763400E-02	3.613900E-02	3.032100E-02	3.662600E-02	3.073400E-02	2.875500E-02
Breastcancer	Avg	**2.667800E-02**	3.077000E-02	2.939400E-02	2.746100E-02	3.020000E-02	3.192000E-02	2.996800E-02
	Stdv	1.263500E-02	1.242600E-02	1.562500E-02	1.561500E-02	1.438300E-02	1.396200E-02	**1.151700E-02**
German	Avg	**1.808700E-01**	1.842300E-01	1.881000E-01	1.848600E-01	2.044800E-01	1.926800E-01	1.837000E-01
	Stdv	**1.837200E-02**	2.142000E-02	2.232100E-02	2.947700E-02	2.280700E-02	2.365200E-02	1.855700E-02
JPNdata	Avg	**7.355100E-02**	1.181600E-01	8.592600E-02	1.105700E-01	1.008000E-01	1.122600E-01	9.819600E-02
	Stdv	6.430900E-02	7.620100E-02	5.006400E-02	6.354900E-02	4.869000E-02	6.310500E-02	**4.071200E-02**
Vote	Avg	**2.382300E-02**	3.907900E-02	2.989100E-02	4.287100E-02	3.972900E-02	4.283800E-02	3.030600E-02
	Stdv	1.796100E-02	2.312100E-02	2.617200E-02	3.749900E-02	2.851700E-02	3.904700E-02	**1.584300E-02**
wdbc	Avg	**2.119600E-02**	2.350000E-02	2.883500E-02	2.775700E-02	3.663900E-02	3.406200E-02	2.672700E-02
	Stdv	**1.503300E-02**	1.544500E-02	1.279100E-02	1.717700E-02	1.775700E-02	1.115400E-02	1.352300E-02
heart	Avg	**6.816200E-02**	6.931600E-02	8.333300E-02	7.673800E-02	8.327600E-02	9.861800E-02	8.146700E-02
	Stdv	**2.864100E-02**	4.096900E-02	3.020800E-02	4.670000E-02	3.499000E-02	6.228300E-02	4.790100E-02
Parkinson	Avg	**3.968200E-02**	5.516400E-02	6.097700E-02	6.425500E-02	6.804500E-02	5.102300E-02	5.107300E-02
	Stdv	3.389300E-02	4.440000E-02	**2.961500E-02**	4.598600E-02	5.435500E-02	4.890100E-02	4.307500E-02
Sonar	Avg	**1.471400E-02**	2.410700E-02	3.995200E-02	4.317900E-02	7.507700E-02	5.101200E-02	2.840200E-02
	Stdv	**1.997700E-02**	1.798400E-02	3.335500E-02	4.051200E-02	4.829800E-02	2.424700E-02	3.095200E-02
thyroid_2class	Avg	**1.092400E-01**	1.388300E-01	1.275600E-01	1.275700E-01	1.383500E-01	1.310300E-01	1.400800E-01
	Stdv	6.007100E-02	4.492800E-02	5.153100E-02	**4.042800E-02**	6.339300E-02	5.701300E-02	5.547700E-02
Wielaw	Avg	**1.124400E-01**	1.363700E-01	1.303100E-01	1.355400E-01	1.580800E-01	1.673100E-01	1.389100E-01
	Stdv	6.758300E-02	6.794900E-02	7.733300E-02	**4.233900E-02**	5.292400E-02	5.443500E-02	6.218300E-02
Mean_rank		**1.00**	4.15	4.00	4.00	4.77	5.54	3.62
Rank		**1**	5	3	3	6	7	2

Table 14 indicates the comparison outcomes of the accuracy indexes of BSWEWOA and the other six algorithms. As you can see from the table, the average ranking of BSWEWOA is 1.00, which means that BSWEWOA is number one in every dataset. Therefore, in terms of accuracy, BSWEWOA performs best on public datasets. The specificity indexes of BSWEWOA and other algorithms are provided in Table C11. In plain sight from Table C11, BSWEWOA stands first in the seven competitors with a mean ranking of 1.38. This illustrates that BSWEWOA performs best on most public datasets. Table 15 gives the precision comparison results of seven competitors. The average ranking of BSWEWOA algorithm is 1.38, ranking first overall, and the average ranking of BWOA is 3.46, ranking third in the overall ranking, indicating the superiority of the proposed improved strategy for WOA in strengthening the classification accuracy. The MCC of seven competitors are presented in Table C12. In the table, BSWEWOA is the best in most of the datasets, and BSWEWOA ranks first overall with an average ranking of 1.08. Table C13 is the F-measure evaluation index of BSWEWOA et al. It is not difficult to see from Table C13 that the average ranking of BSWEWOA is 1.08, ranking first in the comprehensive ranking, and the mean value of F-measure is close to 1, representing that the prediction result of BSWEWOA is very acceptable. Table 16 shows the mean value of the number of features selected by BSWEWOA and other algorithms in the dataset. BSWEWOA can simplify the dimension of the dataset to the maximum extent in most datasets. BSWEWOA ranked first in eight of the datasets. Importantly, in Breast, clean1, wdbc, and Sonar, the ability of BSWEWOA to simplify the dataset far exceeds that of the other six high-performance algorithms. Although the ranking of the BSWEWOA algorithm in heartandlung, Vote, thyroid_2class, and Wielaw is not as good as that of the famous algorithm BGWO, there is no significant difference in the mean of the number of features selected by BSWEWOA algorithm. The same situation applies to BreastCancer: BPSO in Parkinson dataset and BWOA in heart dataset. Although BSWEWOA is inferior to BGWO, BPSO, and BWOA in reducing the number of features in some datasets, BSWEWOA can maintain very close results with BGWO, BPSO, and BWOA in the inferior datasets, and its performance in the dominant datasets is far better than any other six algorithms. Therefore, it can be shown that BSWEWOA performs better than other comparison algorithms. Importantly, the main goal of wrap-based methods is to choose the subset of features that best perform the model. Therefore, if all the tables showing the experimental results in this section are considered together, it can be found that although BSWEWOA is not dominant in the number of features selected in some datasets, BSWEWOA is far superior to other algorithms in the most critical aspects such as fitness, accuracy, and precision. This suggests that the BSWEWOA algorithm has the highest accuracy in searching the optimal feature. BSWEWOA can use its optimal search optimization ability, the highest accuracy, and the best feature acquisition ability to determine the critical feature subset that can strengthen the model performance the most.

**Table 14:** The results of SWEWOA and other competitors in accuracy

Dataset	Items	BSWEWOA	BGWO	BGSA	BPSO	BBA	BSSA	BWOA
Breast	Avg	**9.893500E-01**	9.859900E-01	9.859900E-01	9.877500E-01	9.876900E-01	9.859600E-01	9.876900E-01
	Stdv	**1.244700E-02**	1.374700E-02	1.383700E-02	1.435100E-02	1.859500E-02	1.375600E-02	1.445600E-02
clean1	Avg	**9.979200E-01**	9.894100E-01	9.768600E-01	9.853300E-01	9.537600E-01	9.558100E-01	9.811600E-01
	Stdv	**6.588100E-03**	1.501100E-02	2.709600E-02	1.719100E-02	2.582700E-02	3.050200E-02	2.072800E-02
heartandlung	Avg	**9.928600E-01**	9.714300E-01	9.714300E-01	9.857100E-01	9.714300E-01	9.785700E-01	9.857100E-01
	Stdv	**2.258800E-02**	4.994300E-02	3.688600E-02	3.011700E-02	3.688600E-02	3.450300E-02	3.011700E-02
Breastcancer	Avg	**9.871200E-01**	9.828200E-01	9.842600E-01	9.857100E-01	9.857500E-01	9.827700E-01	9.842400E-01
	Stdv	1.251100E-02	1.131600E-02	1.718100E-02	1.649800E-02	1.501700E-02	1.325900E-02	**1.053500E-02**
German	Avg	**8.300000E-01**	8.280000E-01	8.270000E-01	8.280000E-01	8.170000E-01	8.270000E-01	8.290000E-01
	Stdv	**1.885600E-02**	2.440400E-02	2.406000E-02	2.859700E-02	3.233500E-02	2.584100E-02	2.024800E-02
JPNdata	Avg	**9.336300E-01**	8.872000E-01	9.211300E-01	8.957100E-01	9.086300E-01	8.944600E-01	9.082100E-01
	Stdv	6.882100E-02	7.850300E-02	5.214500E-02	7.007000E-02	5.189600E-02	7.180900E-02	**4.515900E-02**
Vote	Avg	**9.864400E-01**	9.667600E-01	9.767600E-01	9.631000E-01	9.733100E-01	9.660900E-01	9.766500E-01
	Stdv	1.751400E-02	2.188200E-02	2.744700E-02	4.053800E-02	3.067700E-02	3.938400E-02	**1.612900E-02**
wdbc	Avg	**9.841800E-01**	9.824600E-01	9.807000E-01	9.806100E-01	9.807300E-01	9.806400E-01	9.841500E-01
	Stdv	1.745000E-02	1.654100E-02	**1.285600E-02**	1.757800E-02	1.536600E-02	1.303200E-02	1.305100E-02
heart	Avg	**9.444400E-01**	**9.444400E-01**	9.333300E-01	9.370400E-01	9.370400E-01	9.148100E-01	9.296300E-01
	Stdv	**3.147500E-02**	4.701100E-02	3.403500E-02	4.953700E-02	3.513600E-02	6.773300E-02	5.075300E-02
Parkinson	Avg	**9.642100E-01**	9.483900E-01	9.439500E-01	9.378700E-01	9.436800E-01	9.592100E-01	9.536500E-01
	Stdv	3.502600E-02	4.781100E-02	**2.925300E-02**	4.740000E-02	5.599200E-02	5.240100E-02	4.466900E-02
Sonar	Avg	**9.904800E-01**	9.902400E-01	9.761900E-01	9.709500E-01	9.478100E-01	9.709500E-01	9.854500E-01
	Stdv	**2.007800E-02**	2.058800E-02	3.367200E-02	4.105300E-02	5.201200E-02	2.501700E-02	3.325100E-02
thyroid_2class	Avg	**9.040900E-01**	8.657000E-01	8.815200E-01	8.821600E-01	8.721300E-01	8.765500E-01	8.657000E-01
	Stdv	6.495600E-02	4.756900E-02	5.678100E-02	**4.393600E-02**	6.706400E-02	6.330300E-02	6.028800E-02
Wielaw	Avg	**8.874300E-01**	8.620700E-01	8.744100E-01	8.669700E-01	8.541200E-01	8.424900E-01	8.664100E-01
	Stdv	6.824200E-02	7.122500E-02	8.162300E-02	**4.641500E-02**	5.645300E-02	5.950600E-02	6.509700E-02
Mean_rank		**1.00**	4.15	4.00	4.00	4.77	5.54	3.62
Rank		**1**	5	3	3	6	7	2

**Table 15:** The results of SWEWOA and other competitors in precision

Dataset	Items	BSWEWOA	BGWO	BGSA	BPSO	BBA	BSSA	BWOA
Breast	Avg	**9.836300E-01**	9.785900E-01	9.785200E-01	9.812900E-01	9.815600E-01	9.785100E-01	9.812900E-01
	Stdv	**1.899500E-02**	2.086600E-02	2.108200E-02	2.178700E-02	2.746800E-02	2.088900E-02	2.178700E-02
clean1	Avg	**1.000000E+00**	9.927200E-01	9.961500E-01	9.888900E-01	9.697800E-01	9.810100E-01	9.888800E-01
	Stdv	**0.000000E+00**	1.534000E-02	1.216300E-02	1.789100E-02	2.366200E-02	2.619500E-02	1.791900E-02
heartandlung	Avg	**9.875000E-01**	9.607100E-01	9.750000E-01	**9.875000E-01**	9.750000E-01	9.625000E-01	9.750000E-01
	Stdv	**3.952800E-02**	6.344200E-02	5.270500E-02	**3.952800E-02**	5.270500E-02	6.038100E-02	5.270500E-02
Breastcancer	Avg	9.936600E-01	9.914400E-01	**9.955100E-01**	9.913000E-01	9.913500E-01	9.914300E-01	9.915300E-01
	Stdv	1.416800E-02	1.104800E-02	**9.476900E-03**	1.123100E-02	1.117200E-02	1.480000E-02	1.469800E-02
German	Avg	**8.328400E-01**	8.310200E-01	8.309300E-01	8.305900E-01	8.244400E-01	8.281300E-01	8.307500E-01
	Stdv	**1.871200E-02**	3.355200E-02	2.521300E-02	2.399400E-02	2.925500E-02	2.531500E-02	1.936000E-02
JPNdata	Avg	**9.336300E-01**	8.872000E-01	9.211300E-01	8.957100E-01	9.086300E-01	8.944600E-01	9.082100E-01
	Stdv	6.882100E-02	7.850300E-02	5.214500E-02	7.007000E-02	5.189600E-02	7.180900E-02	**4.515900E-02**
Vote	Avg	**9.509900E-01**	8.482800E-01	9.252800E-01	9.166700E-01	9.083300E-01	8.942100E-01	8.896000E-01
	Stdv	**6.386300E-02**	1.064700E-01	8.464500E-02	1.165600E-01	8.861800E-02	9.118600E-02	8.308600E-02
wdbc	Avg	**1.000000E+00**	**1.000000E+00**	**1.000000E+00**	**1.000000E+00**	**1.000000E+00**	**1.000000E+00**	**1.000000E+00**
	Stdv	**0.000000E+00**	**0.000000E+00**	**0.000000E+00**	**0.000000E+00**	**0.000000E+00**	**0.000000E+00**	**0.000000E+00**
heart	Avg	**9.443900E-01**	9.327600E-01	9.254800E-01	9.287600E-01	9.259000E-01	9.095800E-01	9.151100E-01
	Stdv	5.288800E-02	5.714400E-02	**4.656200E-02**	7.060400E-02	4.665800E-02	7.948400E-02	6.117800E-02
Parkinson	Avg	**9.611900E-01**	9.387500E-01	9.494300E-01	9.436500E-01	9.452200E-01	9.574400E-01	9.545800E-01
	Stdv	**3.351600E-02**	5.425700E-02	3.863600E-02	5.388800E-02	6.039700E-02	5.424800E-02	4.305300E-02
Sonar	Avg	**9.909100E-01**	9.900000E-01	9.809100E-01	9.718200E-01	9.360600E-01	9.718200E-01	**9.909100E-01**
	Stdv	**2.874800E-02**	3.162300E-02	4.030400E-02	6.246400E-02	7.319500E-02	4.544400E-02	**2.874800E-02**
thyroid_2class	Avg	9.492100E-01	9.268900E-01	**9.505100E-01**	8.653800E-01	8.605600E-01	9.388900E-01	8.989300E-01
	Stdv	**6.642300E-02**	1.045000E-01	9.102700E-02	9.978400E-02	1.339600E-01	1.080100E-01	9.085200E-02
Wielaw	Avg	**8.696100E-01**	8.554800E-01	8.763600E-01	8.539300E-01	8.753700E-01	8.205300E-01	**9.003300E-01**
	Stdv	9.071400E-02	1.186900E-01	1.118200E-01	8.504900E-02	**7.338400E-02**	9.303300E-02	1.108200E-01
Mean_rank		**1.00**	4.15	4.00	4.00	4.77	5.54	3.62
Rank		**1**	5	3	3	6	7	2

**Table 16:** The results of SWEWOA and other competitors in average feature number

Dataset	Items	BSWEWOA	BGWO	BGSA	BPSO	BBA	BSSA	BWOA
Breast	Avg	**4.50**	**4.50**	6.60	5.70	13.00	14.50	13.10
clean1	Avg	**16.20**	60.30	66.40	61.90	98.20	90.70	55.20
heartandlung	Avg	2.40	**2.20**	3.00	3.30	5.90	5.60	3.80
Breastcancer	Avg	2.60	2.60	2.60	**2.50**	2.80	2.80	2.70
German	Avg	**9.30**	10.00	11.40	10.30	14.70	13.60	10.20
JPNdata	Avg	**2.10**	2.20	2.20	2.30	2.80	2.40	2.20
Vote	Avg	3.50	**2.40**	2.50	2.50	4.60	3.40	2.60
wdbc	Avg	**3.70**	4.10	6.30	5.60	11.00	9.40	7.00
heart	Avg	4.00	4.30	5.20	4.40	6.10	4.60	**3.80**
Parkinson	Avg	2.50	2.70	3.40	**2.30**	6.40	5.40	3.10
Sonar	Avg	**6.80**	17.80	20.80	18.70	30.60	28.10	17.50
thyroid_2class	Avg	2.90	**1.80**	2.40	2.50	2.70	2.20	2.00
Wielaw	Avg	3.30	**3.20**	6.60	5.50	11.70	10.60	7.20
Mean_rank		2.23	**1.85**	3.92	3.31	6.69	5.69	3.54
Rank		**2**	**1**	5	3	7	6	4

In summary, according to the performance of BSWEWOA in the above 13 datasets, it is not difficult to see that BSWEWOA has the best performance among all algorithms.

#### Testing of BSWEWOA-KELM on high-dimensional datasets

In Section 4.3.3, the selected datasets are low-dimensional datasets. Next, in this section, we will select several high dimensional datasets to confirm the validity of the proposed model. In this paper, another 2 excellent algorithms are selected, which are the standard moth-flame optimization (BMFO) and the gray wolf optimizer with chaotic diffusion-limited aggregation (BSCGWO).^87^ The specific parameter settings of the BMFO and BSCGWO are provided in Table 2. The specific content of the high-dimensional datasets is given in Table 17.

**Table 17:** Details of high-dimensional datasets

Dataset	Samples	Features
Colon	62	2000
Lung_Cancer	203	12600
Semeion	1593	255

Table 18 shows the average fitness value of the competitors. From the table, BSWEWOA achieves better quality fitness values in all three datasets, and the quality of the fitness values achieved in the Colon dataset is second only to BMFO. This means that BSWEWOA also maintains excellent optimization capability in dealing with high-dimensional datasets. Table 19 describes the prediction accuracy of the algorithms. From the table, in the three datasets, the prediction accuracy of the BSWEWOA-KELM is stronger than the other comparison algorithms. This indicates that BSWEWOA-KELM correctly predicted a larger proportion of samples than other algorithms. In addition, in the Colon dataset, the accuracy of BSWEWOA-KELM reaches 88.3%, the accuracy of BMFO ranked second is only 76.7%, and the prediction accuracy of the original WOA without any improvement is only 45%, which suggests that the improvement strategy of this paper greatly enhances the performance of WOA. Tables 20 and 21, respectively, show the precision and feature number of the algorithm in high-dimensional datasets. In Table 20, the precision of BSWEWOA ranks first overall, so it can be concluded that BSWEWOA-KELM has a high level of prediction for positive samples. Combining Tables 19 and 20, the results demonstrate that the classification accuracy and precision of the original BWOA ranked 9th and 8th, respectively, whereas the BSWEWOA ranked first overall. This illustrates that the introduction of the three strategies greatly strengthens the capability of WOA. In Table 21, the average number of features obtained by the proposed model in three different high-dimensional datasets is 167.2, 1411.8, 9.0, and 26.2, respectively. Combining Tables 18, 19, 20, and 21, it can be found that BSWEWOA-KELM can greatly simplify the dimensions of the dataset while having excellent prediction performance.

**Table 18:** The fitness of the algorithms in high-dimensional datasets

	Colon		Lung_Cancer		Semeion	
	Avg	Stdv	Avg	Stdv	Avg	Stdv
BSWEWOA	5.536000E-01	1.152300E-01	**8.761200E-01**	2.229100E-02	**4.943400E-03**	2.169200E-03
BSCGWO	7.611000E-01	1.361400E-01	8.951600E-01	2.227700E-02	2.934600E-02	4.184500E-03
BMFO	**5.505600E-01**	1.309800E-01	8.767000E-01	**1.931300E-02**	**4.914500E-03**	1.878100E-03
BGWO	7.221100E-01	1.336400E-01	8.930400E-01	2.227600E-02	1.209400E-02	5.930000E-04
BGSA	7.722600E-01	1.174300E-01	8.944500E-01	2.141100E-02	2.000000E-02	1.067300E-03
BPSO	7.543700E-01	1.490900E-01	8.945600E-01	2.063600E-02	1.756600E-02	**8.365000E-04**
BBA	7.232800E-01	1.762800E-01	8.767500E-01	2.034000E-02	2.941900E-02	4.091200E-03
BSSA	7.418100E-01	**1.018000E-01**	8.823200E-01	1.937800E-02	2.320800E-02	2.560900E-03
BWOA	7.669300E-01	1.734200E-01	8.876400E-01	2.136100E-02	1.720100E-02	1.703000E-03

**Table 19:** The accuracy of the algorithms in high-dimensional datasets

	Colon		Lung_Cancer		Semeion	
	Avg	Stdv	Avg	Stdv	Avg	Stdv
BSWEWOA	**8.833300E-01**	**2.490700E-01**	**8.366500E-02**	2.354000E-02	**1.000000E+00**	**0.000000E+00**
BSCGWO	5.333300E-01	3.751500E-01	8.362200E-02	2.341900E-02	9.987400E-01	2.651800E-03
BMFO	7.666700E-01	2.509200E-01	8.279200E-02	**2.026900E-02**	9.993800E-01	1.976400E-03
BGWO	5.666700E-01	2.854500E-01	8.364700E-02	2.340800E-02	**1.000000E+00**	**0.000000E+00**
BGSA	4.500000E-01	2.945000E-01	8.335800E-02	2.252200E-02	**1.000000E+00**	**0.000000E+00**
BPSO	4.833300E-01	3.374700E-01	8.314500E-02	2.175700E-02	**1.000000E+00**	**0.000000E+00**
BBA	5.166700E-01	4.116400E-01	8.359600E-02	2.324900E-02	9.981100E-01	3.044600E-03
BSSA	5.166700E-01	2.986600E-01	8.279200E-02	**2.026900E-02**	**1.000000E+00**	**0.000000E+00**
BWOA	4.500000E-01	3.689300E-01	8.335800E-02	2.252200E-02	9.993700E-01	1.988900E-03

**Table 20:** The precision of the algorithms in high-dimensional datasets

	Colon		Lung_Cancer		Semeion	
	Avg	Stdv	Avg	Stdv	Avg	Stdv
BSWEWOA	**1.000000E+00**	**0.000000E+00**	**8.366500E-02**	2.354000E-02	**1.000000E+00**	**0.000000E+00**
BSCGWO	8.000000E-01	4.216400E-01	8.362200E-02	2.341900E-02	9.986100E-01	2.928000E-03
BMFO	**1.000000E+00**	**0.000000E+00**	8.279200E-02	**2.026900E-02**	9.993100E-01	2.180900E-03
BGWO	9.000000E-01	3.162300E-01	8.364700E-02	2.340800E-02	**1.000000E+00**	**0.000000E+00**
BGSA	8.000000E-01	4.216400E-01	8.335800E-02	2.252200E-02	**1.000000E+00**	**0.000000E+00**
BPSO	8.000000E-01	4.216400E-01	8.314500E-02	2.175700E-02	**1.000000E+00**	**0.000000E+00**
BBA	5.166700E-01	4.116400E-01	8.359600E-02	2.324900E-02	9.981100E-01	3.044600E-03
BSSA	7.000000E-01	4.830500E-01	8.279200E-02	**2.026900E-02**	**1.000000E+00**	**0.000000E+00**
BWOA	7.000000E-01	4.830500E-01	8.335800E-02	2.252200E-02	9.993100E-01	2.196000E-03

**Table 21:** The average feature number of the algorithms in high-dimensional datasets

Dataset	Items	BSWEWOA	BSCGWO	BMFO	BGWO	BGSA	BPSO	BBA	BSSA	BWOA
Colon	Avg	**167.2**	1039.3	172.6	764.3	870.4	851.6	286.6	665.7	657.3
Lung_Cancer	Avg	1411.8	6200.1	**1348.5**	5672	5956.5	5933.2	1555.1	2765.9	4241.2
Semeion	Avg	26.2	149.2	**22.9**	64.1	106	93.1	146.4	123	88
Rank		**2**	9	**1**	5	8	7	4	6	3

In conclusion, BSWEWOA-KELM also has an excellent performance in high-dimensional datasets.

### Limitations of the study

This study introduces enhancement strategies to improve the performance of WOA. However, there are still several limitations in this study. First, the impact of different strategies on WOA is not evaluated in the feature selection experiments. Initially, the impact of three different strategies on WOA is tested on the CEC2017 test set in the global optimization task. A more in-depth evaluation of the impact of the three mechanisms could also be carried out. Secondly, in the feature selection task, the maximum number of features for our selected dataset is 12,600. In this range, BSWEWOA achieves a satisfactory performance. And when the number of features exceeds this value, the performance of BSWEWOA is waiting to be evaluated. We recommend that the performance of BSWEWOA in higher dimensional datasets be further evaluated. Finally, it is clear from the experiments that the algorithm takes a long time to execute. To address this issue, incorporating parallel computing into the algorithm could be an option.

### Conclusions and future works

In the study, sine initialization strategy, escape energy, and wormhole search mechanism are combined into WOA to strengthen the global optimization capability of the algorithm. To demonstrate the optimization ability of SWEWOA, the article conducts a policy combination experiment, historical searching experiment, experimental analysis of stability in different dimensions, meta-heuristic algorithms comparison experiment, WOA variant algorithms, and other advanced algorithms comparison experiment. Through policy combination experiment and historical searching experiment, it is proved that when three strategies are all introduced into WOA, the optimization ability is most improved. This is because the sine initialization policy can generate whales with higher initial quality, allowing the whale to find a more suitable search direction. Moreover, introducing escape energy will enable whales to behave more rationally and cost-effectively. Meanwhile, the wormhole search mechanism helps to prevent WOA from dropping into the trap of local optimality. The stability experiment results indicate that SWEWOA has superior optimization capacity in low and high latitudes. In addition, the effectiveness of SWEWOA is further confirmed by comparing it with several famous original methods and high-performance improved algorithms. The comparison results suggest that this method has excellent optimization ability and can obtain better solutions. SWEWOA shows greater global optimization capability significantly better than other competitors. Finally, SWEWOA succeeds in the classification accuracy of feature selection. Furthermore, a new method based on a binary version of SWEWOA and KELM (BSWEWOA-KELM) is proposed, and 13 public datasets confirm the capability of the model. The outcomes show that BSWEWOA-KELM has a marked predominance over other competitors constructed by the original WOA, PSO, and GWO algorithms in some key performance indicators. BSWEWOA-KELM has good results in search ability, solution quality, and selection of optimal features. In addition, the strong performance on high-dimensional datasets proves that the proposed model performs well not only on low-dimensional datasets but also on high-dimensional datasets. Therefore, it can be concluded that the proposed SWEWOA has excellent applications in feature selection, and the BSWEWOA-KELM may be regarded as a valuable decision support tool.

In the future, there are still some rooms that deserve further investigation. For instance, on the premise that SWEWOA has high convergence accuracy, SWEWOA is made to have a faster convergence speed to strengthen the global optimization ability further. In addition, the proposed method can be extended to engineering design optimization and image segmentation.

## STAR★Methods

### Key resources table

**Table:** 

REAGENT or RESOURCE	SOURCE	IDENTIFIER
**Software and algorithms**		

Whale Optimization Algorithm (WOA)	Seyedali Mirjalili	http://www.alimirjalili.com/WOA.html

### Resource availability

#### Lead contact

Further requests for information should be directed and will be handled by the lead contact, Huiling Chen, email: chenhuiling.jlu@gmail.com.

#### Materials availability

This study did not generate new materials.

### Method details

#### Overview of the whale optimization algorithm

WOA is an excellent SIA developed by Mirjalili.^56^ WOA is inspired by the predation activities of humpback whales in nature in exploring prey, surrounding prey, and using bubble nets to attack prey. In this algorithm, the individual whale represents the potential agent, and the global optimal solution represents the prey. In general, WOA mainly completes location updates in the following three ways.

##### Encircling prey

During this phase, whales locate their prey and surround them. Since the initial best location is unknown, the WOA considers the current best agent as the prey. After identifying the prey, other whales in the population will update their position based on the current prey location. The mathematical model of this behavior is shown in Equations 1 and 2 :

(Equation 1) $$\begin{array}{c} D=\left|C\cdot X^{*}\left(t\right)-X\left(t\right)\right| \end{array}$$

(Equation 2) $$\begin{array}{c} X\left(t+1\right)=X^{*}\left(t\right)-A\cdot D \end{array}$$

where t represents the current iteration, X(t) stands for the current agent, and $X^{*}\left(t\right)$ means the optimal position of the humpback whale in the group in the current iteration. D represents the distance between the optimal individual in the current state and the current individual of population. A and C are two vectors of coefficients.

The parameters A and C are calculated as:

(Equation 3) $$\begin{array}{c} A=2a\cdot r_{1}-a \end{array}$$

(Equation 4) $$\begin{array}{c} C=2\cdot r_{1} \end{array}$$

where a decreases linearly from 2 to 0 in the whole search process, and $r_{1}$ is a random value between [0,1].

##### Bubble-net attacking (exploitation phase)

At the step, the algorithm randomly uses the two behavioral mechanisms of humpback whale contraction bounding and bubble net predation with 50% probability. Among them, when the bubble net is used for predation, the position update of the whale is expressed by the logarithmic spiral equation. The mathematical model is shown in Equation 5:

(Equation 5) $$\begin{array}{c} X\left(t+1\right)=D\cdot e^{bl}\cdot \cos\left(2\pi l\right)+X^{*}\left(t\right) \end{array}$$

where b is a constant with value 1, and l is a random value between [−1,1].

##### Search for prey (exploration phase)

In the exploration phase, humpback whales randomly search for prey in the search space. Mathematical models such as Equations 6 and 7 :

(Equation 6) $$\begin{array}{c} D=\left|C\cdot X_{rand}-X\right| \end{array}$$

(Equation 7) $$\begin{array}{c} X\left(t+1\right)=X_{rand}-A\cdot D \end{array}$$

where $X_{rand}$ represents a randomly selected position from the current population and X indicates the current location of the search agent.

#### Overview of kernel extreme learning machine (KELM)

Kernel extreme learning machine (KELM)^7^ is a widely researched learning algorithm that originated from extreme learning machine (ELM).^2^ Compared with traditional neural network algorithms, ELM has emerged as a research hotspot in recently due to its faster training speed and higher generalization capability. Nevertheless, ELM has the defects of requiring manual adjustment of parameters and easy to be trapped by local optimum. The new KELM method comes into being. KELM strengthens the convergence speed and generalization of ELM by combining kernel functions.

The single hidden layer feedforward neural networks can be expressed as Equation 8:

(Equation 8) $$\begin{array}{c} f\left(x\right)=h\left(x\right)\beta =H\beta =T \end{array}$$

where is the input vector, h(x), H stands for the hidden layer output matrix, β is the output weight, and T is the desired output. In ELM, β is expressed as Equation 9:

(Equation 9) $$\begin{array}{c} \beta ={\left(HH^{T}+\frac{I}{C}\right)}^{-1}\cdot H^{T}T \end{array}$$

where C is the regularization factor and I is the identity matrix.

Hence, ELM is represented by Equation 10:

(Equation 10) $$\begin{array}{c} f\left(x\right)=h\left(x\right)\beta =h\left(x\right)H^{T}{\left(HH^{T}+\frac{I}{C}\right)}^{-1}T \end{array}$$

In KELM, the kernel function is introduced to replace the output matrix of the hidden layer in ELM, and its mathematical model is represented by Equations 11 and 12.

(Equation 11) $$\begin{array}{c} f\left(x\right)=h\left(x\right)H^{T}{\left(HH^{T}+\frac{I}{C}\right)}^{-1}T=\left[\begin{array}{c} K\left(x,x_{1}\right)\\ \vdots \\ K\left(x,x_{n}\right) \end{array}\right]{\left(\Omega_{k}+\frac{I}{C}\right)}^{-1}T \end{array}$$

(Equation 12) $$\begin{array}{c} \left\{ \begin{array}{c} \Omega_{K}=HH^{T}\\ \Omega_{K,j}=h\left(x\right)\cdot h\left(x_{j}\right)=K\left(x_{i},x_{j}\right) \end{array}\right. \end{array}$$

where $H^{T}$ is the transpose matrix of the output matrix of the hidden layer, $\Omega_{K}$ is the kernel matrix, i,j∈(1,2,·,n), $K\left(x_{i},x_{j}\right)$ is the kernel function, and $x_{i}$ and $x_{j}$ represent the factor in the ith row and jth column of the kernel matrix $\Omega_{K}$, respectively.

Common kernel functions consist of linear kernel function, polynomial kernel function, and radial basis kernel function (RBF). In the proposed model, RBF is used, and its function expression is as shown in Equation 13:

(Equation 13) $$\begin{array}{c} K\left(u,v\right)=\exp\left(-\gamma {\left|\left|u-v\right|\right|}^{2}\right)\;\gamma >0 \end{array}$$

where γ is the kernel parameter and C balances the fitting error and the model complexity.

#### The proposed methodology

Although WOA has excellent convergence accuracy and convergence speed when facing global optimization situations, it may easily drop into the trap of local optimum (LO) when solving optimization problems with high complexity, such as feature selection (FS), its ability to explore and exploit needs to be improved. Therefore, WOA combines some strategies to overcome its shortcomings. This section will elaborate on the basic preparatory knowledge of the proposed SWEWOA and its application mechanism in detail, namely the wormhole search mechanism (WS), sine mapping initialization strategy (SS), and the added adaptive parameter E as the escape energy of prey (EE). Escape energy (EE) is a critical parameter between WOA exploration and exploitation transformation, which can help humpback whales choose reasonable behaviors with less cost.

##### The sine mapping initialization strategy *(SS)*

Chaotic sequences have randomness, ergodicity, and sensitivity to initial values, and can accelerate the algorithm to find the optimal solution. In the article, the population is initialized by chaotic sequences of sine mapping so that the solutions are dispersed as evenly as possible in the solution space. The quality of the initial solutions is improved so as to improve the convergence accuracy. The mathematical model of generating chaotic sequence based on sine mapping is shown in Equation 14 :

(Equation 14) $$\begin{array}{c} X_{i}=\left\{ \begin{array}{c} \left(UB-LB\right)\cdot r_{2}+LB\;if\;i=1\\ \frac{4}{\alpha }\sin\left(\pi \cdot X_{i-1}\right)\;if\;i>1 \end{array}\right. \end{array}$$

where UB and LB limit the boundaries of the search region, $r_{2}$ and α are random numbers with values varying from 0 to 1 and from 0 to 4, respectively.

##### The wormhole search mechanism*(WS)*

In the MVO, the wormhole search mechanism is designed to easily lead the swarm to dig deeper for the best individuals in the local space to uncover the potential optimal solution. In other words, by increasing the diversity of the swarm, the mechanism helps the population run away from the local optimum prematurely, thus improving the exploitation ability of the algorithm. *WEP* and *TDR* are two adaptive parameters, the former is used to determine the update method of location, while the latter represents the importance of the current candidate solution; the WS as expressed in Equations 15, 16, and 17 :

(Equation 15) $$\begin{array}{c} X_{j}^{i}\left(t+1\right)=\left\{ \begin{array}{c} \left\{ \begin{array}{c} X_{j}+TDR\times \left(\left(UB-LB\right)\times r_{5}+LB\right)\;r_{4}<0.5\\ X_{j}-TDR\times \left(\left(UB-LB\right)\times r_{5}+LB\right)\;r_{4}\geq 0.5 \end{array}\right.\;r_{3}<0.5\\ X_{i}^{j}\left(t\right)\;r_{3}\geq 0.5 \end{array}\right. \end{array}$$

(Equation 16) $$\begin{array}{c} WEP={WEP}_{\min}+FEs\times \left(\frac{{WEP}_{\max}-{WEP}_{\min}}{MaxFEs}\right) \end{array}$$

(Equation 17) $$\begin{array}{c} TDR=1-\frac{{FEs}^{\frac{1}{k}}}{{MaxFEs}^{\frac{1}{k}}} \end{array}$$

where k can control the local search capability, the larger the value of k, the more advantages in local space search, which is set to 6 in this paper.^88^ The range of WEP is between ${WEP}_{\min}$ and ${WEP}_{\max}$. In this paper, ${WEP}_{\min}$ is set to 0.2 and ${WEP}_{\max}$ is set to 1^95^. $r_{3}{，r}_{4}，r_{5}$ is a random number between [0，1]. FEs indicates the current count of evaluations and MaxFEs is the maximum count of evaluations.

##### Escaping energy *(EE)*

Heidari et al.^43^ used the energy of prey to transform the HHO algorithm between different behaviors during exploration and exploitation. Mathematically, escape energy is represented by equation Equation 18 :

(Equation 18) $$\begin{array}{c} E=2E_{0}\left(1-\frac{FEs}{MaxFEs}\right) \end{array}$$

(Equation 19) $$\begin{array}{c} E_{0}=2rand-1 \end{array}$$

where $E_{0}$ stands for the energy of the prey when it starts to be chased, which is a random number between [−1,1], and E represents the prey energy during the hunt. In the initial stage, the prey's energy is abundant, but as the search progresses, E is consumed and gradually decreases.

##### The proposed SWEWOA

In the cause of improving the capability of WOA to cope with complex combinatorial problems such as FS, a novel SIA called SWEWOA is proposed.

In the initialization phase, the SS is introduced in SWEWOA to improve the quality of the initial solutions to make whale individuals used for better search directions. Then, the wormhole search strategy is introduced as a search mechanism to help the original algorithm escape from the local optimum, and the behavior transformation between exploration and development is completed by escaping energy E. The optimization process of SWEWOA is as follows:

(1) Initialization parameters;
(2) The SS replaces the conventional population random initialization method. Using Equation 14;
(3) Calculate the fitness value for each individual;
(4) The escape energy E is updated using Equations 18 and 19;
(5) If |E|≥1, with a 50% probability in the surrounding prey or random search prey choice in these two behaviors, using Equation 2 or Equation 7 for a position update. If |E|<1, then with a 50% probability in the bubble net hunt or wormhole search mechanism of the two strategies to make a choice, using Equation 5 or Equation 15 for a position update.
(6) If the loop ending condition is not satisfied, then return to the third step; otherwise, the position of the currently found optimal solution and its fitness value are returned.

The computational complexity of SWEWOA relies on the maximum evaluation times (*MaxFEs*), the overall size (*N*), and the dimension of the objective function (*Dim*). Max_iteration is Max_iteration=(MaxFEs−N)/(2.05×N). SWEWOA consists of SS, WOA, WS, and EE. Escape energy EE is an adaptive parameter, so the computational complexity of SWEWOA is mainly affected by SS, WOA, and WS. O(SS)=O(N×Dim), $O\left(WOA\right)=O\left(Max_{iteration}\times N\times \mathrm{Dim}\right),O\left(WS\right)=O(\mathrm{Max}\_iteration\times N\times \mathrm{Dim}\text{.}$ So O(SWEWOA)=O(N×Dim+Max_iteration(2×N×Dim)).

The pseudocode of SWEWOA is displayed in Appendix A of supplemental information, and the flow chart of SWEWOA is displayed in below figure.

**Figure:**
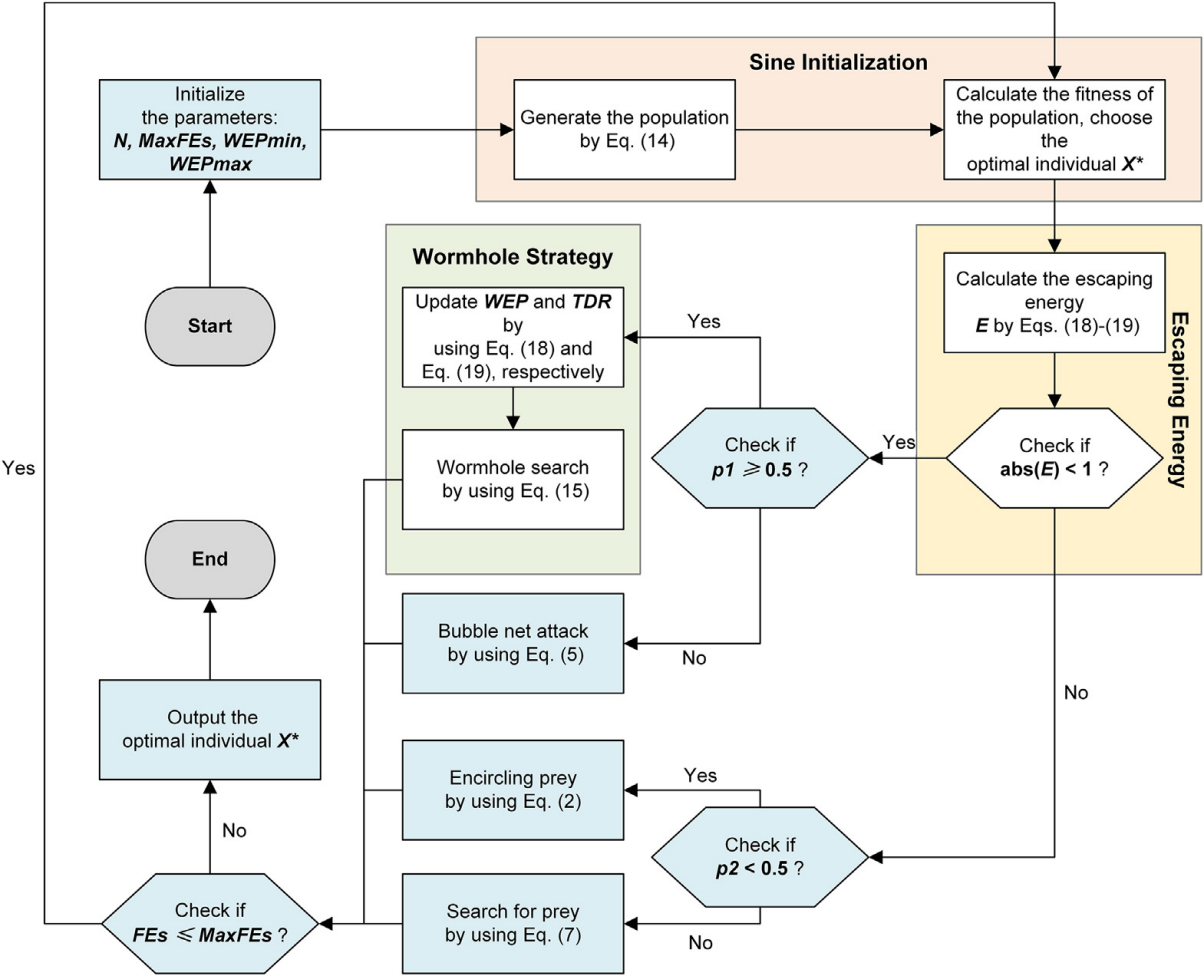
Flowchart of the SWEWOA

##### The new suggested feature selection method

###### Fitness function

The wrapped method evaluates the feature subset according to the performance of the model. Therefore, we need to construct a reasonable fitness function for feature selection. This research uses the traditional evaluation method of combining classification error rate and feature subset. The fitness function is expressed as Equation 20.

(Equation 20) $$\begin{array}{c} fitness=\omega \cdot error+\theta \cdot \frac{R}{L} \end{array}$$

where error represents the classification error rate, error=1−accuracy. R represents the size of obtained features, and L represents the size of total features. ω and θ are the two weight coefficients. Compared with the chosen features, the impact of the error rate on the classification results is more important. In this study, the two weight coefficients were set as ω=0.99 and θ=0.01. For the feature subset, lower fitness value indicates the stronger classification performance.

###### The ten-fold cross-validation

In order to avoid the special division of the training set and test set, the generalization capability of the model is reduced. In this study, the ten-fold cross-validation is employed to fully partition the existing data sets for many times.

This method distributes the dataset into 10 disjoint subsets of the same size, nine of which are devoted to training and remaining one subset is used for validation, and the performance is measured. Repeat this process until all 10 subsets have been used as validation sets. The average value of 10 validation outcomes was utilized as the final result. The ten-fold cross-validation is displayed in below figure.

**Figure:**
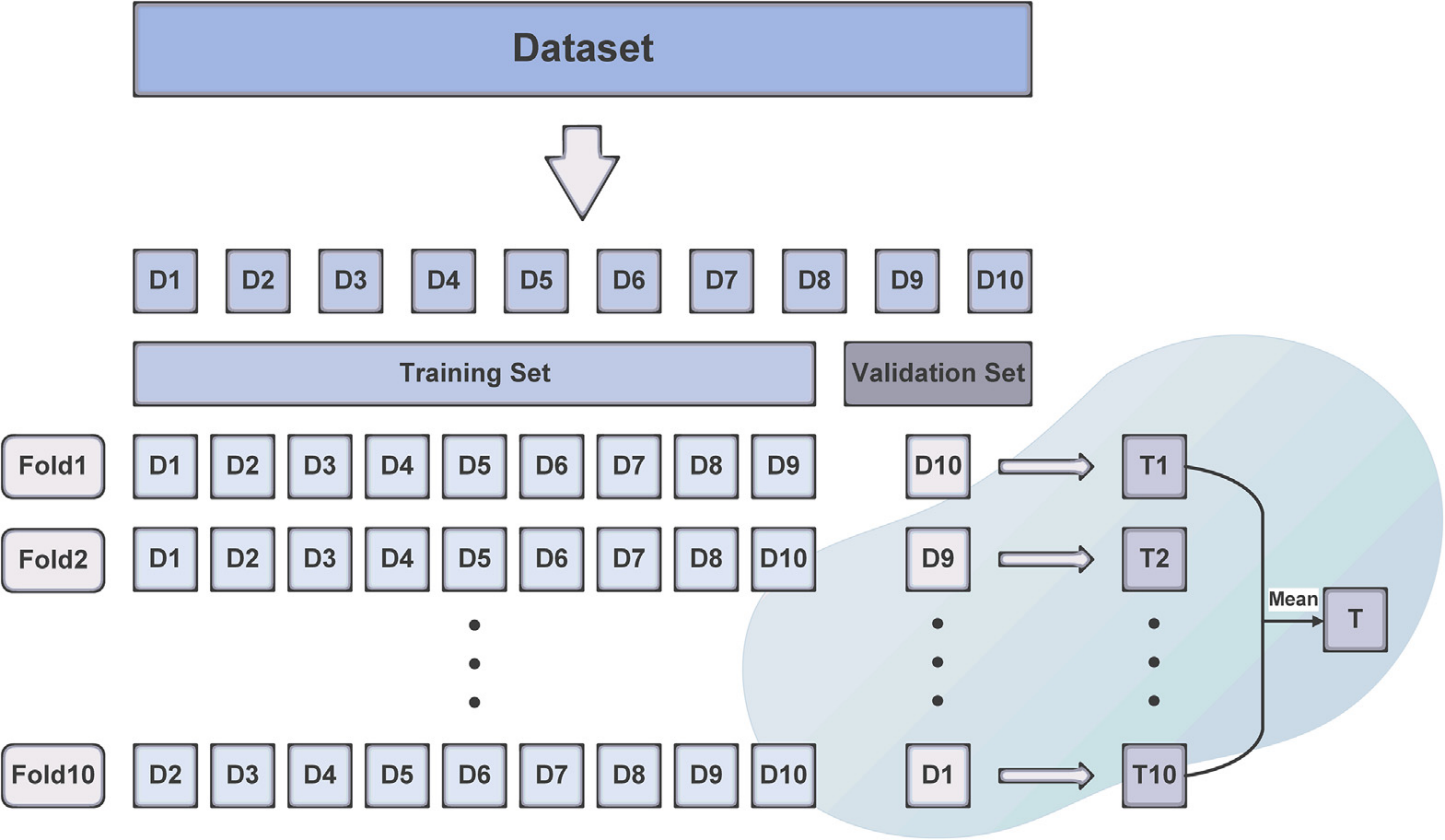
10-fold cross-validation procedure

###### The presented SWEWOA-KELM

Feature selection is a problem of binary classification where each feature appears with only two outcomes (chosen and unchosen). Therefore, this section needs to improve SWEWOA to a binary version (BSWEWOA). All features are numbers with a 0 or 1, where 0 and 1 represent unselected and selected features, respectively. Then the continuous space is converted into a discrete space by Equations 21 and 22.

(Equation 21) $$\begin{array}{c} X_{d}\left(t+1\right)=\left\{ \begin{array}{c} ∼X_{d,}\;sigmoid\left(X_{d}\left(t\right)\right)\geq r\\ X_{d},otherwise \end{array}\right. \end{array}$$

(Equation 22) $$\begin{array}{c} sigmoid\left(x\right)=\left|\frac{2}{\pi }\arctan\left(\frac{2}{\pi }\cdot x\right)\right| \end{array}$$

where r is a random value between [0,1] and $X_{d}$ is the binary position of the search agent.

A new machine learning framework is presented, combining SWEWOA and KELM. The framework involves two primary elements. The first component mainly focuses on the optimization of two important parameters C, γ, and the selection of features in KELM. The next component is to evaluate the test set samples. In the selection process of internal parameters and features, the SWEWOA strategy is used to dynamically adjust the best parameters and critical features of the training set. Then, the best parameters and feature subsets are input into the KELM, and the ten-fold cross-validation was utilized to assess the classification performance.

The proposed SWEWOA-KELM flow chart is drawn in below figure.

**Figure:**
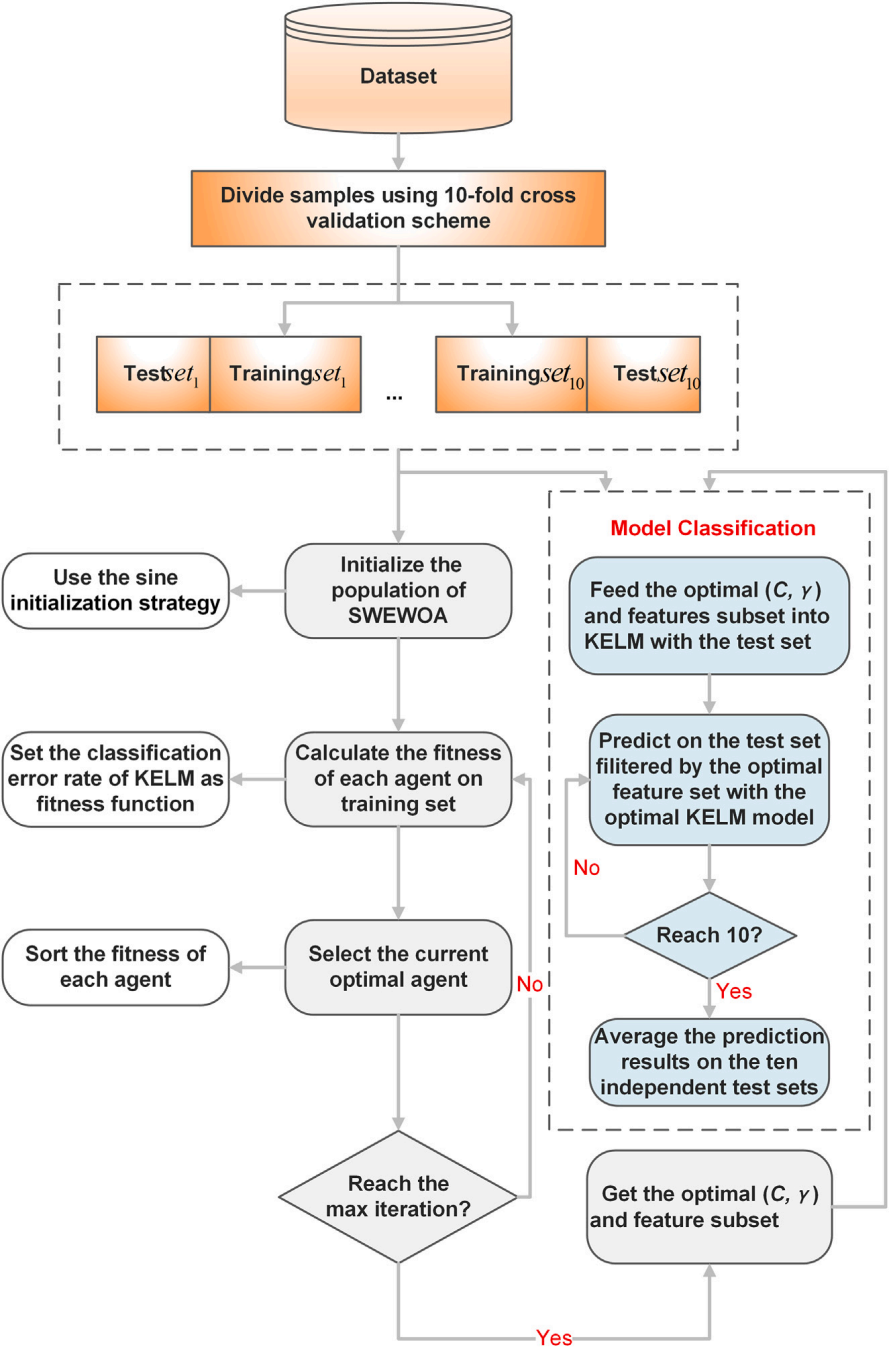
Flowchart of the BSWEWOA-KELM

### Quantification and statistical analysis

Detailed description of statistical methods is provided in experimental results and discussion under the following sections: global optimization experiment and feature selection experiment. The overall experiments are conducted in the same hardware and MATLAB R2018a software environment. Global optimisation experiments include strategy comparisons, comparisons with some classical original algorithms and comparisons with several variants of algorithms. All algorithms evaluated their performance using the statistical average value of the optimal function (Avg) and standard deviation (Std). The smaller the value, the better the performance. The Wilcoxon signed-rank test is used to evaluate the significance of differences between algorithms. If the result of the Wilcoxon signed-rank test is less than 0.05, that is, the p-value is less than 0.05, then there is a significant difference in performance between the methods. In addition, the Friedman test is used to analyse the statistical results obtained in this paper. The symbols “+/=/-” illustrate that the proposed algorithm performs better, equal, or worse than the other comparative method. All statistical details of global optimization are provided in Tables 4, 5, 6, 7, 8, 9, and C1–C10, and Figures 3, 4, 5, 6, 7, 8, 9, 10, 11, and 12. In terms of feature selection, datasets size information for all analyses is provided in Tables 10 and 17. The results are evaluated based on the mean and standard deviation of the fitness, average feature number, accuracy, MCC, F-measure. Tables 13, 14, 15, 16, 18, 19, 20, 21, and C11–C13 describe the statistical outcomes of the 14 high-dimensional gene datasets simulated by intelligent swarm algorithms. All statistical details are provided and explained in the text.

## Data Availability

• The dataset that informed or guided this study are available online and data reported in this paper will be shared by the lead contact upon request. • The original code is not reported in this paper. • Any additional information required to reanalyze the data reported in this paper is available from the lead contact upon request.

## Supplemental information

Document S1. Algorithm 1 and Tables B1, B2, and C1–C13

## References

[1] Qin X., Liu Z., Liu Y., Liu S., Yang B., Yin L., Liu M., Zheng W. (2022). User OCEAN Personality Model Construction Method Using a BP Neural Network. Electronics.

[2] Huang G.B., Zhu Q.Y., Siew C.K. (2006). Extreme learning machine: Theory and applications. Neurocomputing.

[3] Cao J., Zhang K., Luo M., Yin C., Lai X. (2016). Extreme learning machine and adaptive sparse representation for image classification. Neural Network..

[4] Zong W., Huang G.B. (2011). Face recognition based on extreme learning machine. Neurocomputing.

[5] Wan C., Xu Z., Pinson P., Dong Z.Y., Wong K.P. (2014). Probabilistic Forecasting of Wind Power Generation Using Extreme Learning Machine. IEEE Trans. Power Syst..

[6] Naji S., Keivani A., Shamshirband S., Alengaram U.J., Jumaat M.Z., Mansor Z., Lee M. (2016). Estimating building energy consumption using extreme learning machine method. Energy.

[7] Huang G.B., Zhou H., Ding X., Zhang R. (2012). Extreme Learning Machine for Regression and Multiclass Classification. IEEE Trans. Syst. Man Cybern. B Cybern..

[8] Wang M., Chen H., Yang B., Zhao X., Hu L., Cai Z., Huang H., Tong C. (2017). Toward an optimal kernel extreme learning machine using a chaotic moth-flame optimization strategy with applications in medical diagnoses. Neurocomputing.

[9] Chen H.L., Wang G., Ma C., Cai Z.N., Liu W.B., Wang S.J. (2016). An efficient hybrid kernel extreme learning machine approach for early diagnosis of Parkinson's disease. Neurocomputing.

[10] Liu T., Hu L., Ma C., Wang Z.Y., Chen H.L. (2015). A fast approach for detection of erythemato-squamous diseases based on extreme learning machine with maximum relevance minimum redundancy feature selection. Int. J. Syst. Sci..

[11] Lu J., Huang J., Lu F. (2019). Distributed Kernel Extreme Learning Machines for Aircraft Engine Failure Diagnostics. Appl. Sci..

[12] Luo J., Wang Y., Nakano T., Xu Y.T., Huang H., Zhao X.H. (2018). An improved grasshopper optimization algorithm with application to financial stress prediction. Polymers.

[13] Zhao D., Huang C., Wei Y., Yu F., Wang M., Chen H. (2017). An Effective Computational Model for Bankruptcy Prediction Using Kernel Extreme Learning Machine Approach. Comput. Econ..

[14] Le B.T., Ha T.T.L. (2020). Hyperspectral remote sensing image classification based on random average band selection and an ensemble kernel extreme learning machine. Appl. Opt..

[15] Pal M., Maxwell A.E., Warner T.A. (2013). Kernel-based extreme learning machine for remote-sensing image classification. Remote Sensing Letters.

[16] Chen C., Li W., Su H., Liu K. (2014). Spectral-Spatial Classification of Hyperspectral Image Based on Kernel Extreme Learning Machine. Rem. Sens..

[17] Lv L., Wang W., Zhang Z., Liu X. (2020). A novel intrusion detection system based on an optimal hybrid kernel extreme learning machine. Knowl. Base Syst..

[18] Deng W.Y., Zheng Q.H., Wang Z.M. (2014). Cross-person activity recognition using reduced kernel extreme learning machine. Neural Network..

[19] Liu B., Tang L., Wang J., Li A., Hao Y. (2014). 2-D defect profile reconstruction from ultrasonic guided wave signals based on QGA-kernelized ELM. Neurocomputing.

[20] Zhao X., Li D., Yang B., Liu S., Pan Z., Chen H. (2016). An Efficient and Effective Automatic Recognition System for Online Recognition of Foreign Fibers in Cotton. IEEE Access.

[21] Cai Z., Gu J., Luo J., Zhang Q., Chen H., Pan Z., Li Y., Li C. (2019). Evolving an optimal kernel extreme learning machine by using an enhanced grey wolf optimization strategy. Expert Syst. Appl..

[22] Lu H., Du B., Liu J., Xia H., Yeap W.K. (2017). A kernel extreme learning machine algorithm based on improved particle swam optimization. Memet. Comput..

[23] Li X., Sun Y. (2021). Application of RBF neural network optimal segmentation algorithm in credit rating. Neural Comput. Appl..

[24] Bolón-Canedo V., Sánchez-Maroño N., Alonso-Betanzos A. (2013). A review of feature selection methods on synthetic data. Knowl. Inf. Syst..

[25] Ke W., Wu C., Wu Y., Xiong N.N. (2018). A New Filter Feature Selection Based on Criteria Fusion for Gene Microarray Data. IEEE Access.

[26] Cui X., Li Y., Fan J., Wang T. (2022). A novel filter feature selection algorithm based on relief. Appl. Intell..

[27] Hancer E., Xue B., Zhang M. (2018). Differential evolution for filter feature selection based on information theory and feature ranking. Knowl. Base Syst..

[28] Li W., Chen L., Zhao J., Wang W. (2022). Embedded Feature Selection Based on Relevance Vector Machines With an Approximated Marginal Likelihood and Its Industrial Application. IEEE Trans. Syst. Man Cybern. Syst..

[29] Zhu Q.H., Yang Y.B. (2018). Discriminative embedded unsupervised feature selection. Pattern Recogn. Lett..

[30] Chen G., Chen J. (2015). A novel wrapper method for feature selection and its applications. Neurocomputing.

[31] Zhang Y., Wang S., Phillips P., Ji G. (2014). Binary PSO with mutation operator for feature selection using decision tree applied to spam detection. Knowl. Base Syst..

[32] Faris H., Mafarja M.M., Heidari A.A., Aljarah I., Al-Zoubi A.M., Mirjalili S., Fujita H. (2018). An efficient binary Salp Swarm Algorithm with crossover scheme for feature selection problems. Knowl. Base Syst..

[33] Zhang X., Wen S., Yan L., Feng J., Xia Y. (2022). A Hybrid-Convolution Spatial–Temporal Recurrent Network For Traffic Flow Prediction. Comput. J..

[34] Yang X., Zhao D., Yu F., Heidari A.A., Bano Y., Ibrohimov A., Liu Y., Cai Z., Chen H., Chen X. (2022). An optimized machine learning framework for predicting intradialytic hypotension using indexes of chronic kidney disease-mineral and bone disorders. Comput. Biol. Med..

[35] Luo J., Chen H., Hu Z., Huang H., Wang P., Wang X., Lv X.-E., Wen C. (2019). A new kernel extreme learning machine framework for somatization disorder diagnosis. IEEE Access.

[36] Cao B., Fan S., Zhao J., Tian S., Zheng Z., Yan Y., Yang P. (2021). Large-scale many-objective deployment optimization of edge servers. IEEE Trans. Intell. Transport. Syst..

[37] Cao B., Zhao J., Lv Z., Yang P. (2021). Diversified personalized recommendation optimization based on mobile data. IEEE Trans. Intell. Transport. Syst..

[38] Cao B., Li M., Liu X., Zhao J., Cao W., Lv Z. (2021). Many-objective deployment optimization for a drone-assisted camera network. IEEE Trans. Netw. Sci. Eng..

[39] Li B., Tan Y., Wu A.-G., Duan G.-R. (2022). A distributionally robust optimization based method for stochastic model predictive control. IEEE Trans. Automat. Control.

[40] Mirjalili S., Dong J.S., Lewis A. (2019).

[41] Cao B., Gu Y., Lv Z., Yang S., Zhao J., Li Y. (2021). RFID Reader Anticollision Based on Distributed Parallel Particle Swarm Optimization. IEEE Internet Things J..

[42] Kaur S., Awasthi L.K., Sangal A., Dhiman G. (2020). Tunicate Swarm Algorithm: A new bio-inspired based metaheuristic paradigm for global optimization. Eng. Appl. Artif. Intell..

[43] Heidari A.A., Mirjalili S., Faris H., Aljarah I., Mafarja M., Chen H. (2019). Harris hawks optimization: Algorithm and applications. Future Generat. Comput. Syst..

[44] Pan W.T. (2012). A new fruit fly optimization algorithm: taking the financial distress model as an example. Knowl. Base Syst..

[45] Li S., Chen H., Wang M., Heidari A.A., Mirjalili S. (2020). Slime mould algorithm: A new method for stochastic optimization. Elsevier.

[46] Yang Y., Chen H., Heidari A.A., Gandomi A.H. (2021). Hunger games search: Visions, conception, implementation, deep analysis, perspectives, and towards performance shifts. Expert Syst. Appl..

[47] Ahmadianfar I., Heidari A.A., Noshadian S., Chen H., Gandomi A.H. (2022).

[48] Ahmadianfar I., Heidari A.A., Gandomi A.H., Chu X., Chen H. (2021). RUN beyond the metaphor: An efficient optimization algorithm based on Runge Kutta method. Expert Syst. Appl..

[49] Tu J., Chen H., Wang M., Gandomi A.H. (2021). The Colony Predation Algorithm. J. Bionic Eng..

[50] Issa M., Hassanien A.E., Oliva D., Helmi A., Ziedan I., Alzohairy A. (2018). ASCA-PSO: Adaptive sine cosine optimization algorithm integrated with particle swarm for pairwise local sequence alignment. Expert Syst. Appl..

[51] Nenavath H., Jatoth R.K. (2018). Hybridizing sine cosine algorithm with differential evolution for global optimization and object tracking. Appl. Soft Comput..

[52] Zhang Y., Cui G., Wu J., Pan W.T., He Q. (2016). A novel multi-scale cooperative mutation Fruit Fly Optimization Algorithm. Knowl. Base Syst..

[53] Singh N., Singh S.B. (2017). A novel hybrid GWO-SCA approach for optimization problems. Eng. Sci. Technol. Int. J..

[54] Zhu A., Xu C., Li Z., Wu J., Liu Z. (2015). Hybridizing grey wolf optimization with differential evolution for global optimization and test scheduling for 3D stacked SoC. J. Syst. Eng. Electron..

[55] Li H., Liu J., Chen L., Bai J., Sun Y., Lu K. (2019). Chaos-enhanced moth-flame optimization algorithm for global optimization. J. Syst. Eng. Electron..

[56] Mirjalili S., Lewis A. (2016). The Whale Optimization Algorithm. Adv. Eng. Software.

[57] Li X., Berahovich R., Zhou H., Liu X., Li F., Xu S., Wei Y., Ouaret D., Bodmer W., Wu L., Golubovskaya V. (2020). Stock intelligent investment strategy based on support vector machine parameter optimization algorithm. Front. Biosci..

[58] Yousri D., Allam D., Eteiba M.B. (2019). Chaotic whale optimizer variants for parameters estimation of the chaotic behavior in Permanent Magnet Synchronous Motor. Appl. Soft Comput..

[59] Elhosseini M.A., Haikal A.Y., Badawy M., Khashan N. (2019). Biped robot stability based on an A-C parametric Whale Optimization Algorithm. J. Comput. Sci..

[60] Abd Elaziz M., Oliva D. (2018). Parameter estimation of solar cells diode models by an improved opposition-based whale optimization algorithm. Energy Convers. Manag..

[61] Qiao S., Yu H., Heidari A.A., El-Saleh A.A., Cai Z., Xu X., Mafarja M., Chen H. (2022). Individual disturbance and neighborhood mutation search enhanced whale optimization: performance design for engineering problems. J. Comput. Des. Eng..

[62] Yu H., Qiao S., Heidari A.A., Bi C., Chen H.J.M. (2022). Individual Disturbance and Attraction Repulsion Strategy Enhanced Seagull Optimization for Engineering Design. Mathematics.

[63] Liu X.-F., Zhan Z.-H., Gao Y., Zhang J., Kwong S., Zhang J. (2019). Coevolutionary particle swarm optimization with bottleneck objective learning strategy for many-objective optimization. IEEE Trans. Evol. Comput..

[64] Chen Y., Wang M., Heidari A.A., Shi B., Hu Z., Zhang Q., Chen H., Mafarja M., Turabieh H. (2022). Multi-threshold image segmentation using a multi-strategy shuffled frog leaping algorithm. Expert Syst. Appl..

[65] Qi A., Zhao D., Yu F., Heidari A.A., Wu Z., Cai Z., Alenezi F., Mansour R.F., Chen H., Chen M. (2022). Directional mutation and crossover boosted ant colony optimization with application to COVID-19 X-ray image segmentation. Comput. Biol. Med..

[66] Zhao D., Liu L., Yu F., Heidari A.A., Wang M., Liang G., Muhammad K., Chen H. (2021). Chaotic random spare ant colony optimization for multi-threshold image segmentation of 2D Kapur entropy. Knowl. Base Syst..

[67] Zhao D., Qi A., Yu F., Heidari A.A., Chen H., Li Y. (2023). Multi-strategy ant colony optimization for multi-level image segmentation: Case study of melanoma. Biomed. Signal Process Control.

[68] Xing J., Zhou X., Zhao H., Chen H., Heidari A.A. (2023). Elite levy spreading differential evolution via ABC shrink-wrap for multi-threshold segmentation of breast cancer images. Biomed. Signal Process Control.

[69] Pang J., Zhou H., Tsai Y.C., Chou F.D. (2018). A scatter simulated annealing algorithm for the bi-objective scheduling problem for the wet station of semiconductor manufacturing. Comput. Ind. Eng..

[70] Zhang Y., Liu R., Wang X., Chen H., Li C. (2021). Boosted binary Harris hawks optimizer and feature selection. Eng. Comput..

[71] Li Q., Chen H., Huang H., Zhao X., Cai Z., Tong C., Liu W., Tian X. (2017). An Enhanced Grey Wolf Optimization Based Feature Selection Wrapped Kernel Extreme Learning Machine for Medical Diagnosis. Comput. Math. Methods Med..

[72] Peng L., Cai Z., Heidari A.A., Zhang L., Chen H. (2023).

[73] Wang M.J., Noel J.E., Li H., Cai Z., Zhao X., Tong C., Li J., Xu X. (2017). Grey wolf optimization evolving kernel extreme learning machine: Application to bankruptcy prediction. World J. Otorhinolaryngol. Head Neck Surg..

[74] Wolpert D.H., Macready W.G. (1997). No free lunch theorems for optimization. IEEE Trans. Evol. Comput..

[75] Tubishat M., Abushariah M.A., Idris N., Aljarah I.J.A.I. (2019). Improved whale optimization algorithm for feature selection in Arabic sentiment analysis. Appl. Intell..

[76] Chen H., Yang C., Heidari A.A., Zhao X. (2020). An efficient double adaptive random spare reinforced whale optimization algorithm. Expert Syst. Appl..

[77] Wu G., Mallipeddi R., Suganthan P.N. (2017).

[78] García S., Fernández A., Luengo J., Herrera F.J.I.S. (2010). Advanced nonparametric tests for multiple comparisons in the design of experiments in computational intelligence and data mining. Exp. Anal. Power.

[79] Derrac J., García S., Molina D., Herrera F., Computation E. (2011). A practical tutorial on the use of nonparametric statistical tests as a methodology for comparing evolutionary and swarm intelligence algorithms. Swarm Evol. Comput..

[80] Zhang K., Wang Z., Chen G., Zhang L., Yang Y., Yao C., Wang J., Yao J. (2022). Training effective deep reinforcement learning agents for real-time life-cycle production optimization. J. Petrol. Sci. Eng..

[81] Xu J., Pan S., Sun P.Z.H., Hyeong Park S., Guo K. (2023). Human-Factors-in-Driving-Loop: Driver Identification and Verification via a Deep Learning Approach using Psychological Behavioral Data. IEEE Trans. Intell. Transport. Syst..

[82] Chen H., Xu Y., Wang M., Zhao X. (2019).

[83] Nobile M.S., Cazzaniga P., Besozzi D., Colombo R., Mauri G., Pasi G. (2018). Fuzzy Self-Tuning PSO: A settings-free algorithm for global optimization. Swarm Evol. Comput..

[84] Jia H., Lang C., Oliva D., Song W., Peng X. (2019). Dynamic harris hawks optimization with mutation mechanism for satellite image segmentation. Rem. Sens..

[85] Mirjalili S., Mirjalili S.M., Lewis A. (2014). Grey Wolf Optimizer. Adv. Eng. Software.

[86] Yang X.S., Hossein Gandomi A. (2012). Bat algorithm: a novel approach for global engineering optimization. Eng. Comput..

[87] Hu J., Heidari A.A., Zhang L., Xue X., Gui W., Chen H., Pan Z. (2022). Chaotic diffusion-limited aggregation enhanced grey wolf optimizer: insights, analysis, binarization, and feature selection. Int. J. Intell. Syst..

[88] Mirjalili S., Mirjalili S.M., Hatamlou A. (2016). Multi-verse optimizer: a nature-inspired algorithm for global optimization. Neural Comput. Appl..

