# Supplementary material for: An accelerated sine mapping whale optimizer for feature selection
Source: iScience. 2023 Sep 14;26(10):107896. doi: 10.1016/j.isci.2023.107896 (PMC10582515; doi:10.1016/j.isci.2023.107896)
Supplement: Document S1. Algorithm 1 and Tables B1, B2, and C1–C13 [file mmc1.pdf]

iScience, Volume *26*

Supplemental information

An accelerated sine mapping whale optimizer for feature selection

Helong Yu, Zisong Zhao, Ali Asghar Heidari, Li Ma, Monia Hamdi, Romany F. Mansour, and Huiling Chen

Supplementary materials

Appendix A

Algorithm 1 The pseudocode of SWEWOA , Related to Figure 1.

Algorithm 1 The pseudo-code of the proposed SWEWOA

Initialize parameters $N, FEs, MaxFEs, WEP_{min}, WEP_{max}$.
$//SS$
Initialize the population of whale X by Eq. (14).
while $(FEs \leq MaxFEs)$ **do**
for $i = 1{:}\,size(X, 1)$**do**
Calculate the fitness value of each search agent.
end for
Set X^* as the best individual of whales.
Update WEP and TDR by Eq. (16) and Eq. (17), respectively.
for $i = 1{:}\,size(X, 1)$ **do**
Update a, C, l, p_1, p_2.
$//EE$
Update Initial energy E_0 and escaping energy E by Eq. (18) and Eq. (19), respectively.
for $j = 1{:}\,size(X, 2)$ **do**
if $|E| < 1$
if $p_1 < 0.5$
Update the position of the agent using Eq. (5).
else
$//WS$
Update the position of the agent using Eq. (15).
end if
else
if $p_2 < 0.5$
Update the position of the agent using Eq. (2).
else
Update the position of the agent using Eq. (7).
end if
end if
end for
end for
end while
Return X^*.

Appendix B

Table B 1. Description of IEEE CEC2017 benchmark functions, Related to Figure 4 to 14.

Class Name	No.	Functions	f_{min}
Unimodal	F_1	Shifted and Rotated Bent Cigar Function	100

Functions	F_2	Shifted and Rotated Sum of Different Power Function	200
	F_3	Shifted and Rotated Zakharov Function	300
Simple Multimodal Functions	F_4	Shifted and Rotated Rosenbrock's Function	400
	F_5	Shifted and Rotated Rastrigin's Function	500
	F_6	Shifted and Rotated Expanded Scaffer's F6 Function	600
	F_7	Shifted and Rotated Lunacek Bi_Rastrigin Function	700
	F_8	Shifted and Rotated Non-Continuous Rastrigin's Function	800
	F_9	Shifted and Rotated Levy Function	900
	F_{10}	Shifted and Rotated Schwefel's Function	1000
Hybrid Function	F_{11}	Hybrid Function 1 (N=3)	1100
	F_{12}	Hybrid Function 2 (N=3)	1200
	F_{13}	Hybrid Function 3 (N=3)	1300
	F_{14}	Hybrid Function 4 (N=4)	1400
	F_{15}	Hybrid Function 5 (N=4)	1500
	F_{16}	Hybrid Function 6 (N=4)	1600
	F_{17}	Hybrid Function 6 (N=5)	1700
	F_{18}	Hybrid Function 6 (N=5)	1800
	F_{19}	Hybrid Function 6 (N=5)	1900
	F_{20}	Hybrid Function 6 (N=6)	2000
Composition Functions	F_{21}	Composition Function 1 (N=3)	2100
	F_{22}	Composition Function 2 (N=3)	2200
	F_{23}	Composition Function 3 (N=4)	2300
	F_{24}	Composition Function 4 (N=4)	2400
	F_{25}	Composition Function 5 (N=5)	2500
	F_{26}	Composition Function 6 (N=5)	2600
	F_{27}	Composition Function 7 (N=6)	2700
	F_{28}	Composition Function 8 (N=6)	2800
	F_{29}	Composition Function 9 (N=3)	2900
	F_{30}	Composition Function 10 (N=3)	3000
Search Range: $[-100, 100]^{Dim}$			

Table B 2. Public parameter settings , Related to Table 1.

Public parameters	Name	Values
Maximum number of evaluations	*MaxFEs*	300,000
Objective function dimensions	*Dim*	30
Size of the population	*N*	30
The upper boundary of the search space	*Ub*	100
The lower boundary of the search space	*Lb*	-100
Number of independent runs	*Flod*	30

Table B 3. Description of IEEE CEC2022 benchmark functions, Related to Figure 15.

Class Name	No.	Functions	f_{min}
Unimodal Functions	F_1	Shifted and Rotated Zakharov Function	300

Basic Functions	F_2	Shifted and full Rotated Rosenbrock's Function	400
	F_3	Shifted and full Rotated Expanded Scaffer's F6 Function	600
	F_4	Shifted and full Rotated Non-Continuous Rastrigin's Function	800
	F_5	Shifted and full Rotated Levy Function	900
Hybrid Function	F_6	Hybrid Function 1 (N=3)	1800
	F_7	Hybrid Function 2 (N=6)	2000
	F_8	Hybrid Function 3 (N=5)	2200
Composition Functions	F_9	Composition Function 1 (N=5)	2300
	F_{10}	Composition Function 2 (N=4)	2400
	F_{11}	Composition Function 3 (N=5)	2600
	F_{12}	Composition Function 4 (N=6)	2700
Search Range: [-100, 100] ^{Dim}			

Appendix C

Table C 1. Mean value and standard deviation of stability experiment, Related to Table 6 to 7.

Fun	Items	*Dim*=10		*Dim*=30		*Dim*=50		*Dim*=100	
		SWEWOA	WOA	SWEWOA	WOA	SWEWOA	WOA	SWEWOA	WOA
F1	Avg	**3.73E+04**	8.91E+07	**1.02E+04**	3.22E+06	**8.72E+03**	7.05E+05	**7.18E+03**	3.51E+05
	Stdv	**1.59E+04**	7.26E+07	**6.29E+03**	2.35E+06	**6.58E+03**	7.71E+05	**7.34E+03**	5.85E+05
F2	Avg	**1.11E+06**	7.50E+30	**4.60E+02**	3.03E+20	**2.00E+02**	1.79E+20	**2.00E+02**	3.09E+16
	Stdv	**4.38E+06**	4.07E+31	**7.93E+02**	6.49E+20	**1.39E+00**	9.71E+20	**1.85E-04**	1.16E+17
F3	Avg	**3.04E+02**	2.13E+05	**3.00E+02**	1.62E+05	**3.00E+02**	1.39E+05	**3.00E+02**	8.18E+04
	Stdv	**1.49E+00**	5.29E+04	**6.93E-02**	5.61E+04	**2.77E-02**	4.97E+04	**1.10E-02**	4.02E+04
F4	Avg	**4.93E+02**	5.95E+02	**4.89E+02**	5.44E+02	**4.91E+02**	5.34E+02	**4.84E+02**	5.22E+02
	Stdv	**1.29E+01**	4.26E+01	**1.25E+01**	4.28E+01	**1.29E+01**	3.68E+01	**1.76E+01**	3.59E+01
F5	Avg	**6.29E+02**	8.10E+02	**6.26E+02**	7.90E+02	**6.03E+02**	7.68E+02	**6.00E+02**	7.67E+02
	Stdv	**5.22E+01**	5.61E+01	**3.34E+01**	6.01E+01	**2.28E+01**	4.99E+01	**2.71E+01**	4.56E+01
F6	Avg	**6.18E+02**	6.73E+02	**6.12E+02**	6.70E+02	**6.08E+02**	6.68E+02	**6.05E+02**	6.70E+02
	Stdv	**1.12E+01**	1.27E+01	1.13E+01	**1.11E+01**	**7.17E+00**	9.16E+00	8.77E+00	**1.23E+01**
F7	Avg	**8.47E+02**	1.25E+03	**8.31E+02**	1.24E+03	**8.25E+02**	1.23E+03	**8.26E+02**	1.20E+03
	Stdv	**3.12E+01**	8.82E+01	**3.36E+01**	8.70E+01	1.23E+03	**9.79E+01**	**3.17E+01**	8.92E+01
F8	Avg	**9.20E+02**	1.01E+03	**9.08E+02**	1.01E+03	**9.15E+02**	1.01E+03	**9.03E+02**	1.01E+03
	Stdv	1.60E+03	**4.08E+01**	**3.08E+01**	5.44E+01	**2.70E+01**	5.52E+01	**3.21E+01**	5.44E+01
F9	Avg	**3.69E+03**	8.63E+03	**2.48E+03**	7.61E+03	**2.41E+03**	6.92E+03	**1.95E+03**	7.42E+03
	Stdv	**6.22E+00**	2.89E+03	**1.20E+03**	2.01E+03	**1.49E+03**	2.02E+03	**1.22E+03**	2.05E+03
F10	Avg	**4.70E+03**	6.67E+03	**4.57E+03**	6.10E+03	**4.54E+03**	5.88E+03	**4.48E+03**	5.82E+03
	Stdv	**7.07E+02**	9.22E+02	**7.34E+02**	8.78E+02	**5.77E+02**	9.97E+02	**5.66E+02**	6.99E+02
F11	Avg	**1.30E+03**	3.24E+03	**1.28E+03**	1.54E+03	**1.26E+03**	1.37E+03	**1.23E+03**	1.28E+03
	Stdv	**6.61E+01**	1.18E+03	**4.88E+01**	1.57E+02	**5.90E+01**	8.79E+01	**5.47E+01**	7.41E+01
F12	Avg	**5.17E+06**	8.28E+07	**3.55E+06**	3.94E+07	**3.06E+06**	2.54E+07	**2.09E+06**	1.86E+07

	Stdv	**3.51E+06**	7.44E+07	**2.67E+06**	2.64E+07	**2.72E+06**	1.86E+07	**1.74E+06**	1.37E+07
F13	Avg	**1.70E+05**	2.89E+05	1.59E+05	**1.57E+05**	1.67E+05	**1.45E+05**	**1.34E+05**	1.35E+05
	Stdv	**1.29E+05**	6.16E+05	**8.82E+04**	1.02E+05	9.32E+04	**7.04E+04**	6.51E+04	**6.28E+04**
F14	Avg	**3.63E+04**	1.80E+06	**1.39E+04**	7.61E+05	**8.04E+03**	6.40E+05	**7.79E+03**	2.99E+05
	Stdv	**2.72E+04**	1.91E+06	**8.40E+03**	1.10E+06	**4.11E+03**	7.43E+05	**4.86E+03**	3.20E+05
F15	Avg	**7.31E+04**	1.08E+05	**3.82E+04**	1.03E+05	**3.02E+04**	8.67E+04	**1.90E+04**	6.07E+04
	Stdv	**4.66E+04**	7.33E+04	**1.73E+04**	1.06E+05	**1.38E+04**	9.25E+04	**1.20E+04**	5.43E+04
F16	Avg	**2.67E+03**	3.85E+03	**2.63E+03**	3.62E+03	**2.41E+03**	3.54E+03	**2.45E+03**	3.33E+03
	Stdv	**3.22E+02**	4.62E+02	**2.87E+02**	4.61E+02	**3.35E+02**	4.95E+02	**2.59E+02**	3.30E+02
F17	Avg	**2.25E+03**	2.57E+03	**2.14E+03**	2.52E+03	**2.13E+03**	2.45E+03	**2.09E+03**	2.41E+03
	Stdv	**1.57E+02**	3.56E+02	2.45E+02	**1.98E+02**	**2.01E+02**	2.10E+02	**1.48E+02**	2.22E+02
F18	Avg	**2.56E+05**	5.25E+06	**2.06E+05**	3.52E+06	**2.06E+05**	2.01E+06	**1.90E+05**	1.48E+06
	Stdv	**1.91E+05**	5.99E+06	**2.13E+05**	3.52E+06	**1.52E+05**	1.88E+06	**1.55E+05**	1.13E+06
F19	Avg	**2.69E+04**	4.67E+06	**1.29E+04**	2.96E+06	**1.07E+04**	1.44E+06	**1.34E+04**	1.01E+06
	Stdv	**1.94E+04**	5.53E+06	**1.16E+04**	2.67E+06	**1.20E+04**	1.23E+06	**1.52E+04**	8.77E+05
F20	Avg	**2.51E+03**	2.78E+03	**2.44E+03**	2.73E+03	**2.39E+03**	2.70E+03	**2.31E+03**	2.62E+03
	Stdv	1.85E+02	**1.51E+02**	**1.58E+02**	2.01E+02	**1.58E+02**	2.16E+02	**1.76E+02**	2.06E+02
F21	Avg	**2.41E+03**	2.59E+03	**2.40E+03**	2.58E+03	**2.40E+03**	2.58E+03	**2.40E+03**	2.57E+03
	Stdv	**4.76E+01**	5.72E+01	**4.68E+01**	7.03E+01	**4.32E+01**	8.31E+01	**2.89E+01**	6.87E+01
F22	Avg	**3.25E+03**	6.46E+03	**2.842E+03**	6.65E+03	**3.08E+03**	7.16E+03	**3.11E+03**	6.46E+03
	Stdv	**1.76E+03**	2.39E+03	**1.41E+03**	1.97E+03	1.60E+03	**1.50E+03**	**1.69E+03**	2.00E+03
F23	Avg	**2.77E+03**	3.06E+03	**2.77E+03**	3.07E+03	**2.75E+03**	3.03E+03	**2.75E+03**	3.03E+03
	Stdv	**3.63E+01**	9.82E+01	**4.00E+01**	7.36E+01	**2.75E+01**	8.82E+01	**2.46E+01**	9.04E+01
F24	Avg	**2.93E+03**	3.19E+03	**2.93E+03**	3.18E+03	**2.92E+03**	3.18E+03	**2.92E+03**	3.18E+03
	Stdv	**3.04E+01**	9.38E+01	**2.93E+01**	9.54E+01	**2.40E+01**	8.49E+01	**3.10E+01**	8.33E+01
F25	Avg	**2.89E+03**	3.00E+03	**2.89E+03**	2.95E+03	**2.89E+03**	2.94E+03	**2.89E+03**	2.93E+03
	Stdv	**3.11E+00**	3.11E+01	**1.06E+01**	2.66E+01	**1.43E+00**	3.19E+01	**3.02E+00**	3.24E+01
F26	Avg	**3.90E+03**	7.46E+03	**4.15E+03**	7.75E+03	**4.22E+03**	7.66E+03	**4.37E+03**	7.63E+03
	Stdv	**1.06E+03**	1.22E+03	**1.08E+03**	1.21E+03	**9.31E+02**	1.27E+03	**7.95E+02**	1.03E+03
F27	Avg	**3.23E+03**	3.38E+03	**3.22E+03**	3.39E+03	**3.22E+03**	3.34E+03	**3.21E+03**	3.33E+03
	Stdv	**1.39E+01**	8.42E+01	**1.08E+01**	1.05E+02	**1.51E+01**	9.48E+01	**1.32E+01**	6.75E+01
F28	Avg	**3.23E+03**	3.37E+03	**3.22E+03**	3.31E+03	**3.22E+03**	3.28E+03	**3.20E+03**	3.26E+03
	Stdv	**2.67E+01**	4.72E+01	3.88E+01	**3.27E+01**	4.39E+01	**2.84E+01**	5.12E+01	**2.26E+01**
F29	Avg	**3.90E+03**	5.12E+03	**3.88E+03**	4.83E+03	**3.69E+03**	4.71E+03	**3.73E+03**	4.54E+03
	Stdv	**2.16E+02**	5.28E+02	**2.00E+02**	5.21E+02	**1.64E+02**	5.51E+02	**2.16E+02**	3.59E+02
F30	Avg	**5.36E+05**	2.57E+07	**2.10E+05**	1.14E+07	**1.39E+05**	9.48E+06	**6.60E+04**	5.86E+06
	Stdv	**3.45E+05**	1.84E+07	**1.52E+05**	8.68E+06	**7.33E+04**	7.57E+06	**3.22E+04**	3.39E+06

Table C 2. Comparative results of stability experiment, Related to Table 6 to 7.

Fun	*Dim*=10		*Dim*=30		*Dim*=50		*Dim*=100	
	P-value	Results	P-value	Results	P-value	Results	P-value	Results
F1	1.73E-06	+	1.73E-06	+	1.73E-06	+	2.88E-06	+
F2	1.73E-06	+	1.73E-06	+	1.73E-06	+	1.73E-06	+
F3	1.73E-06	+	1.73E-06	+	1.73E-06	+	1.73E-06	+

F4	1.73E-06	+	2.88E-06	+	1.49E-05	+	1.80E-05	+
F5	1.73E-06	+	1.73E-06	+	1.73E-06	+	1.73E-06	+
F6	1.73E-06	+	1.73E-06	+	1.73E-06	+	1.73E-06	+
F7	1.73E-06	+	1.73E-06	+	1.73E-06	+	1.73E-06	+
F8	2.35E-06	+	1.73E-06	+	1.73E-06	+	2.12E-06	+
F9	2.13E-06	+	2.13E-06	+	1.73E-06	+	1.73E-06	+
F10	2.88E-06	+	1.13E-05	+	1.64E-05	+	1.73E-06	+
F11	1.73E-06	+	1.92E-06	+	2.16E-05	+	4.68E-03	+
F12	1.92E-06	+	1.73E-06	+	5.21E-06	+	1.92E-06	+
F13	2.99E-01		9.10E-01		1.92E-01		9.59E-01	
F14	2.35E-06	+	1.92E-06	+	1.73E-06	+	1.73E-06	+
F15	7.19E-02		1.29E-03	+	7.51E-05	+	3.41E-05	+
F16	1.73E-06	+	1.73E-06	+	1.73E-06	+	2.35E-06	+
F17	4.20E-04	+	1.97E-05	+	4.45E-05	+	1.13E-05	+
F18	2.60E-06	+	3.18E-06	+	2.13E-06	+	5.75E-06	+
F19	1.92E-06	+	1.73E-06	+	1.73E-06	+	1.73E-06	+
F20	6.98E-06	+	2.60E-05	+	1.64E-05	+	3.11E-05	+
F21	1.92E-06	+	1.73E-06	+	1.73E-06	+	1.73E-06	+
F22	2.84E-05	+	3.18E-06	+	4.73E-06	+	1.49E-05	+
F23	1.73E-06	+	1.73E-06	+	1.73E-06	+	1.73E-06	+
F24	1.73E-06	+	1.73E-06	+	1.73E-06	+	1.73E-06	+
F25	1.73E-06	+	2.13E-06	+	1.73E-06	+	1.73E-06	+
F26	2.35E-06	+	2.13E-06	+	1.92E-06	+	1.73E-06	+
F27	1.73E-06	+	1.73E-06	+	1.73E-06	+	1.73E-06	+
F28	1.73E-06	+	1.73E-06	+	2.84E-05	+	5.75E-06	+
F29	1.73E-06	+	1.92E-06	+	1.92E-06	+	1.73E-06	+
F30	1.73E-06	+	1.73E-06	+	1.73E-06	+	1.73E-06	+
B/W/E	28/0/2		29/0/1		29/0/1		29/0/1	

Table C 3. Mean and standard deviation of the comparative experiment of the original algorithms for IEEE CEC2017, Related to Figure 6 to 8.

Fun	Items	SWEWOA	HHO	TSA	FA	PSO	SCA	MFO	SMA	WOA
F1	Avg	**1.07E+04**	5.97E+06	1.99E+10	1.45E+10	1.29E+08	1.21E+10	1.13E+10	2.59E+09	3.08E+06
	Stdv	**6.96E+03**	1.58E+06	7.74E+09	1.66E+09	1.82E+07	1.60E+09	8.27E+09	1.13E+09	2.08E+06
F2	Avg	**2.59E+02**	2.98E+10	**2.29E+44**	6.58E+33	2.68E+13	2.86E+34	4.06E+39	9.53E+28	5.58E+23
	Stdv	**1.56E+02**	7.32E+10	**1.25E+45**	1.13E+34	2.04E+13	1.37E+35	1.56E+40	4.78E+29	3.05E+24
F3	Avg	**3.00E+02**	8.83E+02	4.74E+04	6.13E+04	6.40E+02	3.69E+04	1.03E+05	3.86E+04	1.38E+05
	Stdv	**1.08E-01**	2.32E+02	1.23E+04	8.53E+03	3.93E+01	4.94E+03	7.13E+04	7.19E+03	5.57E+04
F4	Avg	4.90E+02	5.12E+02	3.91E+03	1.37E+03	**4.74E+02**	1.41E+03	1.43E+03	6.36E+02	5.44E+02
	Stdv	**1.29E+01**	3.84E+01	2.52E+03	1.21E+02	3.26E+01	2.16E+02	9.98E+02	8.94E+01	3.48E+01
F5	Avg	**6.19E+02**	7.17E+02	8.23E+02	7.62E+02	7.35E+02	7.84E+02	7.15E+02	7.15E+02	7.80E+02
	Stdv	3.40E+01	4.00E+01	6.80E+01	**1.23E+01**	2.65E+01	2.12E+01	4.93E+01	3.33E+01	5.29E+01
F6	Avg	**6.11E+02**	6.59E+02	6.71E+02	6.43E+02	6.51E+02	6.50E+02	6.39E+02	6.44E+02	6.73E+02
	Stdv	1.05E+01	6.01E+00	1.35E+01	**2.99E+00**	1.16E+01	4.30E+00	1.18E+01	7.13E+00	1.10E+01
F7	Avg	**8.39E+02**	1.19E+03	**1.23E+03**	1.38E+03	9.16E+02	1.12E+03	1.07E+03	1.08E+03	1.23E+03

	Stdv	3.12E+01	7.03E+01	9.68E+01	3.86E+01	**1.51E+01**	3.32E+01	1.45E+02	4.59E+01	8.10E+01
F8	Avg	**9.19E+02**	9.53E+02	**1.09E+03**	1.05E+03	9.91E+02	1.05E+03	1.03E+03	9.72E+02	1.01E+03
	Stdv	3.57E+01	2.39E+01	3.96E+01	**1.41E+01**	2.01E+01	2.02E+01	4.80E+01	2.27E+01	5.98E+01
F9	Avg	**2.95E+03**	5.99E+03	1.01E+04	5.49E+03	5.51E+03	5.70E+03	7.44E+03	5.49E+03	8.18E+03
	Stdv	1.42E+03	5.47E+02	3.46E+03	**4.52E+02**	2.30E+03	9.16E+02	2.82E+03	1.29E+03	2.45E+03
F10	Avg	**4.70E+03**	5.24E+03	**6.76E+03**	7.90E+03	6.38E+03	8.20E+03	5.45E+03	5.69E+03	6.01E+03
	Stdv	6.38E+02	6.98E+02	6.62E+02	3.21E+02	5.65E+02	**2.96E+02**	8.34E+02	6.55E+02	9.34E+02
F11	Avg	1.27E+03	**1.25E+03**	5.18E+03	3.50E+03	1.28E+03	2.01E+03	5.44E+03	1.59E+03	1.53E+03
	Stdv	5.09E+01	5.15E+01	1.97E+03	4.36E+02	**4.05E+01**	2.20E+02	3.99E+03	1.05E+02	1.54E+02
F12	Avg	**2.58E+06**	7.89E+06	2.79E+09	1.52E+09	2.64E+07	1.14E+09	2.90E+08	1.14E+08	3.67E+07
	Stdv	**1.84E+06**	4.16E+06	1.93E+09	2.84E+08	1.23E+07	2.45E+08	4.62E+08	6.14E+07	2.90E+07
F13	Avg	1.74E+05	1.96E+05	3.42E+09	5.81E+08	4.73E+06	4.12E+08	1.37E+08	1.46E+06	**1.66E+05**
	Stdv	1.24E+05	1.29E+05	4.67E+09	1.75E+08	1.31E+06	1.61E+08	4.92E+08	1.40E+06	**7.77E+04**
F14	Avg	1.24E+04	2.09E+04	8.68E+05	1.75E+05	**9.33E+03**	1.64E+05	1.34E+05	2.04E+05	**9.16E+05**
	Stdv	**5.01E+03**	2.14E+04	1.14E+06	5.84E+04	5.90E+03	1.56E+05	1.94E+05	1.11E+05	1.23E+06
F15	Avg	4.50E+04	5.02E+04	1.14E+08	7.21E+07	4.39E+05	9.90E+06	4.87E+04	**1.97E+04**	8.02E+04
	Stdv	2.35E+04	3.59E+04	1.74E+08	2.73E+07	1.47E+05	7.36E+06	3.21E+04	**1.11E+04**	6.88E+04
F16	Avg	**2.52E+03**	3.14E+03	3.21E+03	3.50E+03	2.92E+03	3.52E+03	3.06E+03	2.85E+03	3.44E+03
	Stdv	2.99E+02	3.27E+02	5.99E+02	**1.48E+02**	2.15E+02	2.17E+02	4.51E+02	2.64E+02	3.60E+02
F17	Avg	**2.26E+03**	2.45E+03	2.51E+03	2.53E+03	2.32E+03	2.38E+03	2.58E+03	2.27E+03	2.56E+03
	Stdv	1.76E+02	2.56E+02	5.91E+02	**1.32E+02**	1.85E+02	1.84E+02	3.16E+02	1.61E+02	2.63E+02
F18	Avg	2.29E+05	5.83E+05	1.03E+07	3.26E+06	**1.71E+05**	3.03E+06	1.41E+06	1.11E+06	2.28E+06
	Stdv	2.11E+05	5.15E+05	2.07E+07	1.49E+06	**9.71E+04**	1.48E+06	3.00E+06	2.23E+06	2.12E+06
F19	Avg	**1.05E+04**	1.42E+05	3.84E+08	9.07E+07	1.55E+06	2.28E+07	1.28E+07	5.14E+05	3.22E+06
	Stdv	**1.03E+04**	9.58E+04	9.56E+08	4.07E+07	6.11E+05	9.05E+06	3.66E+07	5.41E+05	2.41E+06
F20	Avg	**2.37E+03**	2.71E+03	2.77E+03	2.59E+03	2.68E+03	2.65E+03	2.70E+03	2.45E+03	2.69E+03
	Stdv	1.58E+02	1.44E+02	1.60E+02	**8.54E+01**	2.06E+02	1.19E+02	2.23E+02	1.22E+02	1.69E+02
F21	Avg	**2.41E+03**	2.52E+03	2.61E+03	2.54E+03	2.54E+03	2.56E+03	2.51E+03	2.47E+03	2.60E+03
	Stdv	**3.14E+01**	3.54E+01	6.88E+01	**1.29E+01**	4.27E+01	2.05E+01	4.07E+01	2.54E+01	6.97E+01
F22	Avg	**2.66E+03**	6.33E+03	**7.08E+03**	3.84E+03	4.66E+03	8.66E+03	6.98E+03	3.83E+03	6.62E+03
	Stdv	1.09E+03	1.92E+03	1.84E+03	**1.49E+02**	2.71E+03	2.24E+03	9.18E+02	2.14E+03	2.06E+03
F23	Avg	**2.76E+03**	3.07E+03	3.22E+03	2.91E+03	**3.14E+03**	3.00E+03	2.83E+03	2.85E+03	3.05E+03
	Stdv	2.62E+01	1.27E+02	1.39E+02	**1.61E+01**	**1.42E+02**	2.91E+01	3.89E+01	2.90E+01	1.06E+02
F24	Avg	**2.92E+03**	3.40E+03	3.36E+03	3.06E+03	**3.20E+03**	3.16E+03	2.98E+03	3.01E+03	3.18E+03
	Stdv	2.51E+01	9.42E+01	8.90E+01	**1.18E+01**	8.04E+01	3.09E+01	3.79E+01	3.72E+01	9.49E+01
F25	Avg	**2.89E+03**	2.90E+03	3.61E+03	3.57E+03	**2.91E+03**	3.20E+03	3.43E+03	2.99E+03	2.94E+03
	Stdv	**1.25E+00**	1.31E+01	4.03E+02	7.52E+01	2.47E+01	8.38E+01	7.94E+02	3.59E+01	2.89E+01
F26	Avg	**4.32E+03**	6.03E+03	**8.22E+03**	6.51E+03	5.58E+03	6.99E+03	5.96E+03	5.22E+03	7.81E+03
	Stdv	8.33E+02	1.85E+03	9.28E+02	**1.27E+02**	1.91E+03	2.55E+02	4.43E+02	8.15E+02	1.39E+03
F27	Avg	3.22E+03	3.31E+03	3.66E+03	3.34E+03	**3.22E+03**	3.40E+03	3.26E+03	3.26E+03	3.36E+03
	Stdv	**1.34E+01**	5.63E+01	1.80E+02	1.45E+01	1.20E+02	4.13E+01	3.27E+01	2.68E+01	7.24E+01
F28	Avg	**3.22E+03**	3.23E+03	4.53E+03	3.90E+03	3.25E+03	3.81E+03	4.19E+03	3.39E+03	3.34E+03
	Stdv	4.74E+01	3.28E+01	6.04E+02	1.07E+02	**2.41E+01**	1.19E+02	1.06E+03	4.53E+01	2.35E+02

F29	Avg	**3.74E+03**	4.24E+03	4.72E+03	4.70E+03	4.23E+03	4.68E+03	4.18E+03	4.00E+03	4.88E+03
	Stdv	1.86E+02	3.26E+02	4.59E+02	2.13E+02	2.99E+02	**1.68E+02**	3.07E+02	2.10E+02	3.69E+02
F30	Avg	**2.64E+05**	1.04E+06	9.73E+07	9.09E+07	3.44E+06	6.93E+07	6.84E+05	5.29E+06	9.36E+06
	Stdv	**1.67E+05**	5.16E+05	3.32E+08	3.12E+07	1.36E+06	2.27E+07	6.98E+05	2.91E+06	5.51E+06

Table C 4. Comparative results of the comparative experiment of the original algorithms for IEEE CEC2017, Related to Figure 6 to 8.

Fun	HHO		TSA		FA		PSO	
	P-value	Results	P-value	Results	P-value	Results	P-value	Results
F1	1.73E-06	+	1.73E-06	+	1.73E-06	+	1.73E-06	+
F2	1.73E-06	+	1.73E-06	+	1.73E-06	+	1.73E-06	+
F3	1.73E-06	+	1.73E-06	+	1.73E-06	+	1.73E-06	+
F4	2.58E-03	+	1.73E-06	+	1.73E-06	+	6.56E-02	
F5	1.73E-06	+	1.73E-06	+	1.73E-06	+	2.13E-06	+
F6	1.73E-06	+	1.73E-06	+	1.73E-06	+	1.73E-06	+
F7	1.73E-06	+	1.73E-06	+	1.73E-06	+	2.35E-06	+
F8	8.94E-04	+	1.73E-06	+	1.73E-06	+	3.18E-06	+
F9	1.73E-06	+	1.73E-06	+	2.13E-06	+	2.83E-04	+
F10	8.73E-03	+	1.73E-06	+	1.73E-06	+	2.88E-06	+
F11	1.06E-01		1.73E-06	+	1.73E-06	+	9.43E-01	
F12	4.29E-06	+	1.73E-06	+	1.73E-06	+	1.73E-06	+
F13	4.53E-01		1.73E-06	+	1.73E-06	+	1.73E-06	+
F14	2.89E-01		5.75E-06	+	1.73E-06	+	1.85E-02	-
F15	8.13E-01		1.73E-06	+	1.73E-06	+	1.73E-06	+
F16	6.98E-06	+	1.02E-05	+	1.73E-06	+	7.51E-05	+
F17	7.73E-03	+	2.11E-03	+	4.73E-06	+	1.92E-01	
F18	3.88E-04	+	1.92E-06	+	1.73E-06	+	4.28E-01	
F19	1.73E-06	+	1.73E-06	+	1.73E-06	+	1.73E-06	+
F20	2.88E-06	+	5.75E-06	+	2.35E-06	+	2.60E-05	+
F21	1.92E-06	+	1.73E-06	+	1.73E-06	+	1.73E-06	+
F22	4.73E-06	+	1.73E-06	+	2.77E-03	+	4.86E-05	+
F23	1.73E-06	+	1.73E-06	+	1.73E-06	+	1.73E-06	+
F24	1.73E-06	+	1.73E-06	+	1.73E-06	+	1.73E-06	+
F25	8.47E-06	+	1.73E-06	+	1.73E-06	+	8.19E-05	+
F26	2.61E-04	+	1.73E-06	+	1.73E-06	+	3.16E-03	+
F27	2.88E-06	+	1.73E-06	+	1.73E-06	+	5.98E-02	
F28	5.86E-01		1.73E-06	+	1.73E-06	+	9.84E-03	+
F29	6.98E-06	+	1.92E-06	+	1.73E-06	+	4.29E-06	+
F30	2.88E-06	+	1.73E-06	+	1.73E-06	+	1.73E-06	+
B/W/E	25/0/5		30/0/0		30/0/0		24/1/5	

Table C 5. Comparative results of the comparative experiment of the original algorithms for IEEE CEC2017, Related to Figure 6 to 8. (continued)

Fun	SCA		MFO		SMA		WOA	
	P-value	Results	P-value	Results	P-value	Results	P-value	Results

F1	1.73E-06	+	1.73E-06	+	1.73E-06	+	1.73E-06	+
F2	1.73E-06	+	1.73E-06	+	1.73E-06	+	1.73E-06	+
F3	1.73E-06	+	1.73E-06	+	1.73E-06	+	1.73E-06	+
F4	1.73E-06	+	1.73E-06	+	1.73E-06	+	1.73E-06	+
F5	1.73E-06	+	4.73E-06	+	2.35E-06	+	1.92E-06	+
F6	1.73E-06	+	3.18E-06	+	1.73E-06	+	1.73E-06	+
F7	1.73E-06	+	1.73E-06	+	1.73E-06	+	1.73E-06	+
F8	1.73E-06	+	1.92E-06	+	2.16E-05	+	3.52E-06	+
F9	3.52E-06	+	1.73E-06	+	5.22E-06	+	2.35E-06	+
F10	1.73E-06	+	7.71E-04	+	1.64E-05	+	2.16E-05	+
F11	1.73E-06	+	1.92E-06	+	1.73E-06	+	1.73E-06	+
F12	1.73E-06	+	2.35E-06	+	1.73E-06	+	1.73E-06	+
F13	1.73E-06	+	6.58E-01		8.47E-06	+	9.10E-01	
F14	1.73E-06	+	2.88E-06	+	1.73E-06	+	1.73E-06	+
F15	1.73E-06	+	8.13E-01		6.89E-05	-	3.00E-02	+
F16	1.73E-06	+	9.71E-05	+	3.88E-04	+	1.92E-06	+
F17	1.48E-02	+	5.29E-04	+	7.97E-01		2.83E-04	+
F18	1.73E-06	+	3.38E-03	+	3.59E-04	+	1.73E-06	+
F19	1.73E-06	+	2.13E-06	+	2.88E-06	+	1.73E-06	+
F20	8.47E-06	+	1.36E-05	+	3.87E-02	+	4.73E-06	+
F21	1.73E-06	+	1.73E-06	+	6.98E-06	+	1.73E-06	+
F22	1.73E-06	+	1.92E-06	+	1.97E-05	+	1.73E-06	+
F23	1.73E-06	+	3.18E-06	+	1.73E-06	+	1.73E-06	+
F24	1.73E-06	+	5.75E-06	+	1.73E-06	+	1.73E-06	+
F25	1.73E-06	+	1.73E-06	+	1.73E-06	+	1.73E-06	+
F26	1.73E-06	+	2.13E-06	+	5.29E-04	+	2.35E-06	+
F27	1.73E-06	+	2.35E-06	+	3.52E-06	+	1.73E-06	+
F28	1.73E-06	+	1.73E-06	+	1.73E-06	+	8.47E-06	+
F29	1.73E-06	+	2.88E-06	+	4.20E-04	+	1.73E-06	+
F30	1.73E-06	+	1.17E-02	+	1.73E-06	+	1.73E-06	+
B/W/E	30/0/0		28/0/2		28/1/1		29/0/1	

Table C5. Mean and standard deviation of the WOA variant algorithms for IEEE CEC2017, Related to Figure 9 to 11.

Fun	Items	SWEWOA	CWOA	BMWOA	CCMWOA	ACWOA	MWOA	OBWOA
F1	Avg	**1.01E+04**	2.38E+09	2.18E+08	1.92E+10	5.35E+09	8.59E+10	8.53E+09
	Stdv	**6.88E+03**	1.68E+09	9.09E+07	3.99E+09	2.65E+09	1.71E+10	5.86E+09
F2	Avg	**3.57E+02**	3.02E+33	4.17E+23	8.88E+38	1.52E+34	1.14E+50	1.43E+34
	Stdv	**4.18E+02**	1.46E+34	1.46E+24	4.59E+39	4.50E+34	4.28E+50	4.29E+34
F3	Avg	**3.00E+02**	1.79E+05	6.95E+04	7.65E+04	5.11E+04	1.16E+07	5.20E+04
	Stdv	**8.78E-02**	4.79E+04	9.45E+03	5.06E+03	9.21E+03	2.72E+07	1.03E+04
F4	Avg	**4.87E+02**	8.59E+02	5.98E+02	3.38E+03	1.36E+03	2.69E+04	1.59E+03
	Stdv	**1.67E+01**	5.89E+02	4.41E+01	1.31E+03	8.94E+02	1.13E+04	1.04E+03
F5	Avg	**5.99E+02**	8.23E+02	8.04E+02	8.31E+02	8.08E+02	1.06E+03	8.02E+02
	Stdv	2.93E+01	6.27E+01	3.05E+01	3.76E+01	**2.63E+01**	6.47E+01	2.84E+01

F6	Avg	**6.09E+02**	6.74E+02	6.69E+02	6.70E+02	6.65E+02	7.18E+02	6.72E+00
	Stdv	7.37E+00	9.98E+00	7.81E+00	1.01E+01	**6.74E+00**	1.51E+01	3.51E+01
F7	Avg	**8.36E+02**	1.27E+03	**1.24E+03**	1.26E+03	1.24E+03	2.14E+03	1.30E+03
	Stdv	**3.51E+01**	8.06E+01	1.04E+02	8.79E+01	7.68E+01	3.28E+02	7.17E+01
F8	Avg	**9.04E+02**	9.98E+02	**1.00E+03**	1.04E+03	1.01E+03	1.28E+03	1.01E+03
	Stdv	2.97E+01	4.88E+01	2.89E+01	2.84E+01	**2.26E+01**	7.48E+01	2.57E+01
F9	Avg	**2.89E+03**	8.47E+03	7.55E+03	7.69E+03	7.46E+03	2.60E+04	7.11E+03
	Stdv	1.62E+03	2.88E+03	1.26E+03	1.31E+03	1.12E+03	6.82E+03	**8.71E+02**
F10	Avg	**4.51E+03**	6.28E+03	**7.58E+03**	6.99E+03	6.71E+03	1.03E+04	6.52E+03
	Stdv	6.79E+02	7.16E+02	5.73E+02	**4.51E+02**	8.75E+02	5.02E+02	9.38E+02
F11	Avg	**1.27E+03**	**3.55E+03**	1.69E+03	2.98E+03	3.19E+03	4.34E+04	2.38E+03
	Stdv	**5.60E+01**	1.46E+03	2.60E+02	5.53E+02	8.73E+02	1.82E+04	8.51E+02
F12	Avg	**3.14E+06**	1.15E+08	5.77E+07	2.36E+09	5.05E+08	1.78E+10	5.66E+08
	Stdv	**2.16E+06**	1.71E+08	3.87E+07	1.79E+09	3.77E+08	6.11E+09	1.19E+09
F13	Avg	2.14E+05	**1.31E+05**	2.81E+05	1.07E+08	4.19E+07	9.91E+09	2.27E+07
	Stdv	**1.04E+05**	1.16E+05	1.62E+05	2.88E+08	7.22E+07	6.97E+09	6.28E+07
F14	Avg	**1.61E+04**	1.65E+06	6.60E+05	1.65E+06	1.08E+06	1.84E+07	1.27E+06
	Stdv	**7.86E+03**	1.45E+06	5.59E+05	1.25E+06	1.03E+06	1.45E+07	7.40E+05
F15	Avg	**3.62E+04**	3.76E+06	1.63E+05	6.73E+06	6.37E+06	3.34E+09	1.37E+06
	Stdv	**2.04E+04**	1.32E+07	1.88E+05	8.51E+06	4.01E+06	1.98E+09	2.44E+06
F16	Avg	**2.58E+03**	3.67E+03	3.38E+03	3.93E+03	3.92E+03	7.11E+03	3.87E+03
	Stdv	**3.34E+02**	4.86E+02	4.36E+02	6.65E+02	3.59E+02	1.34E+03	5.10E+02
F17	Avg	**2.20E+03**	2.56E+03	2.48E+03	2.80E+03	2.32E+03	1.44E+04	2.63E+03
	Stdv	**1.46E+02**	2.98E+02	2.16E+02	4.55E+02	2.83E+02	2.33E+04	3.90E+02
F18	Avg	**2.35E+05**	3.58E+06	3.84E+06	9.92E+06	4.86E+06	2.24E+08	2.93E+06
	Stdv	**2.23E+05**	3.88E+06	5.70E+06	9.73E+06	5.76E+06	1.87E+08	3.35E+06
F19	Avg	**1.04E+04**	3.78E+06	3.90E+05	3.61E+06	8.43E+06	3.29E+09	1.15E+06
	Stdv	**9.09E+03**	7.15E+06	4.19E+05	4.55E+06	1.13E+07	2.23E+09	9.55E+05
F20	Avg	**2.40E+03**	2.77E+03	2.70E+03	2.64E+03	2.66E+03	3.59E+03	2.73E+03
	Stdv	1.93E+02	1.94E+02	1.95E+02	2.03E+02	**1.62E+02**	1.82E+02	1.92E+02
F21	Avg	**2.41E+03**	2.60E+03	2.53E+03	2.62E+03	2.58E+03	2.83E+03	2.60E+03
	Stdv	**2.17E+01**	6.44E+01	4.07E+01	4.41E+01	3.95E+01	6.23E+01	4.56E+01
F22	Avg	**3.37E+03**	7.41E+03	5.02E+03	7.35E+03	5.15E+03	1.13E+04	7.35E+03
	Stdv	1.84E+03	1.33E+03	3.15E+03	1.34E+03	2.29E+03	**1.10E+03**	1.50E+03
F23	Avg	**2.76E+03**	3.10E+03	2.96E+03	3.16E+03	3.07E+03	3.54E+03	3.07E+03
	Stdv	**3.33E+01**	1.07E+02	8.76E+01	1.20E+02	9.45E+01	1.71E+02	1.12E+02
F24	Avg	**2.91E+03**	3.22E+03	3.14E+03	3.31E+03	3.21E+03	3.80E+03	3.18E+03
	Stdv	**2.47E+01**	1.11E+02	7.21E+01	1.07E+02	7.20E+01	1.90E+02	9.78E+01
F25	Avg	**2.89E+03**	3.05E+03	3.03E+03	3.37E+03	3.15E+03	9.86E+03	3.12E+03
	Stdv	**2.41E+00**	9.49E+01	3.26E+01	1.33E+02	1.04E+02	3.51E+03	1.07E+02
F26	Avg	**4.32E+03**	7.78E+03	6.34E+03	9.08E+03	7.47E+03	1.27E+04	8.02E+03
	Stdv	1.06E+03	9.16E+02	1.54E+03	**7.02E+02**	1.08E+03	1.57E+03	1.23E+03
F27	Avg	**3.22E+03**	3.41E+03	3.33E+03	3.65E+03	3.47E+03	4.57E+03	3.38E+03

	Stdv	**1.43E+01**	1.10E+02	6.60E+01	1.37E+02	1.04E+02	4.48E+02	9.15E+01
F28	Avg	**3.21E+03**	3.60E+03	3.41E+03	4.51E+03	3.83E+03	9.53E+03	3.76E+03
	Stdv	**4.26E+01**	2.86E+02	5.11E+01	3.73E+02	2.67E+02	1.62E+03	3.03E+02
F29	Avg	**3.79E+03**	4.94E+03	4.76E+03	5.33E+03	4.76E+03	1.05E+04	4.78E+03
	Stdv	**2.21E+02**	4.30E+02	4.17E+02	5.35E+02	4.03E+02	9.26E+03	4.17E+02
F30	Avg	**2.31E+05**	1.36E+07	5.91E+06	1.19E+08	6.80E+07	2.32E+09	8.52E+07
	Stdv	**9.29E+04**	9.18E+06	3.97E+06	1.05E+08	4.46E+07	1.12E+09	2.23E+08

Table C6. Comparative results of the WOA variant algorithms for IEEE CEC2017, Related to Figure 9 to 11.

Fun	CWOA		BMWOA		CCMWOA		ACWOA		MWOA		OBWOA	
	P-value	Results	P-value	Results	P-value	Results	P-value	Results	P-value	Results	P-value	Results
F1	1.73E-06	+	1.73E-06	+	1.73E-06	+	1.73E-06	+	1.73E-06	+	1.73E-06	+
F2	1.73E-06	+	1.73E-06	+	1.73E-06	+	1.73E-06	+	1.73E-06	+	1.73E-06	+
F3	1.73E-06	+	1.73E-06	+	1.73E-06	+	1.73E-06	+	1.73E-06	+	1.73E-06	+
F4	1.73E-06	+	1.73E-06	+	1.73E-06	+	1.73E-06	+	1.73E-06	+	1.73E-06	+
F5	1.73E-06	+	1.73E-06	+	1.73E-06	+	1.73E-06	+	1.73E-06	+	1.73E-06	+
F6	1.73E-06	+	1.73E-06	+	1.73E-06	+	1.73E-06	+	1.73E-06	+	1.73E-06	+
F7	1.73E-06	+	1.73E-06	+	1.73E-06	+	1.73E-06	+	1.73E-06	+	1.73E-06	+
F8	2.12E-06	+	1.73E-06	+	1.73E-06	+	1.73E-06	+	1.73E-06	+	1.92E-06	+
F9	3.52E-06	+	1.73E-06	+	2.35E-06	+	1.73E-06	+	1.73E-06	+	1.92E-06	+
F10	2.88E-06	+	1.73E-06	+	1.73E-06	+	1.73E-06	+	1.73E-06	+	2.35E-06	+
F11	1.73E-06	+	1.73E-06	+	1.73E-06	+	1.73E-06	+	1.73E-06	+	1.73E-06	+
F12	1.73E-06	+	1.73E-06	+	1.73E-06	+	1.73E-06	+	1.73E-06	+	1.73E-06	+
F13	2.26E-03	-	7.86E-02		1.73E-06	+	1.73E-06	+	1.73E-06	+	8.94E-01	
F14	1.73E-06	+	1.73E-06	+	1.73E-06	+	1.73E-06	+	1.73E-06	+	1.73E-06	+
F15	2.60E-05	+	2.41E-04	+	1.73E-06	+	1.73E-06	+	1.73E-06	+	1.92E-06	+
F16	1.92E-06	+	4.73E-06	+	2.13E-06	+	1.73E-06	+	1.73E-06	+	1.73E-06	+
F17	9.32E-06	+	2.37E-05	+	2.60E-06	+	1.36E-05	+	1.73E-06	+	6.32E-05	+
F18	1.92E-06	+	3.88E-06	+	1.92E-06	+	2.88E-06	+	1.73E-06	+	1.64E-05	+
F19	1.73E-06	+	1.73E-06	+	1.73E-06	+	1.73E-06	+	1.73E-06	+	1.73E-06	+
F20	2.13E-06	+	6.32E-05	+	6.16E-04	+	4.86E-05	+	1.73E-06	+	1.36E-05	+
F21	1.73E-06	+	1.73E-06	+	1.73E-06	+	1.73E-06	+	1.73E-06	+	1.73E-06	+
F22	6.98E-06	+	8.22E-03	+	6.98E-06	+	1.48E-02	+	1.73E-06	+	8.47E-06	+
F23	1.73E-06	+	1.92E-06	+	1.73E-06	+	1.73E-06	+	1.73E-06	+	1.73E-06	+
F24	1.73E-06	+	1.73E-06	+	1.73E-06	+	1.73E-06	+	1.73E-06	+	1.73E-06	+
F25	1.73E-06	+	1.73E-06	+	1.73E-06	+	1.73E-06	+	1.73E-06	+	1.73E-06	+
F26	1.92E-06	+	5.31E-05	+	1.73E-06	+	2.13E-06	+	1.73E-06	+	2.13E-06	+
F27	1.73E-06	+	1.73E-06	+	1.73E-06	+	1.73E-06	+	1.73E-06	+	1.73E-06	+
F28	1.73E-06	+	1.73E-06	+	1.73E-06	+	1.73E-06	+	1.73E-06	+	1.73E-06	+
F29	1.73E-06	+	1.73E-06	+	1.73E-06	+	1.73E-06	+	1.73E-06	+	2.60E-06	+
F30	1.73E-06	+	1.73E-06	+	1.73E-06	+	1.73E-06	+	1.73E-06	+	1.73E-06	+
B/W/E	29/0/1		29/0/1		30/0/0		30/0/0		30/0/0		29/0/1	

Table C7. Mean and standard deviation of the other variant algorithms for IEEE CEC2017, Related to Figure 12 to 14.

Fun	Items	SWEWOA	ASCA_PSO	SCADE	MSFOA	GWOSCA	HGWO	CMFO
F1	Avg	**8.65E+03**	7.84E+08	2.04E+10	1.00E+11	8.07E+10	8.13E+09	2.82E+08
	Stdv	4.89E+03	1.32E+09	2.93E+09	1.67E+10	**1.23E-01**	1.04E+09	4.52E+08
F2	Avg	**2.42E+02**	3.29E+30	2.90E+36	3.53E+53	3.83E+56	8.42E+33	6.12E+37
	Stdv	**1.23E+02**	1.13E+31	5.53E+36	1.91E+54	1.99E+57	1.77E+34	2.72E+38
F3	Avg	**3.00E+02**	1.44E+03	5.89E+04	2.75E+05	9.42E+04	7.61E+04	1.15E+05
	Stdv	**9.71E-02**	7.08E+02	6.15E+03	6.71E+04	3.22E-06	6.69E+03	4.86E+04
F4	Avg	**4.90E+02**	5.71E+02	3.64E+03	3.56E+04	2.34E+04	9.08E+02	6.69E+02
	Stdv	**2.10E+01**	1.17E+02	7.90E+02	1.20E+04	6.15E+02	1.00E+02	2.10E+02
F5	Avg	**6.09E+02**	7.26E+02	8.30E+02	1.07E+03	1.01E+03	7.46E+02	7.17E+02
	Stdv	3.67E+01	3.18E+01	1.98E+01	7.33E+01	**7.73E-01**	1.21E+01	4.17E+01
F6	Avg	**6.09E+02**	6.35E+02	6.63E+02	7.15E+02	7.11E+02	6.37E+02	6.49E+02
	Stdv	8.89E+00	1.20E+01	6.39E+00	1.70E+01	**7.39E-03**	4.60E+00	9.25E+00
F7	Avg	**8.35E+02**	9.93E+02	1.16E+03	2.76E+03	1.59E+03	1.05E+03	1.27E+03
	Stdv	2.57E+01	3.48E+01	3.74E+01	2.87E+02	**6.20E-09**	2.04E+01	1.08E+02
F8	Avg	**9.19E+02**	1.01E+03	1.08E+03	1.30E+03	1.24E+03	1.00E+03	9.64E+02
	Stdv	3.13E+01	3.19E+01	1.47E+01	5.07E+01	**3.80E-08**	1.37E+01	3.27E+01
F9	Avg	**2.48E+03**	4.64E+03	8.28E+03	2.27E+04	1.75E+04	3.44E+03	5.08E+03
	Stdv	1.30E+03	1.26E+03	1.14E+03	4.85E+03	**1.41E-06**	3.77E+02	1.09E+03
F10	Avg	**4.57E+03**	6.07E+03	8.20E+03	9.63E+03	1.07E+04	6.62E+03	6.84E+03
	Stdv	4.93E+02	8.62E+02	**2.54E+02**	7.00E+02	2.66E+02	2.70E+02	1.32E+03
F11	Avg	**1.27E+03**	1.31E+03	3.31E+03	3.31E+04	1.62E+04	4.56E+03	3.64E+03
	Stdv	5.13E+01	**4.84E+01**	6.56E+02	1.99E+04	4.29E-05	9.97E+02	1.86E+03
F12	Avg	**3.78E+06**	6.15E+07	2.15E+09	2.14E+10	2.45E+10	6.26E+08	3.36E+07
	Stdv	**3.58E+06**	1.15E+08	5.00E+08	5.82E+09	3.73E+09	1.40E+08	1.15E+08
F13	Avg	2.10E+05	1.08E+07	5.59E+08	1.63E+10	3.01E+10	2.58E+08	**2.49E+04**
	Stdv	1.06E+05	1.28E+07	2.46E+08	8.38E+09	4.06E+09	1.14E+08	**2.76E+04**
F14	Avg	**1.41E+04**	3.95E+04	2.80E+05	1.83E+07	1.04E+08	8.63E+05	2.88E+05
	Stdv	**6.38E+03**	3.15E+04	1.49E+05	2.05E+07	8.78E+07	5.27E+05	6.87E+05
F15	Avg	**3.89E+04**	1.05E+06	1.02E+07	3.70E+09	4.41E+09	1.11E+07	4.29E+04
	Stdv	**2.83E+04**	3.81E+05	1.19E+07	2.83E+09	4.04E+08	1.35E+07	5.90E+04
F16	Avg	**2.56E+03**	3.01E+03	3.87E+03	6.65E+03	1.06E+04	3.34E+03	2.97E+03
	Stdv	2.95E+02	**2.21E+02**	2.92E+02	1.69E+03	2.71E+03	2.71E+02	3.95E+02
F17	Avg	**2.15E+03**	2.23E+03	2.51E+03	7.02E+03	6.26E+04	2.33E+03	2.40E+03
	Stdv	1.58E+02	1.65E+02	**1.37E+02**	4.67E+03	7.70E+04	1.39E+02	2.63E+02
F18	Avg	**2.70E+05**	5.57E+05	4.37E+06	2.09E+08	1.38E+09	1.24E+06	5.59E+06
	Stdv	**2.14E+05**	3.55E+05	3.23E+06	2.33E+08	8.31E+08	1.09E+06	1.05E+07
F19	Avg	**8.94E+03**	5.72E+06	2.53E+07	3.92E+09	3.62E+09	1.22E+07	9.36E+04
	Stdv	4.55E+03	3.47E+06	1.26E+07	2.77E+09	**2.97E-01**	1.20E+07	2.15E+05
F20	Avg	**2.42E+03**	2.50E+03	2.71E+03	3.40E+03	3.72E+03	2.64E+03	2.77E+03
	Stdv	1.86E+02	1.54E+02	9.37E+01	2.33E+02	**5.05E+01**	1.14E+02	2.43E+02

F21	Avg	**2.41E+03**	2.48E+03	2.58E+03	2.83E+03	2.96E+03	2.51E+03	2.49E+03
	Stdv	5.27E+01	2.74E+01	2.39E+01	6.23E+01	7.94E+01	**1.53E+01**	5.71E+01
F22	Avg	3.20E+03	6.35E+03	4.62E+03	1.10E+04	1.17E+04	**3.20E+03**	7.32E+03
	Stdv	1.71E+03	2.16E+03	3.29E+02	5.45E+02	**1.61E+02**	2.06E+02	2.04E+03
F23	Avg	**2.76E+03**	2.87E+03	3.01E+03	3.80E+03	4.11E+03	2.90E+03	2.96E+03
	Stdv	2.95E+01	4.03E+01	2.10E+01	2.10E+02	2.55E+02	**1.36E+01**	8.08E+01
F24	Avg	**2.92E+03**	3.05E+03	3.18E+03	4.11E+03	4.43E+03	3.06E+03	3.14E+03
	Stdv	**2.62E+01**	4.92E+01	2.65E+01	2.58E+02	2.62E+02	3.57E+01	1.17E+02
F25	Avg	**2.89E+03**	2.93E+03	3.42E+03	1.31E+04	7.21E+03	3.09E+03	2.95E+03
	Stdv	**1.18E+01**	3.02E+01	9.40E+01	3.45E+03	3.11E-07	4.07E+01	3.60E+01
F26	Avg	**4.13E+03**	6.34E+03	7.37E+03	1.45E+04	1.52E+04	6.01E+03	6.88E+03
	Stdv	9.24E+02	4.20E+02	4.00E+02	1.79E+03	5.87E+02	**3.43E+02**	8.82E+02
F27	Avg	3.22E+03	3.31E+03	3.44E+03	**3.20E+03**	5.83E+03	3.31E+03	3.38E+03
	Stdv	**1.17E+01**	4.51E+01	4.63E+01	2.94E-06	6.33E+02	2.76E+01	9.25E+01
F28	Avg	**3.21E+03**	3.33E+03	4.32E+03	3.30E+03	9.55E+03	3.59E+03	3.38E+03
	Stdv	**4.61E+01**	1.68E+02	2.99E+02	3.55E-09	2.78E-08	4.75E+01	2.00E+02
F29	Avg	**3.75E+03**	4.38E+03	5.18E+03	1.11E+04	7.33E+04	4.47E+03	4.50E+03
	Stdv	2.05E+02	2.09E+02	2.10E+02	5.24E+03	5.21E+04	**1.61E+02**	3.32E+02
F30	Avg	**2.25E+05**	7.92E+06	1.22E+08	2.39E+09	5.82E+09	8.82E+07	5.50E+05
	Stdv	**1.29E+05**	3.72E+06	5.02E+07	1.49E+09	2.17E+09	2.64E+07	1.84E+06

Table C8. Comparative results of the other variant algorithms for IEEE CEC2017, Related to Figure 12 to 14.

Fun	ASCA_PSO		SCADE		MSFOA		GWOSCA		HGWO		CMFO	
	P-value	Results	P-value	Results	P-value	Results	P-value	Results	P-value	Results	P-value	Results
F1	1.73E-06	+	1.73E-06	+	1.73E-06	+	1.73E-06	+	1.73E-06	+	1.73E-06	+
F2	1.73E-06	+	1.73E-06	+	1.73E-06	+	1.73E-06	+	1.73E-06	+	1.73E-06	+
F3	1.73E-06	+	1.73E-06	+	1.73E-06	+	1.73E-06	+	1.73E-06	+	1.73E-06	+
F4	2.05E-04	+	1.73E-06	+	1.73E-06	+	1.73E-06	+	1.73E-06	+	1.73E-06	+
F5	1.92E-06	+	1.73E-06	+	1.73E-06	+	1.73E-06	+	1.73E-06	+	1.73E-06	+
F6	9.32E-06	+	1.73E-06	+	1.73E-06	+	1.73E-06	+	1.92E-06	+	1.73E-06	+
F7	1.73E-06	+	1.73E-06	+	1.73E-06	+	1.73E-06	+	1.73E-06	+	1.73E-06	+
F8	1.73E-06	+	1.73E-06	+	1.73E-06	+	1.73E-06	+	1.73E-06	+	8.92E-05	+
F9	1.97E-05	+	1.73E-06	+	1.73E-06	+	1.73E-06	+	5.71E-04	+	5.22E-06	+
F10	1.73E-06	+	1.73E-06	+	1.73E-06	+	1.73E-06	+	1.73E-06	+	1.92E-06	+
F11	6.84E-03	+	1.73E-06	+	1.73E-06	+	1.73E-06	+	1.73E-06	+	1.73E-06	+
F12	1.73E-06	+	1.73E-06	+	1.73E-06	+	1.73E-06	+	1.73E-06	+	1.85E-02	+
F13	1.73E-06	+	1.73E-06	+	1.73E-06	+	1.73E-06	+	1.73E-06	+	2.35E-06	-
F14	1.15E-04	+	1.73E-06	+	1.73E-06	+	1.73E-06	+	1.73E-06	+	5.79E-05	+
F15	1.73E-06	+	1.73E-06	+	1.73E-06	+	1.73E-06	+	1.73E-06	+	5.04E-01	
F16	6.34E-06	+	1.73E-06	+	1.73E-06	+	1.73E-06	+	3.52E-06	+	3.32E-04	+
F17	4.07E-02	+	1.73E-06	+	1.73E-06	+	1.73E-06	+	5.71E-04	+	4.53E-04	+
F18	6.16E-04	+	1.73E-06	+	1.73E-06	+	1.73E-06	+	1.73E-06	+	5.29E-04	+
F19	1.73E-06	+	1.73E-06	+	1.73E-06	+	1.73E-06	+	1.73E-06	+	4.49E-02	+

F20	2.06E-01		8.47E-06	+	1.73E-06	+	1.73E-06	+	2.61E-04	+	3.52E-06	+
F21	8.47E-06	+	1.73E-06	+	1.73E-06	+	1.73E-06	+	1.73E-06	+	2.37E-05	+
F22	7.69E-06	+	8.94E-04	+	1.73E-06	+	1.73E-06	+	3.71E-01		9.32E-06	+
F23	2.60E-06	+	1.73E-06	+	1.73E-06	+	1.73E-06	+	1.73E-06	+	1.73E-06	+
F24	1.92E-06	+	1.73E-06	+	1.73E-06	+	1.73E-06	+	1.73E-06	+	1.73E-06	+
F25	2.88E-06	+	1.73E-06	+	1.73E-06	+	1.73E-06	+	1.73E-06	+	4.73E-06	+
F26	1.73E-06	+	1.73E-06	+	1.73E-06	+	1.73E-06	+	1.73E-06	+	1.73E-06	+
F27	1.73E-06	+	1.73E-06	+	1.73E-06	-	1.73E-06	+	1.73E-06	+	1.73E-06	+
F28	1.64E-05	+	1.73E-06	+	1.73E-06	+	1.73E-06	+	1.73E-06	+	1.73E-06	+
F29	2.35E-06	+	1.73E-06	+	1.73E-06	+	1.73E-06	+	1.73E-06	+	1.92E-06	+
F30	1.73E-06	+	1.73E-06	+	1.73E-06	+	1.73E-06	+	1.73E-06	+	3.71E-01	
B/W/E	29/0/1		30/0/0		29/1/0		30/0/0		29/0/1		27/1/2	

Table C9. Mean and standard deviation of the comparative experiment of the algorithms for IEEE CEC2022, Related to Figure 15.

Fun	Items	SWEWOA	WDNMWOA	BWOA	FSTPSO	DHHOM	GWO	BA
F31	Avg	**3.00E+02**	3.04E+02	4.35E+03	2.41E+04	3.74E+02	8.67E+03	3.00E+02
	Stdv	**2.68E-03**	8.07E+00	2.17E+03	8.53E+03	7.59E+01	4.37E+03	7.60E-02
F32	Avg	4.47E+02	4.50E+02	5.15E+02	7.87E+02	4.63E+02	4.88E+02	**4.37E+02**
	Stdv	1.69E+01	2.13E+01	4.18E+01	**1.20E+02**	1.45E+01	3.50E+01	2.71E+01
F33	Avg	**6.02E+02**	6.49E+02	6.62E+02	6.53E+02	6.54E+02	6.04E+02	6.71E+02
	Stdv	**2.69E+00**	1.38E+01	9.54E+00	1.20E+01	9.00E+00	3.23E+00	1.15E+01
F34	Avg	1.90E+03	1.91E+03	**1.90E+03**	1.98E+03	1.90E+03	1.90E+03	1.91E+03
	Stdv	5.83E-03	2.78E+00	**0.00E+00**	8.52E+01	0.00E+00	1.20E-01	2.78E+00
F35	Avg	**1.09E+03**	3.00E+03	2.57E+03	2.30E+03	2.65E+03	1.17E+03	5.32E+03
	Stdv	5.11E+02	1.01E+03	2.79E+02	5.36E+02	**2.57E+02**	2.64E+02	2.31E+03
F36	Avg	**6.18E+03**	8.84E+03	7.63E+04	3.99E+06	7.19E+04	6.80E+05	7.15E+04
	Stdv	**6.14E+03**	6.72E+03	1.16E+05	9.55E+06	3.20E+04	3.29E+06	3.25E+04
F37	Avg	**2.06E+03**	2.14E+03	2.17E+03	2.18E+03	2.15E+03	2.08E+03	2.23E+03
	Stdv	**1.88E+01**	4.02E+01	3.81E+01	5.61E+01	3.86E+01	4.26E+01	6.50E+01
F38	Avg	**2.23E+03**	2.24E+03	2.25E+03	2.36E+03	2.25E+03	2.24E+03	2.50E+03
	Stdv	1.09E+01	**9.74E+00**	3.18E+01	1.07E+02	3.12E+01	4.10E+01	1.41E+02
F39	Avg	**2.48E+03**	2.48E+03	2.51E+03	2.68E+03	2.49E+03	2.50E+03	2.48E+03
	Stdv	**1.93E-03**	8.29E-02	2.45E+01	6.87E+01	3.56E+00	2.01E+01	2.66E-03
F40	Avg	**2.95E+03**	3.10E+03	3.75E+03	5.08E+03	3.37E+03	3.04E+03	4.58E+03
	Stdv	6.25E+02	**5.50E+02**	1.06E+03	1.02E+03	6.02E+02	5.77E+02	1.12E+03
F41	Avg	**2.66E+03**	2.78E+03	2.75E+03	2.98E+03	2.85E+03	2.74E+03	2.85E+03
	Stdv	**1.24E+02**	1.67E+02	1.39E+02	3.19E+02	1.87E+02	1.47E+02	1.46E+02
F42	Avg	**2.95E+03**	3.01E+03	3.01E+03	3.33E+03	3.08E+03	2.97E+03	3.09E+03
	Stdv	**1.48E+01**	4.65E+01	5.31E+01	2.62E+02	1.07E+02	1.77E+01	1.25E+02

Table C10. Comparative results of the algorithms for IEEE CEC2022, Related to Table 8 to 9.

Fun	WDNMWOA		BWOA		FSTPSO		DHHOM		GWO		BA	
	P-value	Results	P-value	Results	P-value	Results	P-value	Results	P-value	Results	P-value	Results
F31	1.73E-06	+	1.73E-06	+	1.73E-06	+	1.73E-06	+	1.73E-06	+	1.92E-06	+

F32	2.71E-01		1.73E-06	+	1.73E-06	+	5.79E-05	+	9.32E-06	+	3.71E-01	
F33	1.73E-06	+	1.73E-06	+	1.73E-06	+	1.73E-06	+	1.38E-03	+	1.73E-06	+
F34	1.73E-06	+	1.73E-06	-	1.73E-06	+	1.73E-06	-	3.11E-05	+	1.73E-06	+
F35	1.92E-06	+	2.13E-06	+	5.75E-06	+	2.13E-06	+	7.27E-03	+	1.73E-06	+
F36	5.71E-02		3.88E-06	+	2.13E-06	+	1.73E-06	+	2.26E-03	+	1.73E-06	+
F37	1.73E-06	+	1.73E-06	+	1.73E-06	+	1.73E-06	+	5.32E-03	+	1.73E-06	+
F38	5.19E-02		2.22E-04	+	5.75E-06	+	4.95E-02	+	3.29E-01		1.92E-06	+
F39	5.75E-06	+	1.73E-06	+	1.73E-06	+	1.73E-06	+	1.73E-06	+	1.40E-02	+
F40	1.59E-01		5.31E-05	+	2.35E-06	+	3.50E-02	+	3.82E-01		1.97E-05	+
F41	2.85E-02	+	2.22E-04	+	1.36E-05	+	8.92E-05	+	4.72E-02	+	4.45E-05	+
F42	3.18E-06	+	2.88E-06	+	1.73E-06	+	1.92E-06	+	1.17E-02	+	2.35E-06	+
B/W/E	8/0/4		11/1/0		12/0/0		11/1/0		10/0/2		11/0/1	

Table C11. Comparison between BSWEWOA and other algorithms on Specificity of KELM, Related to Table 13 to 16.

Dataset	Items	BSWEWOA	BGWO	BGSA	BPSO	BBA	BSSA	BWOA
BreastEW	Avg	**9.71E-01**	9.62E-01	9.61E-01	9.67E-01	9.67E-01	9.62E-01	9.67E-01
	Stdv	**3.33E-02**	3.68E-02	3.68E-02	3.85E-02	5.04E-02	3.68E-02	3.92E-02
clean1	Avg	**1.00E+00**	9.90E-01	9.95E-01	9.85E-01	9.61E-01	9.76E-01	9.86E-01
	Stdv	**0.00E+00**	2.11E-02	1.51E-02	2.34E-02	3.12E-02	3.41E-02	2.30E-02
heartandlung	Avg	**9.86E-01**	9.57E-01	9.71E-01	9.86E-01	9.71E-01	9.57E-01	9.71E-01
	Stdv	**4.52E-02**	6.90E-02	6.02E-02	**4.52E-02**	6.02E-02	6.90E-02	6.02E-02
Breastcancer	Avg	9.88E-01	9.84E-01	**9.92E-01**	9.84E-01	9.84E-01	9.83E-01	9.83E-01
	Stdv	2.81E-02	2.13E-02	**1.76E-02**	2.13E-02	2.13E-02	2.91E-02	2.91E-02
German	Avg	**5.53E-01**	5.43E-01	5.47E-01	5.47E-01	5.30E-01	5.37E-01	5.47E-01
	Stdv	7.06E-02	1.17E-01	9.45E-02	7.73E-02	9.62E-02	8.38E-02	**6.52E-02**
JPNdata	Avg	**9.45E-01**	8.14E-01	9.07E-01	8.96E-01	8.95E-01	8.79E-01	8.64E-01
	Stdv	**7.17E-02**	1.46E-01	1.05E-01	1.46E-01	1.00E-01	1.25E-01	1.12E-01
Vote	Avg	**9.78E-01**	9.62E-01	9.67E-01	9.40E-01	9.68E-01	9.45E-01	9.68E-01
	Stdv	2.87E-02	3.60E-02	4.58E-02	6.61E-02	4.56E-02	6.36E-02	**2.80E-02**
wdbc	Avg	**1.00E+00**	**1.00E+00**	**1.00E+00**	**1.00E+00**	**1.00E+00**	**1.00E+00**	**1.00E+00**
	Stdv	**0.00E+00**	**0.00E+00**	**0.00E+00**	**0.00E+00**	**0.00E+00**	**0.00E+00**	**0.00E+00**
heart	Avg	**9.25E-01**	9.08E-01	9.00E-01	9.00E-01	9.00E-01	8.75E-01	8.83E-01
	Stdv	7.30E-02	8.29E-02	**6.57E-02**	1.02E-01	6.57E-02	1.19E-01	8.96E-02
Parkinson	Avg	**8.80E-01**	7.90E-01	8.35E-01	8.15E-01	8.10E-01	8.55E-01	8.55E-01
	Stdv	**1.03E-01**	1.90E-01	1.29E-01	1.76E-01	2.13E-01	1.92E-01	1.38E-01
SonarEW	Avg	9.91E-01	9.91E-01	9.82E-01	9.73E-01	9.38E-01	9.73E-01	**9.92E-01**
	Stdv	2.87E-02	2.87E-02	3.83E-02	6.14E-02	7.23E-02	4.39E-02	**2.64E-02**
thyroid_2class	Avg	**9.63E-01**	9.34E-01	9.52E-01	8.78E-01	8.68E-01	9.43E-01	9.16E-01
	Stdv	**4.82E-02**	9.92E-02	9.62E-02	9.73E-02	1.37E-01	1.05E-01	7.99E-02
Wielaw	Avg	8.75E-01	8.51E-01	8.83E-01	8.61E-01	8.91E-01	8.21E-01	**9.00E-01**
	Stdv	9.05E-02	1.35E-01	1.16E-01	9.41E-02	**6.60E-02**	1.08E-01	1.41E-01
Mean_rank		**1.38**	4.38	2.77	3.69	4.23	5.08	3.23
Rank		**1**	6	2	4	5	7	3

Table C12. Comparison between BSWEWOA and other algorithms on MCC of KELM, Related to Table 13 to 16.

Dataset	Items	BSWEWOA	BGWO	BGSA	BPSO	BBA	BSSA	BWOA
BreastEW	Avg	**9.77E-01**	9.70E-01	9.70E-01	9.74E-01	9.74E-01	9.70E-01	9.74E-01
	Stdv	**2.62E-02**	2.89E-02	2.90E-02	3.02E-02	3.91E-02	2.89E-02	3.06E-02
clean1	Avg	**9.96E-01**	9.79E-01	9.55E-01	9.70E-01	9.07E-01	9.13E-01	9.62E-01
	Stdv	**1.30E-02**	3.05E-02	5.27E-02	3.48E-02	5.21E-02	6.08E-02	4.17E-02
heartandlung	Avg	**9.87E-01**	9.45E-01	9.46E-01	9.73E-01	9.46E-01	9.60E-01	9.73E-01
	Stdv	**4.24E-02**	9.83E-02	6.92E-02	5.65E-02	6.92E-02	6.47E-02	5.65E-02
Breastcancer	Avg	**9.72E-01**	9.63E-01	9.66E-01	9.69E-01	9.69E-01	9.63E-01	9.66E-01
	Stdv	2.68E-02	2.41E-02	3.73E-02	3.59E-02	3.26E-02	2.87E-02	**2.29E-02**
German	Avg	**5.78E-01**	5.71E-01	5.70E-01	5.69E-01	5.38E-01	5.66E-01	5.73E-01
	Stdv	**4.86E-02**	7.17E-02	6.19E-02	7.71E-02	9.10E-02	7.07E-02	5.28E-02
JPNdata	Avg	**8.73E-01**	7.91E-01	8.53E-01	8.10E-01	8.27E-01	7.95E-01	8.29E-01
	Stdv	1.33E-01	1.43E-01	9.65E-02	1.27E-01	1.00E-01	1.43E-01	**8.40E-02**
Vote	Avg	**9.73E-01**	9.33E-01	9.54E-01	9.30E-01	9.47E-01	9.35E-01	9.53E-01
	Stdv	3.51E-02	4.49E-02	5.32E-02	7.51E-02	6.09E-02	7.43E-02	**3.26E-02**
wdbc	Avg	**9.67E-01**	9.63E-01	9.59E-01	9.59E-01	9.59E-01	9.59E-01	9.66E-01
	Stdv	3.62E-02	3.41E-02	**2.71E-02**	3.69E-02	3.25E-02	2.75E-02	2.75E-02
heart	Avg	8.90E-01	**8.91E-01**	8.68E-01	8.77E-01	8.76E-01	8.31E-01	8.61E-01
	Stdv	**6.35E-02**	9.25E-02	6.83E-02	9.78E-02	7.04E-02	1.36E-01	1.01E-01
Parkinson	Avg	**9.06E-01**	8.57E-01	8.52E-01	8.32E-01	8.44E-01	8.88E-01	8.76E-01
	Stdv	9.13E-02	1.33E-01	**7.84E-02**	1.30E-01	1.60E-01	1.45E-01	1.19E-01
SonarEW	Avg	**9.82E-01**	9.81E-01	9.53E-01	9.44E-01	8.98E-01	9.44E-01	9.72E-01
	Stdv	**3.85E-02**	3.95E-02	6.66E-02	7.85E-02	1.02E-01	4.81E-02	6.29E-02
thyroid_2class	Avg	**8.13E-01**	7.48E-01	7.79E-01	7.78E-01	7.61E-01	7.66E-01	7.42E-01
	Stdv	1.28E-01	**8.38E-02**	1.03E-01	8.85E-02	1.25E-01	1.18E-01	1.11E-01
Wielaw	Avg	**7.80E-01**	7.38E-01	7.54E-01	7.42E-01	7.16E-01	6.99E-01	7.51E-01
	Stdv	1.37E-01	1.39E-01	1.60E-01	**8.90E-02**	1.15E-01	1.13E-01	1.14E-01
Mean_rank		**1.08**	4.14	3.85	4.23	4.92	5.62	3.62
Rank		**1**	4	3	5	6	7	2

Table C13. Comparison between BSWEWOA and other algorithms on F-measure of KELM, Related to Table 13 to 16.

Dataset	Items	BSWEWOA	BGWO	BGSA	BPSO	BBA	BSSA	BWOA
BreastEW	Avg	**9.92E-01**	9.89E-01	9.89E-01	9.90E-01	9.91E-01	9.89E-01	9.90E-01
	Stdv	**9.71E-03**	1.07E-02	1.08E-02	1.12E-02	1.42E-02	1.07E-02	1.12E-02
clean1	Avg	**9.98E-01**	9.91E-01	9.79E-01	9.87E-01	9.59E-01	9.60E-01	9.83E-01
	Stdv	**5.97E-03**	1.31E-02	2.52E-02	1.53E-02	2.33E-02	2.76E-02	1.87E-02
heartandlung	Avg	**9.93E-01**	9.72E-01	9.71E-01	9.86E-01	9.71E-01	9.80E-01	9.87E-01
	Stdv	**2.11E-02**	4.91E-02	3.72E-02	3.04E-02	3.72E-02	3.22E-02	2.81E-02
Breastcancer	Avg	**9.90E-01**	9.87E-01	9.88E-01	9.89E-01	9.89E-01	9.87E-01	9.88E-01
	Stdv	9.60E-03	8.80E-03	1.33E-02	1.27E-02	1.16E-02	1.02E-02	**8.01E-03**
German	Avg	**8.86E-01**	8.86E-01	8.85E-01	8.85E-01	8.78E-01	8.85E-01	8.86E-01

	Stdv	1.43E-02	**1.39E-02**	1.63E-02	1.88E-02	2.02E-02	1.66E-02	1.42E-02
JPNdata	Avg	**9.31E-01**	8.97E-01	9.22E-01	8.95E-01	9.09E-01	8.96E-01	9.12E-01
	Stdv	7.39E-02	7.02E-02	5.30E-02	6.82E-02	5.13E-02	6.32E-02	**4.42E-02**
Vote	Avg	**9.83E-01**	9.58E-01	9.72E-01	9.56E-01	9.66E-01	9.60E-01	9.71E-01
	Stdv	2.16E-02	2.83E-02	3.32E-02	4.67E-02	4.02E-02	4.66E-02	**2.03E-02**
wdbc	Avg	**9.78E-01**	9.76E-01	9.73E-01	9.73E-01	9.73E-01	9.73E-01	9.78E-01
	Stdv	2.44E-02	2.29E-02	**1.82E-02**	2.53E-02	2.20E-02	1.85E-02	1.84E-02
heart	Avg	9.51E-01	**9.52E-01**	9.41E-01	9.45E-01	9.45E-01	9.26E-01	9.39E-01
	Stdv	**2.70E-02**	4.12E-02	3.05E-02	4.22E-02	3.14E-02	5.68E-02	4.37E-02
Parkinson	Avg	**9.77E-01**	9.68E-01	9.63E-01	9.60E-01	9.64E-01	9.74E-01	9.70E-01
	Stdv	2.34E-02	2.94E-02	**1.92E-02**	3.02E-02	3.53E-02	3.30E-02	2.92E-02
SonarEW	Avg	**9.90E-01**	9.89E-01	9.75E-01	9.69E-01	9.46E-01	9.69E-01	9.83E-01
	Stdv	**2.12E-02**	2.22E-02	3.56E-02	4.25E-02	5.29E-02	2.70E-02	4.07E-02
thyroid_2class	Avg	**8.78E-01**	8.29E-01	8.46E-01	8.65E-01	8.57E-01	8.42E-01	8.29E-01
	Stdv	8.44E-02	7.31E-02	8.06E-02	**5.88E-02**	7.07E-02	9.14E-02	1.08E-01
Wielaw	Avg	**8.82E-01**	8.55E-01	8.66E-01	8.60E-01	8.34E-01	8.35E-01	8.52E-01
	Stdv	7.38E-02	7.50E-02	8.17E-02	**4.82E-02**	7.15E-02	6.86E-02	7.19E-02
Mean_rank		**1.08**	4.08	4.38	4.38	4.92	5.31	3.62
Rank		**1**	3	4	4	6	7	2
